# The Impact of Polymerase Chain Reaction Urine Testing on Clinical Decision-Making in the Management of Complex Urinary Tract Infections

**DOI:** 10.3390/ijms25126616

**Published:** 2024-06-16

**Authors:** Julia Elia, Jason Hafron, Mara Holton, Connor Ervin, Mitchell B. Hollander, Deepak A. Kapoor

**Affiliations:** 1 Michigan Institute of Urology, St. Clair Shores, MI 48081, USAhafronj@michiganurology.com (J.H.); hollanderm@michiganurology.com (M.B.H.); 2 Solaris Health Holdings, Ft. Lauderdale, FL 33394, USA; mholton@aaurology.com; 3 Anne Arundel Urology, Annapolis, MD 21401, USA; 4 PRISM Vision Group, Baltimore, MD 21201, USA; 5 Integrated Medical Professionals, Lake Success, NY 11042, USA

**Keywords:** clinical utility, urinary tract infections, urine culture, polymerase chain reaction, urinary tract infections management

## Abstract

While urinary polymerase chain reaction (PCR) testing is effective in organism identification in patients with complex urinary tract infections (cUTI), limited data exists on the clinical usefulness of this test. We serially surveyed physicians treating symptomatic patients with cUTI both at presentation and after PCR, and urine culture (UC) results were available to ascertain how the test results modified the therapy. A total of 96 unique surveys completed by 21 providers were included in the data analysis. The mean age for female and male patients was 69.4 ± 15.5 and 71.6 ± 12.7 years, respectively. The test positivity and line–item concordance for UC and PCR were consistent with prior reports. The PCR results modified or confirmed treatment in 59/96 (61.5%) and 25/96 (26.0%) of the cases, respectively, with 12/29 (41.4%) and 47/67 (70.1%) having negative and positive PCR results, respectively, resulting in treatment change (difference 28.7%, *p* < 0.01). Of these, 55/59 (57.3%) were alterations in the antibiotic regimen. PCR use to modify treatment was similar across providers and not statistically different when stratified by patient age, gender, or prior empiric therapy. In 31/59 (52.5%) of the cases, the PCR results modified the treatment where UC would not; conversely, UC would have modified the treatment in 3/37 (8.1%) of the cases where PCR did not (difference 44.4%, *p* < 0.01). We find that PCR test results are used by clinicians in managing cUTI, and use of this test provides an opportunity to improve antibiotic stewardship in this difficult-to-treat subset of patients.

## 1. Introduction

Urinary tract infections (UTI) are a leading cause of patient visits to healthcare providers, accounting for over 150 million cases worldwide annually [1]. While simple UTIs rapidly resolve with treatment, a subset of patients present with or develop complex UTIs (cUTIs), which are more likely to be associated with adverse outcomes, including a protracted therapeutic course, increased morbidity, and even mortality. In 2018, reports indicated that there were 626,000 hospital admissions for cUTI in the United States alone—this represents a six-fold increase over the prior decade and resulted in billions of dollars of system costs [2]. Because of the potential for serious and long-term complications and adverse effects on the GU tract or other organ systems in cUTIs, it is crucial that pathogen identification be both rapid and correct [3].

Traditionally, UTIs are diagnosed using a urine culture (UC). UC testing is based on collecting a urine sample, which is inoculated onto a culture plate and then incubated to allow colony growth to identify organisms in the sample [4,5]. However, for both uncomplicated cystitis and pyelonephritis, it has been shown that UCs exhibit negative results in patients with active UTIs [6]. A recent study of over 36,000 patients with cUTIs showed that real-time, semi-quantitative urinary polymerase chain reaction (PCR) testing using a panel of at least 25 organisms was superior to conventional UC in the detection of single and multiple organisms (including organisms considered fastidious) in cUTIs [7]. However, enhanced organism detection does not immediately imply that physicians will use these data, and that study did not assess whether the information gleaned from the PCR tests directly impacted clinical decision-making.

Molecular testing has achieved widespread use over the last four decades and is now considered an indispensable tool in the practice of clinical microbiology [8]. With respect to cUTIs, recent literature suggests that the use of empiric antibiotic therapy can be reduced, and outcomes improved [9] using urinary PCR testing. In addition, a recent report suggested that patients who had PCR testing had significantly lower episode cost of care when compared to those that received UC testing alone [10]. However, even those who acknowledge that such tests have the potential to enhance patient care and even decrease mortality raise concerns that the use of these tests potentially results in unnecessary treatment, thereby increasing antimicrobial resistance [11]. The purpose of this analysis was to evaluate the impact of PCR urine testing on clinical decision-making both overall and by provider in this difficult-to-treat subset of patients, and whether this was influenced by patient demographic factors, provider preference, organism type, or whether the patient received empiric therapy.

## 2. Results

### 2.1. Demographic Data

Of the initial 100 provider surveys, 4 were performed on duplicate patients. For data symmetry, only the initial encounter was included in the analysis. Complete demographic data stratified by PCR result, gender, age, and whether empiric therapy was initiated is presented in Supplement SA. In brief, of the 96 unique patients analyzed, the distribution of both gender and age was balanced. In total, there were 53/96 (55.2%) females and 43/96 (44.8%) males included in the analysis. The overall mean patient age was 70.4 ± 14.3 years, with mean age for females and males of 69.4 ± 15.5 years and 71.6 ± 12.7 years, respectively. In total, 21 providers contributed an average of 4.8 ± 4.0 surveys each to the analysis. All the providers contributing to the survey were physicians who were board-certified in urological surgery.

### 2.2. PCR and UC Rates

Table 1 compares the overall positivity rates of the PCR and UC tests. The PCR tests were positive in 67/96 (69.8%) of the patients compared to 46/96 (47.9%) of the patients for UC (difference 21.9%, *p* < 0.01). The PCR results were negative in 1/46 positive UCs (2.2%) while in 22/67 (32.8%) of positive PCR tests, UCs were negative (difference 30.6%, *p* < 0.01). There was a total of 103 instances of 18 unique organisms identified in the 67 positive PCR tests, while there were 46 instances of 10 unique organisms isolated in the UC. Of the positive PCR results, 23/67 (34.3%) of these tests showed polymicrobial UTI, with 15/23 (65.2%), 4/23 (17.4%), 3/23 (13.0%), and 1/23 (4.3%) revealing two, three, four, and five organisms, respectively. In addition, a total of 21/67 (31.3%) of the positive PCR results revealed fastidious organisms. No UC grew fastidious organisms or showed polymicrobial growth.

### 2.3. Line–Item Concordance Analysis

The line–item concordance for organisms between PCR and UC is depicted in Table 2. A total of 105 instances of 20 unique organisms were identified between both tests. The PCR tests identified 103/105 (98.1%) instances of 18/20 (90.0%) total organisms identified, while the UC tests identified 46/105 (43.8%) instances of 10/20 (50.0%) total organisms identified (instance differential 57.1%, *p* < 0.01, total organism difference 40.0%, *p* < 0.01). A total of 43/105 (41.0%) instances of 8/20 (40.0%) total organisms were found concurrently for both tests. The PCR showed high concordance with the UC in the positive cultures, being concordant in 43/46 (93.5%) of the organisms found in the UC tests. In contrast, the UC was concordant with the PCR results for 44/103 (42.7%) of the organisms identified using PCR (difference 51.7%, *p* < 0.01). Two of the three instances in which the PCR was discordant with the UC were where the UC grew organisms that were not present on the PCR panel, while 21/60 (35.0%) of the organisms found using PCR and not isolated in UC were fastidious.

### 2.4. Empiric Therapy Guided by PCR and UC Results

Most of the patients (55/96, 57.3%) in this analysis were started on empiric therapy. The average age of the patients who received empiric therapy was 72.3 ± 13.1 years vs. 67.7 ± 15.6 years for those who did not receive empiric therapy (difference 4.6 years, *p* = 0.13). A total of 30/43 (69.8%) of the male patients and 25/53 (47.2%) of the female patients received empiric therapy (difference 22.6%, *p* = 0.03). Table 3 illustrates the number of patients who did and did not receive empiric therapy based on the test and test results. For patients with positive UC results, 26/46 (56.5%) and 20/46 (43.5%) did and did not receive empiric therapy, respectively. For patients with positive PCR results, 37/67 (55.2%) and 30/67 (44.8%) did and did not receive empiric therapy, respectively. The differences in test positivity between the patients who did and did not receive empiric therapy were not significant: 13.0% for UC and 10.4% for PCR (*p* = 0.52 and 0.65, respectively). In addition, the test positivity in the patients started on empiric therapy between UC (26/46, 56.5%) and PCR (37/67, 55.2%) was similar (difference 1.3%, *p* = 0.89). The test results contrary to empiric therapy (positive test/no empiric therapy or negative test/with empiric therapy) were also similar between the tests, occurring in 49/96 (51.0%) of UC and 48/96 (50.0%) of PCR tests (difference 1.0%, *p* = 0.89).

### 2.5. Comparison of Treatment Change Based on PCR and UC Results

The treatment change based on results of PCR and UC testing is illustrated in Table 4. We found that in 34/96 (35.4%) and 28/96 (29.2%) of total cases, both UC and PCR would have either not altered or both altered clinical treatment, respectively (difference 6.2%, *p* = 0.35). In 31/96 (32.3%) of the cases, the PCR results altered the treatment when UC would not; conversely, UC would have altered the treatment where the PCR results would not in 3/96 (3.1%) cases (difference 29.2%, *p* < 0.01).

### 2.6. Comparison of Change in Treatment Using PCR by Age, Gender, and Provider

#### 2.6.1. Change in Treatment by PCR Results and Age

Table 5 presents the treatment changes, stratified by age and PCR results. The average age of the patients whose treatment was modified based on the PCR results was 71.2 ± 15.1 years, vs. 69.1 ± 13.0 years for patients whose treatment was not modified based on the PCR results (difference 2.1 years, *p* = 0.48). The average age of the 47 patients whose treatment was modified due to positive PCR findings was 73.0 ± 13.2 years versus 64.0 ± 19.5 years for the 12 patients whose treatment was modified based on negative PCR results (difference 9.0 years, *p* = 0.17). The average age of the 20 patients whose treatment was not modified based on the PCR findings was 71.4 ± 9.0 years versus 66.4 ± 16.1 years for the patients whose treatment was not modified by negative PCR results (difference 5.0 years, *p* = 0.28). In addition, the differences in patient age were not significant: 1.6 years for patients with positive PCR results whose treatment was or was not modified by PCR (*p* = 0.57) and 2.4 years for patients with negative PCR results whose treatment was or was not modified by PCR (*p* = 0.72).

#### 2.6.2. Change in Treatment by Gender

The impact of gender on the clinical use of the PCR result is presented in Table 6. In total, 32/53 (60.4%) of female patients and 27/43 (62.8%) of males had their treatment modified based on their PCR results (difference 2.4%, *p* = 0.84). Of the 59 total patients who had their treatment modified by the PCR results, 28/59 (47.4%) and 19/27 (32.2%) of female and male patients, respectively, had treatment modifications based on positive PCR results (difference 15.2%, *p* = 0.12). In total, 28/41 (68.3%) of females and 19/26 (73.1%) males with positive PCR results had treatment modifications based on these results (difference: 4.8%, *p* = 0.67).

#### 2.6.3. Use of PCR by Provider to Change Treatment 

**Figure 1:**
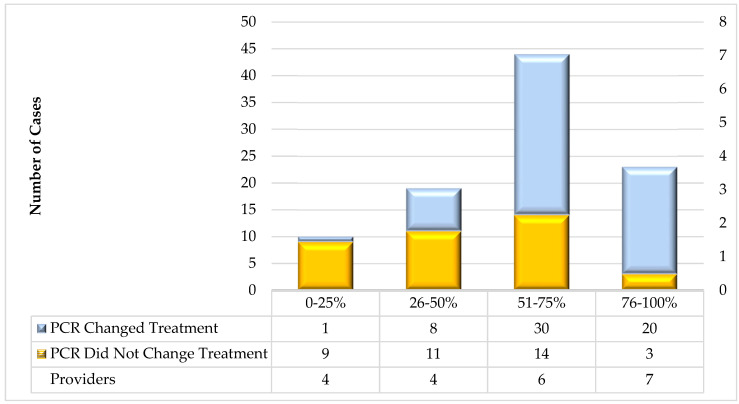
Use of PCR to Change Treatment by Provider.

In total, 21 providers contributed a median of 4 (range: 1–19) survey responses each. When averaged by provider, the PCR results modified treatment decision making in 60.5% ± 33.3% of cases (median 66.7%, range 0.0–100%). For analysis, the providers were grouped by quartiles based on the percentage of the times that the PCR results were used to modify treatment. The total number of providers grouped by quartile with the number of cases where the PCR results modified the treatment, as well as the average use of PCR to modify treatments, are presented in Figure 1. A total of 4, 4, 6, and 7 providers were in the 1st through 4th quartiles of PCR use, respectively. On average by quartile, the providers used PCR to modify treatments in 1/10 (10.0%), 8/19 (42.1%), 30/44 (68.2%), and 20/23 (87.0%) of total cases in the 1st through 4th quartile, respectively. Three providers did not use the PCR results to modify the treatments in any of their five cases, while five providers used the PCR results to modify the treatment in all seven of their cases. More than half of the providers (13/21, 61.9%) used the PCR results to modify their treatment in over 50% of cases, comprising 67/96 (69.8%) of the total cases, compared to 8/21 (38.1%) of the providers using PCR in less than 50% of cases, comprising 29/67 (30.2%) of the total cases. The 13 providers that used PCR more than half the time used the PCR results to modify the treatment in 50/67 (74.6%) of cases; those providers that used PCR to modify the treatment less than half the time used the test in 9/29 (31.0%) of instances.

### 2.7. Treatment Change in Patients with and without Empiric Therapy by PCR Result

The specifics of the use of both positive and negative PCR tests in patients who either did or did not receive empiric therapy are illustrated in Table 7. We found that 59/96 (61.5%) of the patients had modifications of their treatment based on PCR testing. Of these, 55/59 (93.2%) involved alterations in antibiotic regimen, while 4/59 (6.8%) resulted in the formation of an alternative plan of care (e.g., referral to another provider, initiation of alternative medication regimen). In total, 12/29 (41.4%) of the negative and 47/67 (70.1%) of the positive PCR results resulted in treatment change (difference 28.7%, *p* < 0.01). We found that 31/55 (56.4%) and 28/41 (68.3%) of the patients without and with empiric therapy, respectively, had changes in antibiotic treatment based on the PCR test results (difference: 11.9%, *p* = 0.23). Of the 41 patients who did not receive empiric therapy, 2/11 (18.2%) and 27/30 (86.7%) of the patients with negative and positive PCR, respectively, had their treatment modified based on the PCR results (difference 68.5%, *p* < 0.01). Lastly, of the patients who received empiric antibiotic therapy, 10/18 (55.6%) and 21/37 (56.8%) of the patients with negative and positive PCR, respectively, had their treatment modified based on the PCR results (difference: 1.2%, *p* = 0.94).

### 2.8. Comparison of Treatment Change by Organism

The specific treatment change by organism is presented in Supplement SA. A summary of these results grouped by organism type (fastidious, fungi, Gram-negative rods (GNR), and Gram-positive cocci (GPC)) is presented in Table 8. The number of organisms detected using PCR (103) exceeded the number of positive PCR specimens (67) due to the presence of multiple organisms in 23 specimens. The treatment change per patient was recorded for each organism identified in each case (for example, if a hypothetical sample revealed both *E. coli* and *A. urinae* and the clinician modified the therapy by changing antibiotics, this would be reflected in the result reported for both organism groups). We found that, in general, there was no statistically significant difference between the probability of any treatment change occurring for any category of organism when compared to the incidence of that organism group, with certain exceptions. Changes in antibiotic therapy were more likely to occur in fastidious organisms compared to the incidence of fastidious organisms (7/15, 46.7% vs. 21/103, 20.4%, respectively, difference: 26.3%, *p* = 0.03). Conversely, changes in antibiotic therapy (2/15, 13.3%) were less likely than the incidence of an organism group in GNR (48/103, 46.6%); the difference of 33.3% was significant (*p* = 0.01). Fungi resulted in a significant percentage, albeit small absolute, change in likelihood of the providers initiating an alternative plan of care (1/2, 50%) when compared to the incidence of fungal infections (4/103, 3.9%, difference 46.1%, *p* < 0.01). The only organism group in which it was more likely that the antibiotic treatment was extended when compared to the organism incidence was with GNR, which comprised 7/8 (87.5%) of this treatment change compared to an incidence of 48/103 (46.6%) for this organism type overall (difference 40.9%, *p* = 0.03). We noted that 5/6 (83.3%) of the cases where antibiotics were discontinued were with GPC, which was significantly higher than the incidence of 30/103 (29.3%) of the cases where this organism was detected (difference 54.2%, *p* = 0.01).

## 3. Discussion

Evaluating the usefulness of a diagnostic test depends on several factors. Together, these are defined as clinical utility, but unfortunately, the precise definition of clinical utility is vague [12]. While any new diagnostic test needs to show benefits over those already in use, this usefulness goes beyond merely being diagnostically accurate. In their mini-review, Miller et. al. suggested, with respect to advanced microbiology testing tools, that a test has utility if the test results provide information that is of decision-making value to patients and suggested the definition include not only outcomes but clinical workflow and/or cost offsets or avoidance [13]. Indeed, it has been recognized that specifically with regards to molecular diagnostic tests, clinical outcomes may be difficult to capture, but that outcome changes are predicated on changes in clinical behavior, and that changes in clinical practice are a more practical and feasible method to ascertain clinical utility [14]. To further understanding in this regard, we sought to study the impact of PCR urine testing on clinician decision-making for patients with cUTI seen in a single urology practice.

The purpose of this analysis was not to compare the diagnostic accuracy of PCR and UC, nor to assess PCR use in circumstances other than complex UTIs. However, to ensure that our results were consistent with prior reports, we evaluated the overall test positivity and individual organism line–item concordance for this subset of data. We found that our findings regarding the overall test sensitivity and line–item concordance were consistent with data previously published by the national reference laboratory that performed the PCR tests in this study [7], as well as in our subset analysis using this test in our practice [15]. In addition, although also not a focus of this study, we noted that even in this relatively limited number of patients, 18 distinct organisms were identified using PCR, consistent with reports that urinary molecular testing ideally is comprised of a comprehensive organism panel [7]. However, our purpose was not to revalidate urinary molecular testing but rather to determine how the results of PCR testing were used in a clinical setting.

Empiric therapy-prescribing methods are driven by the urgency to alleviate UTI symptoms and prevent future complications that are present in patients. This is particularly true in patients with cUTI, who have a higher likelihood of developing complications, including increased risk of hospitalization and even mortality. Though crucial for the immediate management of cUTI, empiric treatment comes with risks, including the overuse of broad-spectrum antibiotics or inappropriate antibiotic prescription [4,16,17,18]. These risks are well documented in this study: regardless of the test used, we found that initiation of empiric therapy was akin to a coin flip—approximately 50% of the patients who received or did not receive empiric antibiotics had that decision confirmed by laboratory testing. We did note that a greater number and percentage of male patients received empiric therapy when compared to their female counterparts. Although not a focus of this study, we ascertained that the differential was exclusively due to the patients presenting with signs or symptoms consistent with prostatitis, epididymitis, or orchitis, conditions not present in females.

Overall, we found that over 61% of the patients had their treatment modified based on the results of the PCR testing. We found that empiric antibiotic therapy commenced at the time of the initial patient encounter in well over half of the symptomatic patients who met the clinical criteria for cUTI. Importantly, whether the treatment was modified was not statistically different regardless of whether empiric therapy was instituted at the time of the initial visit. As would logically follow, we found that in those patients without empiric therapy, a positive PCR result was more likely to alter care than a negative test. However, positive and negative PCR tests were both likely to alter treatment in the patients who were started on empiric antibiotic therapy. Furthermore, the fact that the results of the PCR test did not specifically result in a modification of treatment does not imply that the clinicians did not incorporate the results of the test into their treatment plan. Of the 37 patients whose treatment was not modified by PCR, 16/37 (43.2%) had their empiric therapy confirmed by a positive PCR result, while an additional 9/37 (24.3%) had the decision not to initiate empiric therapy confirmed by a negative PCR result. Consequently, the true use of PCR by the clinicians can be observed in cases where treatment was either modified (59/96, 61.5%) or confirmed (25/96, 26.0%). In total, PCR provided relevant data for patient care in of 84/96 cases (87.5%) compared to 12/96 cases (12.5%) where the PCR results did not appear to be informative (difference: 75.0%, *p* < 0.01).

Importantly, the use of PCR to modify treatment decisions was broadly distributed across ordering providers. Even in circumstances where the PCR results did not modify the treatment, the test did aid the therapy, as exemplified by the five cases seen by the three providers who did not use the PCR results to modify the treatment in any cases. In this subgroup, in 4/5 (80%) cases, a positive PCR result confirmed the empiric treatment administered, or a negative PCR result confirmed that no antibiotic was needed where empiric treatment was not initiated. Furthermore, we found that neither age nor gender influenced whether PCR testing was used to modify treatment. Our findings suggest that PCR is a more influential diagnostic tool than UC in the context of cUTIs, especially in identifying pathogens that are not detected using traditional culture. This suggests that PCR can play a critical role in adjusting treatments, particularly in cases where a urine culture fails to identify the presence of pathogens, leading to more effective and targeted management of complex urinary tract infections.

We identified certain changes in treatment modifications based on the types of organisms detected using PCR. We found that the choice of antibiotic was more likely to be changed when a fastidious organism was found using PCR and less likely when a GNR was found. Indeed, the physicians continued or extended empiric antibiotic therapy in 21/25 (84.0%) of the cases where GNR were identified using PCR compared to 11/30 (36.7%) instances for fastidious organisms, fungi, and GPC combined (difference 47.3%, *p* < 0.01). This follows a clinical workflow reflecting the choice of empiric antibiotic prescribed or provided as samples when the patient was evaluated by the provider. We also noted that although GPC are increasingly recognized as urinary pathogens [19], careful clinical assessment is needed to differentiate whether this organism based on PCR results is indeed pathogenic—5/6 (83.3%) of cases where empiric antibiotics were stopped were when the PCR results detected GPC.

The overuse of broad-spectrum antibiotics can lead to adverse consequences such as allergies, *C. difficile* infections, microbiome disturbances, and drug resistance [5,20]. Antibiotic resistance is a significant worldwide issue caused by prolonged antibiotic use. Overusing broad-spectrum antibiotics contributes to antibiotic resistance, making UTIs increasingly challenging to treat and contributing to the high recurrence rates in complex UTIs [2,16,17,21]. It is essential to select the correct therapy for treatment to have optimal antibiotic stewardship. Proper antimicrobial stewardship is enhanced by test results being both timely and accurate—urinary molecular testing is both more rapid and can identify more potential pathogens than conventional UC. Of the 55 patients who received empiric antibiotic therapy, 24/55 (43.6%) had their therapy confirmed; however, 31/55 had their treatment modified based on the PCR findings. We observed that 14/31 (45.2%) of the patients had their antibiotics discontinued or had their diagnosis altered after PCR testing, vs. 17/31 (54.8%) who had their antibiotics either changed or the course extended. This difference of 9.6% was not significant (*p* = 0.45).

As the turnaround time for PCR is shorter than for UC, the question of whether UC would modify treatments is more difficult to ascertain. We queried providers as to whether the results of UC would have had an impact on clinical decision making independent of the PCR result. We found that in nearly 28/96 (29.2%) of cases, the clinicians reported that the UC and PCR results would have resulted in similar therapy modifications; however, we found that in 31/59 (52.5%) of cases where the PCR results modified the treatment, UC would not have done so. Conversely, UC would have modified treatment in 3/37 (8.1%) of the cases where PCR did not (difference 44.4%, *p* < 0.01). These results are due to the UC growth of organisms not included on the PCR panel. This aligns with prior reports [7,14] that suggest that rather than any one test being a gold standard, UC and PCR are complementary tests and are best used contemporaneously in patients with cUTI.

Although UC has long been considered the standard of care in the diagnosis of UTI, the existence of both false-negative and positive results has been known for some time. When considering whether a novel technology can supplant or augment existing historical standards, it is important to assess whether that test has clinical utility. Whether or not these patients with chronic illness showed improvement is also important, but the results of therapy are but one of the criteria that determine the value of performing a diagnostic test. A corollary to this can be seen in genomic testing for malignancy; in urology, it is estimated that approximately 14% of patients diagnosed with adenocarcinoma of the prostate have genetic predisposition to disease [22], yet consensus treatment guidelines are specific: regardless of the fact that actionable results are found in fewer than 1 in 5 patients, genetic testing should be offered to all patients with metastatic and certain newly diagnosed prostate cancers [23]. In our study, we found that nearly one-quarter (10/41) of the patients who did not receive empiric therapy would not have been treated had PCR testing not been performed, and over one-fifth (12/55) of those who received empiric treatment would have had their antibiotics needlessly continued.

Our analysis does have certain limitations. We relied on the clinician’s response to surveys and did not conduct a separate chart review; however, this limitation was mitigated by the fact these surveys were conducted contemporaneously with patient visits. In addition, as the patient selection was based on clinical pathways within a single practice specifically for cUTI using a specific PCR panel, the findings are potentially not relevant to groups that use different criteria for patient selection or an alternate PCR panel. However, the pathways developed in our practice resemble those published in prior reports and are consistent with a third-party review of the literature [7,9]. While we acknowledge that this study would be strengthened by a larger patient population, our survey number was sufficiently powered such that our findings achieved statistical significance.

## 4. Materials and Methods

### 4.1. Study Design

This was a prospective, open-label study completed at an independent urology group practice. The study’s primary objective was to measure the proportion of patients with cUTI for whom the results of the PCR urine test altered the planned treatment.

### 4.2. Patient Selection

Eligible patients included symptomatic patients older than 18 years old who presented to the urology office with a cUTI, as defined by our group’s clinical guidelines (presented in Supplement SA). The exclusion criteria included payor noncoverage for PCR testing. The data were collected during a 9-month period from November 2022 to 5 August 2023.

### 4.3. Study Protocol

The urologists were required to complete a two-part questionnaire administered before and after the results of the PCR test. The pre-PCR test questionnaire collected indications for testing, whether empiric therapy was instituted (including antibiotic choice and intended treatment duration), as well as basic patient demographic information. The patient demographic information was verified by the laboratory requisition for each patient. Once the results of the PCR testing were available, the survey respondents were asked to indicate if and how the PCR test revised patient management, including stopping, starting, changing, or modifying the duration of antibiotic therapy (typically, extensions of therapy occur when symptomatic patients are empirically provided samples pending urinary testing). In addition, the survey respondents were asked if the results of the PCR test prompted a change in diagnosis or treatment plan. The questionnaires were recorded in the patient’s electronic health record, reviewed, and entered into a database within two weeks of the visit. A schematic of the survey is presented in Supplement SA.

### 4.4. Specimen Collection, Urine Culture, and PCR Testing

#### 4.4.1. Specimen Collection

Each patient’s urine was collected through either midstream clean catch or via straight urinary catheter in a 4.0 mL gray-top BD Vacutainer^®^ Plus C&S Boric Acid Sodium Borate/Formate (BD Biosciences, Becton Lakes, NJ, USA) tubes specifically designed to transport urine with minimal sample degradation for 48 h when shipped at room temperature. All the urine specimens were shipped to P4 Diagnostix (Pine Brook, NJ, USA), a College of American Pathology (CAP), Clinical Laboratory Improvement Amendment (CLIA) accredited diagnostic laboratory via an overnight courier service. Once received, the urine specimens were first accessioned and then processed for UC and subsequently PCR.

#### 4.4.2. Urine Culture

One (1) µL of each patient’s urine sample was streaked with a standard disposable loop on each side of a biplate with TSA with 5% sheep blood and MacConkey agar plate (ThermoFisher Scientific, Carlsbad, CA, USA). The plates were incubated at 35–37 °C for at least 18 h, then visually inspected for colony growth and assessed for quantity and morphology. Cultures with no visible growth were further incubated for an additional 24 h and re-inspected. Isolated colonies were loaded onto a Vitek 2 (BioMerieux, Durham, NC, USA) overnight to enable microbial species-level identification and antimicrobial susceptibility testing according to the manufacturer’s instructions. The urine culture was considered positive if more than 5 × 10^4^ colony-forming units of any individual organisms were isolated.

#### 4.4.3. PCR Testing

##### Urine DNA Extraction

For DNA extraction from urine, an inverse magnetic particle processing (MPP) technology with multiple washes was used. The extraction protocol requires 800 µL of each patient’s urine to be aliquoted into each well of deep 96-well plates, sealed with foil, and then centrifuged. A total of 700 µL of supernatant was removed from each well. A total of 50 µL of enzyme mix for lysis was added to each well with concentrated urine and an extraction negative control (100 µL of nuclease-free water) and incubated at 65 °C for 20 min using a KingFisher Flex system (ThermoFisher Scientific). Subsequently, 240 µL of binding solution containing DNA beads, proteinase K (EO0491, ThermoFisher Scientific), and TaqMan Universal DNA Spike-In Control (A39175, ThermoFisher Scientific) was added into each well and then processed using the KingFisher Flex system for protein digestion and DNA extraction for 30 min.

##### PCR Analysis

Real-Time semi-quantitative PCR diagnostic testing was performed using the QuantStudio^TM^ 12K Flex Real-Time PCR system (ThermoFisher Scientific, Carlsbad, CA, USA). DNA isolated from the patient urine samples was placed in ThermoFisher OpenArray^TM^ plates using a ThermoFisher AccuFill^TM^ system. The OpenArray plates contained proprietary target primers and probes that were designed, optimized, in silico and wet lab validation tested, and manufactured by ThermoFisher Scientific. Real-Time PCR analysis was performed using the QuantStudio 12K Flex using the gene expression program according to the manufacturer’s proprietary OpenArray thermal cycling protocol and data analysis package (ThermoFisher Scientific, Carlsbad, CA, USA).

Regarding the PCR conditions, primers, probes, and amplification efficiency, 2.5 µL of extracted DNA from each patient’s urine sample and 2.5 µL of master mix (TaqMan OpenArray Real-Time PCR Master Mix, Applied BioSystems, ThermoFisher Scientific) were separately added into a 384-well plate and mixed well, then each sample was pipetted in duplicate onto the OpenArray plate and subsequently placed into the Applied Biosystems QuantStudio 12K Flex Real-Time PCR System (Applied BioSystems, ThermoFisher Scientific) for DNA amplification as instructed by the manufacturer. ThermoFisher Scientific provides MIQE-compliant documentation such as amplicon sequences available for human assays, but pursuant to their corporate policy, this information is considered proprietary for bacterial and antibiotic resistance targets [24]. TaqMan probes have been published in over 290,000 articles, and it is generally accepted to reference the assay ID numbers. The manufacturer will make available to the editor or any requestor the probe sequence, provided an NDA is in place with that party, on a case-by-case basis.

A positive run control, True Mark Comprehensive Microbiota Control (A50383, ThermoFisher Scientific), a negative run control (100 µL of nuclease-free water), and a negative extraction control were included in each OpenArray plate. The QuantStudio 12 Flex Software Program (Applied BioSystems, ThermoFisher Scientific) was used for PCR analysis, and the data were further analyzed for quality assurance and diagnostic test reporting.

To validate the molecular diagnostic assay, the limit of detection (LOD) was validated, as well as precision and inter- and intra-run reproducibility studies. LOD was performed to determine the ability of the assay to positively identify all the organisms on the panel at known concentrations. A positive PCR result (PCR+) was defined as any organism that displayed a cycle threshold above the threshold determined by the LOD studies using serial dilutions of the True Mark Comprehensive Microbiota Control through extinction. The LOD was compared extensively to the microbiology results to ensure the capture of a 10,000 or greater colony count. Limit of quantification (LOQ) studies were performed to determine the normal distribution of a population of randomly chosen samples at 2Σ. Positive targets were defined as those detected above the LOD threshold in duplicate, where: (a) the standard deviation was less than or equal to 2.0 with an amplification score of 1.24 or greater; (b) a minimum Cq confidence score of 0.8 was obtained (as recommended by ThermoFisher Scientific); and (c) the simultaneous presence of Taqman Universal DNA Spike-In Control (A39175, ThermoFisher Scientific) as a determinant of PCR inhibition was found. PCR efficiency was calculated using the geometric efficiency method rather than the standard curve efficiency method. We chose this method because it is the standard assessment tool by the manufacturer of the assay (ThermoFisher Scientific) [25], and it accounts for potential errors including PCR inhibitors, potential contamination, pipet precision, as well as calibration errors and mixing problems. This calculation resulted in a PCR efficiency range across the pathogen panel from a low of 90.6% (*E. aerogenes*) to a high of 103.6% (*C. parapsilosis*). Variability in the theoretical PCR efficiency does not imply variations in clinical sensitivity due to the LOD process described above, which created unique Crt cutoffs for each organism. An overview of the PCR conditions is provided in Supplement SA.

The precision and inter-run and intra-run reproducibility were evaluated based on the diagnostic performance utilizing synthetic, quantified genomic DNA obtained from ThermoFisher Scientific (True Mark Comprehensive Microbiota Control at 5 × 10^7^ copies/μL), urine samples from healthy patients, and urine samples from patients who were given a clinical diagnosis of a urinary tract infection. Precision and inter-run reproducibility were evaluated by demonstrating the presence of replicates within and across runs at multiple dilutions in triplicate.

Regarding the assay controls and quality control, the controls per run included a no template control (NTC), a positive template control (PTC), and a negative extraction control (NEC), in addition to the Taqman Universal DNA Spike-In Control, which served as the internal PCR inhibition control for each patient sample. The negative extraction control was processed alongside all clinical samples in each run, was processed in the same manner, and was used to monitor contamination during extraction or improper extraction setup. The negative template control was used to monitor contamination of reagents and improper PCR setup. A Taqman Universal DNA Spike-In Control was used in lieu of an endogenous control such as human gDNA due to its ability to function as an unbiased process control. Identification of PCR inhibition was monitored using this spike-in control. If there was a greater than 3 relative cycle thresholds (Crt) absolute difference between the Taqman Universal DNA Spike-In Control-NEC (negative extraction control) and the Taqman Universal DNA Spike-In Control patient sample, this was defined as inhibition. If there was a 50% or more height difference in the end-point fluorescence between the Taqman Universal DNA Spike-In Control-NEC and the Taqman Universal DNA Spike-In Control patient sample, this was a second indicator of PCR inhibition. Lastly, OpenArray plate QC images were reviewed for black through holes or improper loading of the plate by the Accufill, both of which possibly indicate improper OpenArray plate sample loading.

For the amplification plots/analysis, the ThermoFisher QuantStudio 12K analysis software set a threshold limit of 1.24, which was similar to the traditional PCR amplification curve score of 2.0, where the base and exponential phases are captured but the plateau is cut off due to late amplification of the target, even though the target is present. This defined spectrum excluded linear plots as high background or unusual components and required that the sample was rerun if detected. Amplification plots for each organism are provided in Supplement SB.

##### Targeted Organisms

This custom assay evaluated a total of 45 organisms (Table 9). The definition of a fastidious organism was any organism that in the performing laboratory (P4 Diagnostix), would require specific nutrients and atmospheric conditions including temperature, oxygen, and carbon dioxide to grow on agar plates (highlighted in blue in Table 9). Although not a part of this analysis, 18 antibiotic resistance genes were also part of the UTI OpenArray panel. Supplement SC provides the antibiotic resistance gene symbol, gene name, ThermoFisher Assay ID, and references to the Comprehensive Antibiotic Resistance Database (CARD). The CARD database link, drug class or classes, and resistance mechanism are described for each. The drug class or classes and resistance mechanism data were derived directly from the CARD database (https://card.mcmaster.ca/, accessed on 21 March 2024).

### 4.5. Data Analysis

Test positivity was calculated based on the ratio of positive tests to the total number of tests performed. The presence of polymicrobial infections or fastidious organisms was similarly tallied. Line–item concordance of organism positivity was performed following the method of Hao et al. [7]. The individual survey responses were tallied and aggregated in Microsoft Excel. The organisms detected using PCR reported in Supplement SA were grouped into four general categories: (a) fastidious organisms; (b) fungi; (c) Gram-negative rods (GNR); (d) Gram-positive cocci. A comparative analysis of treatment change by organism category was performed by comparing the number of cases where a treatment change occurred for a particular organism group with the overall incidence of detection by that organism group.

Given the sample size, a 2 × 2 contingency analysis of specimen positivity, empiric or therapeutic therapy by test type, age, and gender was performed using Fisher’s exact test, with *p* values reported either below or adjacent to the contingency table. Individual organism line–item concordance was analyzed using a Student’s paired *t*-test, and the age differential was determined using Student’s pooled *t*-test. Treatment modifications based on gender were compared using Fischer’s exact test, while those based on age were analyzed using a Student’s pooled *t*-test. Differences in the use of PCR testing based on empiric therapy and organism group were determined using a two-proportion z-test. Statistical analysis was performed using GraphPad Prism version 10.2.1 (395) (GraphPad Software, San Diego, CA, USA) and Microsoft Excel (Microsoft^®^ Excel^®^ for Microsoft 365 MSO (Version 2307 Build 16.0.16626.20170) 64-bit).

## 5. Conclusions

Our study confirms the technical superiority of PCR over UC in overall organism detection as well as the identification of polymicrobial infections and fastidious organisms in symptomatic patients with clinical presentation of cUTI. We found that the clinician’s initial decision to initiate empiric therapy correlated poorly with either the UC or PCR results. We found that in this difficult-to-treat subset of patients, PCR was significantly more likely to result in modification of the treatment regimen compared to UC. Importantly, the decision to use the PCR test results occurred whether the PCR result was positive or negative, with most treatment changes in both circumstances being initiation, cessation, or modification of antibiotic use. The decision to use PCR testing was uniform across providers and was independent of age, gender, diagnosis at presentation, or whether the patient had empiric antibiotic therapy. Nearly one-third of the patients had modifications of their antibiotic regimen based on the PCR test results that would not have been found using UC alone. We find that PCR testing is a valuable tool in the management of cUTI and has utility in improving antibiotic stewardship in this difficult-to-treat subset of patients.

## Figures and Tables

**Table 1:** PCR and UC Results.

Urine Culture	PCR (% Total Cases)		Total (%)
	Positive	Negative	
Positive	45 (46.9%)	1 (1.0%)	46 (47.9%)
Negative	22 (22.9%)	28 (29.2%)	50 (52.1%)
Total (%)	67 (69.8%)	29 (30.2%)	96 (100.0%)

*p* < 0.01.

**Table 2:** Line–item organism concordance for UC and PCR.

Organism	PCR+	UC+	Both+	UC Ratio	PCR Ratio	*p*
*E. coli*	35	26	25	74.3%	96.2%	0.02
*E. faecalis*	15	6	6	40.0%	100.0%	0.01
*A. urinae* *	13	0	0	0.0%	n/a	n/a
*U. urealyticum* *	7	0	0	0.0%	n/a	n/a
*K. pneumoniae*	6	3	3	50.0%	100.0%	0.13
*S. haemolyticus*	5	0	0	0.0%	n/a	n/a
*S. epidermidis*	3	2	2	66.7%	100.0%	0.36
*P. aeruginosa*	3	2	2	66.7%	100.0%	0.36
*E. cloacae*	3	2	2	66.7%	100.0%	0.36
*S. aureus*	2	2	2	100.0%	100.0%	1
*C. glabrata*	2	0	0	0.0%	n/a	n/a
*S. lugdunensis*	2	0	0	0.0%	n/a	n/a
*S. agalactiae*	2	1	1	50.0%	100.0%	0.38
*C. albicans*	1	0	0	0.0%	n/a	n/a
*S. pasteuranus*	1	0	0	0.0%	n/a	n/a
*C. koseri*	1	0	0	0.0%	n/a	n/a
*P. mirabilis*	1	0	0	0.0%	n/a	n/a
*M. hominis* *	1	0	0	0.0%	n/a	n/a
*C. braakii*	0	1	0	n/a	0.0%	n/a
*P. rettgeri*	0	1	0	n/a	0.0%	n/a
Total	103	46	43	42.7%	93.5%	<0.01

* Denotes fastidious organisms.

**Table 3:** Urine and PCR results in patients with and without empiric therapy.

Empiric Therapy	UC (% Total Cases)		Total (%)
	Positive	Negative	
Yes	26 (27.1%)	29 (30.2%)	55 (57.3%)
No	20 (20.8%)	21 (21.9%)	41 (42.7%)
Total (%)	46 (47.9%)	50 (52.1%)	96 (100.0%)
*p* = 0.52			
**Empiric Therapy**	**PCR (% Total Cases)**		**Total (%)**
	**Positive**	**Negative**	
Yes	37 (38.5%)	18 (18.8%)	55 (57.3%)
No	30 (31.3%)	11 (11.5%)	41 (42.7%)
Total (%)	67 (69.8%)	29 (30.2%)	96 (100.0%)

*p* = 0.65.

**Table 4:** Comparison of change in treatment based on UC vs. PCR.

Urine Culture	PCR (% Total Cases)		Total (%)
	Treatment Change	No Treatment Change	
Treatment change	28 (29.2%)	3 (3.1%)	46 (47.9%)
No treatment change	31 (32.3%)	34 (35.4%)	50 (52.1%)
Total (%)	59 (61.5%)	37 (38.5%)	96 (100.0%)

*p* < 0.01.

**Table 5:** Treatment change based on age and PCR results.

Treatment Change	N	Average Age (yrs.) *	*p*	PCR Results	N	Average Age (yrs.) *	*p*
Yes	59	71.2 ± 15.1	0.48	Positive	47	73.0 ± 13.2	0.17
				Negative	12	64.0 ± 19.5	
No	37	69.1 ± 13.0		Positive	20	71.4 ± 9.0	0.28
				Negative	17	66.4 ± 16.1	
Total	96	70.4 ± 14.3			96	70.4 ± 14.3	

* Total is of each 2 × 2 contingency table on which the *p* value is based.

**Table 6:** Treatment change by gender and PCR results.

Treatment Change	Gender		Total (%) *	*p*	PCR Results	Gender		Total (%) *	*p*
	F (%)	M (%)				F (%)	M (%)		
Yes	32 (33.3%)	27 (28.1%)	59 (61.5%)	0.84	Positive	28 (47.4%)	19 (32.2%)	47 (79.7%)	0.12
					Negative	4 (6.7%)	8 (13.5%)	12 (20.3%)	
					Total Yes	32 (54.2%)	27 (45.8%)	59 (100.0%)	
No	21 (21.9%)	16 (16.7%)	37 (38.5%)		Positive	13 (35.1%)	7 (18.9%)	20 (54.1%)	0.33
					Negative	8 (21.6%)	9 (24.3%)	17 (45.9%)	
					Total No	21 (56.8%)	16 (43.2%)	37 (100.0%)	
Total (%)	53 (55.2%)	43 (44.8%)	96 (100.0%)			53 (55.2%)	43 (44.8%)	96 (100.0%)	

* Total is of each 2 × 2 contingency table on which the *p* value is based.

**Table 7:** Treatment modifications based on PCR results in patients with and without empiric therapy.

Treatment Change		Empiric Therapy (% Total Cases)		No Empiric Therapy (% Total Cases)		Total
		Pos PCR	Neg PCR	Pos PCR	Neg PCR	
Modification of antibiotic therapy	Extended duration	6 (6.3%)				6 (6.3%)
	Changed antibiotic	11 (11.5%)				11 (11.5%)
	Started antibiotic			25 (26.0%)		25 (26.0%)
	Stopped antibiotic	3 (3.1%)	10 (10.4%)			13 (13.5%)
All Modifications of antibiotic therapy		20 (20.8%)	10 (10.4%)	25 (26.0%)		55 (57.3%)
Other changes	Alternate care plan	1 (1.0%)		1 (1.0%)	2 (2.1%)	4 (4.2%)
Total changes in treatment		21 (21.9%)	10 (10.4%)	27 (27.1%)	2 (2.1%)	59 (61.5%)
No changes in treatment		16 (16.7%)	8 (8.3%)	4 (4.2%)	9 (9.4%)	37 (38.5%)
All patients total		37 (38.5%)	18 (18.8%)	30 (31.3%)	11 (11.5%)	96 (100.0%)

**Table 8:** Treatment change by organism category *.

All	Organism by Treatment Category								Total (%)
	Fastidious		Fungi		GNR		GPC		
	n (%)	*p*	n (%)	*p*	n (%)	*p*	n (%)	*p*	
No treatment change	5 (17.2%)	0.70	1 (3.4%)	0.91	16 (55.2%)	0.41	7 (24.1%)	0.60	29 (100.0%)
Changed antibiotic	7 (46.7%)	0.03	0 (0.0%)	0.44	2 (13.3%)	0.01	6 (40.0%)	0.40	15 (100.0%)
Extended duration	0 (0.0%)	0.16	0 (0.0%)	0.57	7 (87.5%)	0.03	1 (12.5%)	0.31	8 (100.0%)
Started antibiotic	9 (20.9%)	0.94	2 (4.7%)	0.83	21 (48.8%)	0.80	11 (25.6%)	0.67	43 (100.0%)
Stopped antibiotic	0 (0.0%)	0.22	0 (0.0%)	0.62	1 (16.7%)	0.15	5 (83.3%)	0.01	6 (100.0%)
Alternate plan of care	0 (0.0%)	0.48	1 (50.0%)	<0.01	1 (50.0%)	0.92	0 (0.0%)	0.37	2 (100.0%)
Total	21 (20.4%)		4 (3.9%)		48 (46.6%)		30 (29.1%)		103 (100.0%)

* *p* values are percent of organism group as total of each treatment change vs. ratio of organism group to total organisms.

**Table 9:** Forty-five organisms and one positive control in the PCR panel *.

Organism	Fisher’s Assay Idx	Organism	Fisher’s Assay Idx
*A. baumannii*	Ba04932084_s1	*S. oralis*	AP9HJTH
*C. albicans*	Fn04646233_s1	*C. glabrata*	Fn04646240_s1
*C. freundii*	Ba04932088_s1	*M. tuberculosis*	APEPTGE
*E. aerogenes*	Ba04932080_s1	*M. genitalium*	Ba04646251_s1
*E. cloacae*	Ba04932087_s1	*M. hominis*	Ba04646255_s1
*E. faecalis*	Ba04646247_s1	*P. agglomerans*	AP47WMK
*E. faecium*	Ba04932086_s1	*U. urealyticum*	Ba04646254_s1
*E. coli*	Ba04646242_s1	*CMV*	Pa03453400_s1
*K. oxytoca*	Ba04932079_s1	*HSV1*	Vi04230116_s1
*K. pneumoniae*	Ba04932083_s1	*HSV2*	Vi04646232_s1
*M. morganii*	Ba04932078_s1	*A. schaalii*	AIPAFMX
*P. mirabilis*	Ba04932076_s1	*A. urinae*	AIQJDS5
*P. stuartii*	Ba04932077_s1	*A. omnicolens*	AIVI6H1
*P. aeruginosa*	Ba04932081_s1	*C. parapsilosis*	Fn04646221_s1
*S. aureus*	Ba04646259_s1	*C. riegelii*	AI5IRVT
*S. saprophyticus*	Ba04932085_s1	*C. urealyticum*	AI39TPL
*S. agalactiae*	Ba04646276_s1	*C. trachomatis*	Ba04646249_s1
*C. koseri*	AIX02UH	*N. gonorrhoeae*	Ba04646252_s1
*S. epidermidis*	Ba04230918_s1	*T. vaginalis*	Pr04646256_s1
*S. lugdunenesis*	APTZ9W7	*S. pasteuranus*	APCE4P6
*S. haemolyticus*	APMFXMX	*S. pyogenes*	AIVI6AD
*S. marcescens*	AIMSIYA	*Human Herpesvirus 6*	AI1RW3J
*C. amazonitic*	AP7DR2J	*Xeno Assay Control*	Ac00010014_a1

* ThermoFisher Scientific performed an in-silico study of on and off targets for each analyte sequence on the panel; fastidious organisms are denoted in blue.

## Data Availability

The original data in this study are restricted from public access due to patient privacy.

## Supplementary Materials

The following supporting information can be downloaded at: https://www.mdpi.com/article/10.3390/ijms25126616/s1. References [24,26,27,28] are cited in the Supplementary Material.

## References

[1] Mancuso G., Midiri A., Gerace E., Marra M., Zummo S., Biondo C. (2023). Urinary tract infections: The current scenario and future prospects. Pathogens.

[2] Zilberberg M.D., Nathanson B.H., Sulham K., Shorr A.F. (2022). Descriptive epidemiology and outcomes of emergency department visits with complicated urinary tract infections in the United States, 2016–2018. J. Am. Coll Emerg. Physicians Open.

[3] Johansen T.E.B., Botto H., Cek M., Grabe M., Tenke P., Wagenlehner F.M., Naber K.G. (2011). Critical review of current definitions of urinary tract infections and proposal of an EAU/ESIU classification system. Int. J. Antimicrob. Agents.

[4] Medina M., Castillo-Pino E. (2019). An introduction to the epidemiology and burden of urinary tract infections. Ther. Adv. Urol..

[5] Patel R., Polage C.R., Dien Bard J., May L., Lee F.M., Fabre V., Hayden M.K., Doernberg S.D., Haake D.A., Trautner B.W. (2022). Envisioning Future Urinary Tract Infection Diagnostics. Clin. Infect. Dis..

[6] Price T.K., Dune T., Hilt E.E., Thomas-White K.J., Kliethermes S., Brincat C., Brubaker L., Wolfe A.J., Mueller E.R., Schreckenberger P.C. (2016). The Clinical Urine Culture: Enhanced Techniques Improve Detection of Clinically Relevant Microorganisms. J. Clin. Microbiol..

[7] Hao X., Cognetti M., Patel C., Jean-Charles N., Tumati A., Burch-Smith R., Holton M., Kapoor D.A. (2023). The Essential Role of PCR and PCR Panel Size in Comparison with Urine Culture in Identification of Polymicrobial and Fastidious Organisms in Patients with Complicated Urinary Tract Infections. Int. J. Mol. Sci..

[8] Schmitz J.E., Stratton C.W., Persing D.H., Tang Y.W. (2022). Forty years of molecular diagnostics for infectious diseases. J. Clin. Microbiol..

[9] Haley E., Luke N., Korman H., Baunoch D., Wang D., Zhao X., Mathur M. (2023). Improving Patient Outcomes While Reducing Empirical Treatment with Multiplex-Polymerase-Chain-Reaction/Pooled-Antibiotic-Susceptibility-Testing Assay for Complicated and Recurrent Urinary Tract Infections. Diagnostics.

[10] Ko D.S., Lukacz E.S., Juster I.A. (2023). Real-world evidence that a novel diagnostic combining molecular testing with pooled antibiotic susceptibility testing is associated with reduced infection severity and lower cost compared with standard urine culture in patients with complicated or persistently recurrent urinary tract infections. JU Open Plus.

[11] Medicare Local Coverage Determination L39044, MolDX: Molecular Syndromic Panels for Infectious Disease Pathogen Identification Testing, Effective date 4/17/2022. https://www.cms.gov/medicare-coverage-database/view/lcd.aspx?lcdid=390442/19/2024.

[12] Jackson L.M., Parker R.M., Mattison D.R. (2020). The Clinical Utility of Compounded Bioidentical Hormone Therapy: A Review of Safety, Effectiveness, and Use.

[13] Miller M.B., Atrzadeh F., Burnham C.A.D., Cavalieri S., Dunn J., Jones S., Mathews C., McNult P., Meduri J., Newhouse C. (2019). Clinical utility of advanced microbiology testing tools. J. Clin. Microbiol..

[14] Peabody J.W., Shimkhada R., Tong K.B., Zubiller M.B. (2014). New thinking on clinical utility: Hard lessons for molecular diagnostics. Am. J. Manag. Care.

[15] Kapoor D.A., Holton M.R., Hafron J., Aljundi R., Zwaans B., Hollander M. (2024). Comparison of Polymerase Chain Reaction and Urine Culture in the Evaluation of Patients with Complex Urinary Tract Infections. Biology.

[16] Bartoletti R., Cai T., Wagenlehner F.M., Naber K., Bjerklund Johansen T.E. (2016). Treatment of Urinary Tract Infections and Antibiotic Stewardship. Eur. Urol. Suppl..

[17] Abbo L.M., Hooton T.M. (2014). Antimicrobial Stewardship and Urinary Tract Infections. Antibiotics.

[18] Dryden M., Johnson A.P., Ashiru-Oredope D., Sharland M. (2011). Using antibiotics responsibly: Right drug, right time, right dose, right duration. J. Antimicrob. Chemother..

[19] Gajdács M., Ábrók M., Lázár A., Burián K. (2020). Increasing relevance of Gram-positive cocci in urinary tract infections: A 10-year analysis of their prevalence and resistance trends. Sci. Rep..

[20] Butler A.M., Durkin M.J., Keller M.R., Ma Y., Powderly W.G., Olsen M.A. (2021). Association of Adverse Events With Antibiotic Treatment for Urinary Tract Infection. Clin. Infect. Dis..

[21] Foxman B. (2014). Urinary Tract Infection Syndromes: Occurrence, Recurrence, Bacteriology, Risk Factors, and Disease Burden. Infect. Dis. Clin. N. Am..

[22] Gunn C.M., Li E.X., Gignac G.A., Pankowska M., Loo S., Zayhowski K., Wang C. (2023). Delivering Genetic Testing for Patients with Prostate Cancer: Moving Beyond Provider Knowledge as a Barrier to Care. Cancer Control.

[23] Schaeffer E.M., Srinivas S., Adra N., An Y., Barocas D., Bitting R., Bryce A., Chapin B., Cheng H.H., D’Amico A.V. (2023). Prostate Cancer, Version 4.2023, NCCN Clinical Practice Guidelines in Oncology. J. Natl. Compr. Cancer Netw. JNCCN.

[24] Publish Real-Time PCR Results with Confidence. https://www.thermofisher.com/us/en/home/life-science/pcr/real-time-pcr/real-time-pcr-assays/why-choose-taqman-assays/publish-real-time-pcr-results.html.

[25] Efficiency of Real-Time PCR. https://www.thermofisher.com/us/en/home/life-science/pcr/real-time-pcr/real-time-pcr-learning-center/real-time-pcr-basics/efficiency-real-time-pcr-qpcr.html.

[26] Lantz P.G., Matsson M., Wadström T., Rådström P. (1997). Removal of PCR inhibitors from human faecal samples through the use of an aqueous two-phase system for sample preparation prior to PCR. J. Microbiol. Meth..

[27] Al-Soud W.A., Rådström P. (2001). Purification and characterization of PCR inhibitory components in blood cells. J. Clin. Microbiol..

[28] Bickley J., Short J.K., McDowell D.G., Parkes H.C. (1996). Polymerase chain reaction (PCR) detection of Listeria monocytogenes in diluted milk and reversal of PCR inhibition caused by calcium ions. Lett. Appl. Microbiol..

