# Supplementary material for: The Impact of Polymerase Chain Reaction Urine Testing on Clinical Decision-Making in the Management of Complex Urinary Tract Infections
Source: Int J Mol Sci. 2024 Jun 16;25(12):6616. doi: 10.3390/ijms25126616 (PMC11203880; doi:10.3390/ijms25126616)
Supplement: Supplementary file 1 [file ijms-25-06616-s001.zip › ijms-2971201-supplementary.pdf]

The Impact of Polymerase Chain Reaction Urine Testing on Clinical Decision-Making in the Management of Complex Urinary Tract Infections

Supplement A

Julia Elia, Jason Hafron, Mara Holton, Connor Ervin, Mitchell B. Hollander, and Deepak A. Kapoor

Table of Contents

Table of Contents 1
Clinical Indications for PCR Testing 2
Provider Survey 2
Patient Demographic Information 3
Treatment Change by Organism, With and Without Empiric Antibiotic Therapy 4
Inhibition Considerations 5
Primer/Probe Sequences 5
List of Organisms on the UTI Open Array Panel 6
PCR Conditions 7
Control Methodology 7
PCR Efficiencies, 95% CI's and Amplification Plot 7
Limit of Detection (LOD) 8
PCR Values and Efficiency 9
List of Antibiotic Resistance Genes on UTI OpenArray Panel 10
Antibiotic Resistance Gene CARD Database Location 11
References 11

Clinical Indications for PCR Testing

Although the determination of clinical appropriateness of a diagnostic test is determined by the clinician's evaluation of each patient's unique needs, our group generally recommends that urinary PCR testing be performed on symptomatic patients who meet the following criteria:

- Recurrent UTI
  a. ≥3/year or 2 in ≤6 months
- Complicated UTI (any of the following)
  a. Male
  b. Symptoms >7 days
  c. Co-morbidities (systemic and GU)
  d. Diabetes mellitus
  e. Immunosuppression
  f. Neurogenic bladder
  g. GU structural or functional abnormality
  h. Urolithiasis
  i. Recent hospitalization
  j. Pyelonephritis

Provider Survey

The below is a schematic of the survey providers were asked to complete. Part I was to be completed at the time of the initial patient visit while Part II was to be completed after evaluation of PCR results.

1. Part I – To be completed at time of initial patient visit.
   a. Patient Data
      i. Last Name, First Name, date of birth
      ii. Medical Record Number
   b. Treatment at presentation
      i. Was empiric antibiotic therapy initiated at the time of the initial visit? (Yes or No)
      ii. If the answer to 2(a) was yes, please indicate:
      iii. The antibiotic prescribed; and
      iv. The planned duration of therapy.
2. Part II – To be completed after evaluation of PCR Results
   a. Was the management plan revised after PCR results? (Yes or No)
   b. If the answer to 3(a) was yes, please choose from the following:
      i. Extended duration of antibiotic therapy.
      ii. Changed antibiotic treatment regimen.
      iii. Initiated antibiotic therapy.
      iv. Stopped antibiotic therapy.
      v. Referred to alternate caregiver.
      vi. Other (please provide details)
      vii. Please provide any additional comments you feel are relevant.

Patient Demographic Information

PCR Result	Empiric Treatment	Female		Male		Total	
		N	Average Age	N	Average Age	N	Average Age
Positive	Y	20 (20.8%)	74.1	17 (17.7%)	75.4	37 (38.5%)	74.7
	N	21 (21.8%)	69.6	9 (9.3%)	70.6	30 (31.2%)	69.9
Positive PCR Total		41 (42.7%)	71.8	26 (27%)	73.7	67 (69.7%)	72.5
Negative	Y	5 (5.2%)	64.6	13 (13.5%)	68.7	18 (18.7%)	67.6
	N	7 (7.2%)	58.4	4 (4.1%)	67.8	11 (11.4%)	61.8
Negative PCR Total		12 (12.5%)	61.0	17 (17.7%)	68.5	29 (30.2%)	65.4
Grand Total		53 (55.2%)	69.4	43 (44.7%)	71.6	96 (100%)	70.4

Treatment Change by Organism, With and Without Empiric Antibiotic Therapy

Listed are treatment changes by type and organism, for cases with and without empiric therapy for patients with positive PCR results.

Empiric Therapy	Treatment Change	Fastidious			Fungi			GNR					GPC							Total
		A. urinae	*M. hominis*	*U. urealyticum*	*C. albicans*	*C. glabrata*	*C. koseri*	*E. cloacae*	*E. coli*	*K. pneumoniae*	*P. aeruginosa*	*P. mirabilis*	*E. faecalis*	*S. agalactiae*	*S. aureus*	*S. epidermidis*	*S. haemolyticus*	*S. Lugdunensis*	*S. pasteuranus*	
Yes	No Change	4 (16%)	(0%)	1 (4%)	(0%)	(0%)	(0%)	2 (8%)	9 (36%)	3 (12%)	(0%)	(0%)	3 (12%)	2 (8%)	(0%)	(0%)	1 (4%)	(0%)	(0%)	25 (100%)
	Changed antibiotic	4 (26.7%)	1 (6.7%)	2 (13.3%)	(0%)	(0%)	(0%)	(0%)	2 (13.3%)	(0%)	(0%)	(0%)	4 (26.7%)	(0%)	1 (6.7%)	1 (6.7%)	(0%)	(0%)	(0%)	15 (100%)
	Extended duration	(0%)	(0%)	(0%)	(0%)	(0%)	(0%)	(0%)	6 (75%)	1 (12.5%)	(0%)	(0%)	1 (12.5%)	(0%)	(0%)	(0%)	(0%)	(0%)	(0%)	8 (100%)
	Stopped antibiotic	(0%)	(0%)	(0%)	(0%)	(0%)	(0%)	(0%)	1 (16.7%)	(0%)	(0%)	(0%)	1 (16.7%)	(0%)	(0%)	1 (16.7%)	2 (33.3%)	1 (16.7%)	(0%)	6 (100%)
	Alternate Plan of Care	(0%)	(0%)	(0%)	(0%)	(0%)	(0%)	(0%)	(0%)	1 (100%)	(0%)	(0%)	(0%)	(0%)	(0%)	(0%)	(0%)	(0%)	(0%)	1 (100%)
Empiric Therapy Total		8 (14.5%)	1 (1.8%)	3 (5.5%)	(0%)	(0%)	(0%)	2 (3.6%)	18 (32.7%)	5 (9.1%)	(0%)	(0%)	9 (16.4%)	2 (3.6%)	1 (1.8%)	2 (3.6%)	3 (5.5%)	1 (1.8%)	(0%)	55 (100%)
No	No Change	(0%)	(0%)	(0%)	(0%)	(0%)	1 (25%)	(0%)	2 (50%)	(0%)	(0%)	(0%)	(0%)	(0%)	(0%)	(0%)	1 (25%)	(0%)	(0%)	4 (100%)
	Started antibiotic	5 (11.6%)	(0%)	4 (9.3%)	1 (2.3%)	1 (2.3%)	(0%)	1 (2.3%)	15 (34.9%)	1 (2.3%)	3 (7%)	1 (2.3%)	6 (14%)	(0%)	1 (2.3%)	1 (2.3%)	1 (2.3%)	1 (2.3%)	1 (2.3%)	43 (100%)
	Alternate Plan of Care	(0%)	(0%)	(0%)	(0%)	1 (100%)	(0%)	(0%)	(0%)	(0%)	(0%)	(0%)	(0%)	(0%)	(0%)	(0%)	(0%)	(0%)	(0%)	1 (100%)
No Empiric Therapy Total		5 (10.4%)	(0%)	4 (8.3%)	1 (2.1%)	2 (4.2%)	1 (2.1%)	1 (2.1%)	17 (35.4%)	1 (2.1%)	3 (6.3%)	1 (2.1%)	6 (12.5%)	(0%)	1 (2.1%)	1 (2.1%)	2 (4.2%)	1 (2.1%)	1 (2.1%)	48 (100%)
Grand Total		13 (12.6%)	1 (1%)	7 (6.8%)	1 (1%)	2 (1.9%)	1 (1%)	3 (2.9%)	35 (34%)	6 (5.8%)	3 (2.9%)	1 (1%)	15 (14.6%)	2 (1.9%)	2 (1.9%)	3 (2.9%)	5 (4.9%)	2 (1.9%)	1 (1%)	103 (100%)

Inhibition Considerations

To exclude the potential PCR inhibitors including bile salts [1], hemoglobin [2] and proteinase [3] in urine specimens, an inverse magnetic particle processing (MPP) technology with multiple washes, an ideal method for automation of high-throughput DNA extraction, was used.

TaqMan Universal DNA Spike-In Control (SIC) serves as an exogenous process control of a known concentration that is spiked into a sample at specific stages throughout the qPCR process. Measuring the concentration of the spiked-in sequence allows for its use as a serial quality control and it can be used to monitor recovery efficiency and to identify false negative results such as the presence of PCR inhibitors in molecular detection workflows. This is of particular importance when working with sample types that have a high level of inhibition. For a 1x version of this control, please see Cat. No. A39175.

Spike-In Control is serially diluted to obtain its cut-off threshold (or LOD). Inhibition is monitored using this spike-in control. If the relative cycle threshold (C_{rt}) shows a greater than 3 C_{rt} absolute difference between SIC-NEC (the negative extraction control) and SIC-clinical sample, this is defined as inhibition. Also, if there is a 50% or more height difference in the end-point fluorescence between SIC-NEC and SIC-clinical sample, this too is an indicator of inhibition. Lastly, OpenArray™ plate QC images are reviewed for black through-holes or improper loading of the plate by the Accufill, both of which may indicate improper OpenArray plate sample loading.

Primer/Probe Sequences

In silico design of primer and probe sets, on target and off target in silico studies, and wet lab sensitivity and specificity studies were performed by ThermoFisher Scientific. P4 Diagnostix, a CLIA licensed, CAP accredited diagnostic laboratory completed clinical diagnostic validation and obtained regulatory approval from the Department of Health of the State of New Jersey to perform the diagnostic test. ThermoFisher Scientific makes MIQE-compliant documentation available such as amplicon sequence available in human assays but pursuant to their corporate policy, this information is considered proprietary for bacterial and antibiotic resistance targets. [4] TaqMan probes have been published in over 290,000 articles and general acceptance is to reference the assay ID #'s. The manufacturer will make available to the editor or any requestor the probe sequence provided an NDA is in place with that party on a case-by-case basis.

List of Organisms on the UTI Open Array Panel

UTI Organisms	ThermoFisher Probeset ID	UTI Organisms	ThermoFisher Probeset ID	ThermoFisher ABR Assay	ThermoFisher Probeset ID
A. baumannii	Ba04932084_s1	*S. oralis*	AP9HJTH	*VanA*	Ba04646147_s1
C. albicans	Fn04646233_s1	*C. glabrata*	Fn04646240_s1	*β-lactamase (class D)*	Ba04646133_s1
C. freundii	Ba04932088_s1	*M. tuberculosis*	APEPTGE	*AmpC β-lactamase (Class C)*	Ba04646126_s1
E. aerogenes	Ba04932080_s1	*M. genitalium*	Ba04646251_s1	*Carbapenemase (Class A)*	Ba04646151_s1
E. cloacae	Ba04932087_s1	*M. hominis*	Ba04646255_s1	*Carbapenemase (Class A)*	Ba04646152_s1
E. faecalis	Ba04646247_s1	*P. agglomerans*	AP47WMK	*Carbapenemase (Class B)*	Ba04646131_s1
E. faecium	Ba04932086_s1	*U. urealyticum*	Ba04646254_s1	*Carbapenemase (Class B)*	Ba04931076_s1
E. coli	Ba04646242_s1	*CMV*	Pa03453400_s1	*Carbapenemase (Class D)*	Ba04930816_s1
K. oxytoca	Ba04932079_s1	*HSV1*	Vi04230116_s1	*Extended Spectrum β-lactamase (Class A)*	Ba04646140_s1
K. pneumoniae	Ba04932083_s1	*HSV2*	Vi04646232_s1	*Extended Spectrum β-lactamase (Class A)*	Ba04646153_s1
M. morganii	Ba04932078_s1	*A. schaalii*	AIPAFMX	*Extended-Spectrum-β-Lactamase*	Ba04646149_s1
P. mirabilis	Ba04932076_s1	*A. urinae*	AIQJDS5	*Extended-Spectrum-β-Lactamase*	Ba04646142_s1
P. stuartii	Ba04932077_s1	*A. omnicolens*	AIVI6H1	*Extended-Spectrum-β-Lactamase*	Ba04646154_s1
P. aeruginosa	Ba04932081_s1	*C. parapsilosis*	Fn04646221_s1	*Extended-Spectrum-β-Lactamase*	Ba04646127_s1
S. aureus	Ba04646259_s1	*C. riegelii*	AI5IRVT	*Macrolide Resistance*	Pa04230913_s1
S. saprophyticus	Ba04932085_s1	*C. urealyticum*	AI39TPL	*Quinolone Resistance*	Ba04646160_s1
S. agalactiae	Ba04646276_s1	*C. trachomatis*	Ba04646249_s1	*Quinolone Resistance*	Ba04646145_s1
C. koseri	AIX02UH	*N. gonorrhoeae*	Ba04646252_s1	*verona integron-encoded metallo-β-lactamase*	Ba04646155_s1
S. epidermidis	Ba04230918_s1	*T. vaginalis*	Pr04646256_s1		
S. lugdunenesis	APTZ9W7	*S. pasteuranus*	APCE4P6		
S. haemolyticus	APMFXMX	*S. pyogenes*	AIVI6AD		
S. marcescens	AIMSIYA	*H. herpesvirus-6*	AI1RW3J		
C. amalonaticus	AP7DR2J	*H. herpesvirus-7*	Vi03453404_s1		
S. oralis	AP9HJTH	Xeno Control	Ac00010014_a1		

PCR Conditions

PCR Conditions for the OpenArray plate are encompassed in the software program within the ThermoFisher Quantstudio 12K system. The specific conditions are considered proprietary information by ThermoFisher Scientific for OpenArray technology.

Example of Amplification Plot of TrueMark Comprehensive Microbiota Control:

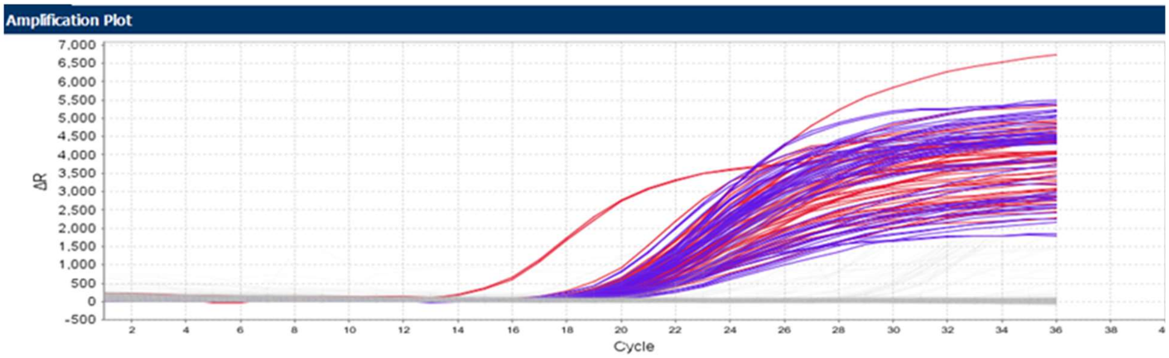

Amplification Plot: This is a 1/100 dilution of the TrueMark Comprehensive Microbiota Control. Each independent amplification curve represents either an organism or antibiotic resistance gene in the panel. All four classic features of a positive amplification result with the history or baseline, the exponential phase, the linear phase, and the plateau are demonstrated.

Control Methodology

The controls per run include (a) negative template control (NTC), (b) positive template control (PTC), (c) and a negative extraction control (NEC), in addition to the SIC as an internal PCR inhibition control per sample. We have two negative controls, NEC (negative extraction control) and NTC (negative template control). The negative extraction control is processed alongside all clinical samples in each run and is treated similarly, used to monitor for contamination during extraction or improper extraction setup. The negative template control is used to monitor for contamination of reagents and improper PCR setup. The SIC is used in place of endogenous controls such as human gDNA due to its ability to act as an unbiased process control.

PCR Efficiencies, 95% CI's and Amplification Plot

Traditional PCR scores amplification curves (sigmoidal in presentation) by the presence of three components: base, exponential, and plateau. Given that all three are present and discernible, the highest score traditionally given is 3. ThermoFisher's analysis software sets a threshold limit to 1.24, which is similar to the traditional score of 2.0, where the base and exponential phases are captured; but the plateau is cut off due to late amplification of the target even though the target is present. This defined spectrum ultimately excludes linear plots as high background or unusual component and requires that the sample is rerun if observed.

A component of the analytical validation of the UTI OpenArray Panel was demonstration of serial dilutions of each organism performed in triplicate. Each organism has a logarithmic curve to C_{rt} value calculation, a standard dilution curve, as well as a PCR efficiency calculation across the range of PCR cycles. The details of the PCR efficiency calculation per target, by dilution, are provided in Supplement B.

An example of *E. coli* PCR Efficiency is illustrated below:

DNA copies/ 2.5 µl	log DNA copies/2.5 ul (x)	Relative Threshold (C_{rt}) Value (y)	PCR Efficiency
5,558,020	6.7449	15	95.67%
2,904,658	6.4631	16	95.67%
1,517,993	6.1813	17	95.67%
793,313	5.8994	18	95.67%
414,591	5.6176	19	95.67%
216,668	5.3358	20	95.67%
113,232	5.054	21	95.67%
59,176	4.7721	22	95.67%
30,926	4.4903	23	95.67%
16,162	4.2085	24	95.67%
8,446	3.9267	25	95.67%
4,414	3.6448	26	95.67%
2,307	3.363	27	95.67%
1,206	3.0812	28	95.67%
630	2.7994	29	95.67%
329	2.5175	30	95.67%
172	2.2357	31	95.67%
90	1.9539	32	95.67%
47	1.6721	33	95.67%
25	1.3902	34	95.67%
13	1.1084	35	N/A

values highlighted in light blue are below limits of detection

Limit of Detection (LOD)

The LOD was established using serial dilutions of the TrueMark Comprehensive Microbiota Control through extinction, then using the lowest reproduceable dilution and using 2-sigma (2Σ) analysis.

The C_{rt} value is 2Σ from the average of each target detected at their highest dilution, each of which has a slightly different C_{rt} cutoff. Concerns over PCR include sensitivity that is beyond the scope of clinical significance vs. absolute presence. The clinical validation was done in close comparison with microbiology results to ensure a minimum of 10K colony count was captured, but also to ensure that subclinical results do not dilute the clinical value of the assay.

The C_{rt} values are typically lower in OpenArray technology when compared to similar or identical primer-probe designs in 96-well or 384-well assays. The C_{rt} values in OpenArray are relative within the OpenArray technology.

PCR Values and Efficiency[1]

Our analysis utilized the geometric method to calculate PCR efficiency; slope-PCR efficiency is presented for comparison purposes only.

Organisms	Standard Curve Equation	R^2	Slope-PCR Efficiency	Geometric PCR Efficiency
A. baumannii_Ba04932084_s1	y=-3.3992x + 39.019	0.9838	96.88%	98.44%
C. albicans_Fn04646233_s1	y=-3.5323x + 37.756	0.9833	91.90%	95.96%
C. freundii_Ba04932088_s1	y=-3.6155x + 39.142	0.9780	89.07%	95.96%
E. aerogenes_Ba04932080_s1	y=-3.8743x + 40.370	0.9858	81.19%	90.59%
E. cloacae_Ba04932087_s1	y=-3.2603x + 37.785	0.9807	102.65%	101.32%
E. faecalis_Ba04646247_s1	y=-3.5057x + 38.040	0.9855	92.89%	96.43%
E. faecium_Ba04932086_s1	y=-3.7091x + 40.062	0.9798	86.04%	93.02%
E. coli_Ba04646242_s1	y=-3.5483x + 38.933	0.9810	91.36%	95.67%
K. oxytoca_Ba04932079_s1	y=-3.1939x + 37.757	0.9828	105.67%	102.82%
K. pneumoniae_Ba04932083_s1	y=-3.5564x + 38.391	0.9891	91.08%	95.53%
M. morganii_Ba04932078_s1	y=-3.3192x + 37.689	0.9816	100.12%	100.06%
ureR_Ba04932076_s1	y=-3.5816x + 39.733	0.9554	90.22%	100.06%
P. stuartii_Ba04932077_s1	y=-3.3935x + 37.890	0.9912	97.12%	98.55%
P. aeruginosa_Ba04932081_s1	y=-3.2156x + 37.431	0.9810	104.62%	102.32%
S. aureus_Ba04646259_s1	y=-3.5243x + 38.079	0.9872	92.03%	96.10%
S. saprophyticus_Ba04932085_s1	y=-3.5007x + 38.915	0.9538	93.07%	96.10%
S. agalactiae_Ba04646276_s1	y=-3.3268x + 38.915	0.9838	99.79%	99.90%
C. koseri_AIX02UH	y=-3.3272x + 38.377	0.9838	99.79%	99.89%
femA_Ba04230918_s1	y=-3.5816x + 38.454	0.9796	90.18%	96.83%
S. lugdunensis_APTZ9W7	y=-3.4817x + 38.484	0.9903	93.76%	96.87%
S. haemolyticus_APMFXMX	y=-3.5922x + 38.432	0.9909	89.84%	94.92%
S. marcescens_AIMSIYA	y=-3.4144x + 38.876	0.985	96.30%	98.14%
C. amalonaticus_AP7DR2J	y=-3.7084x + 38.732	0.9897	86.08%	93.03%
S. oralis_AP9HJTH	y=-3.4706x + 38.044	0.9684	94.13%	97.07%
C. glabrata_Fn04646240_s1	y=-3.5695x + 39.381	0.9926	90.63%	95.31%
M. tuberculosis_APEPTGE	y=-3.4032x + 37.243	0.9917	96.72%	98.36%
M. genitalium_Ba04646251_s1	y=-3.6815x + 39.218	0.994	86.89%	93.45%
M. hominis_Ba04646255_s1	y=-3.605x + 38.08	0.9879	89.41%	94.70%
P. agglomerans_AP47WMK	y=-3.5181x + 38.977	0.989	92.42%	96.21%
U. urealyticum_Ba04646254_s1	y=-3.2829x + 36.706	0.9872	101.65%	100.83%
CMV_Pa03453400_s1	y=-3.3914x + 37.995	0.9732	97.20%	98.59%
HSV1_Vi04230116_s1	y=-3.3675x + 38.597	0.9978	98.11%	99.07%
HSV2_Vi04646232_s1	y=-3.3449x + 37.147	0.9876	99.05%	99.53%
A. schaalii_AIPAFMX	y=-3.34829x + 38.598	0.9911	98.92%	96.85%
A. urinae_AIQJDS5	y=-3.3424x + 37.28	0.9819	99.17%	99.58%
A. omnicolens_AIVI6H1	y=-3.3545x + 37.591	0.9934	98.63%	99.33%
C. parapsilosis_Fn04646221_s1	y=-3.1613x + 37.365	0.9661	107.18%	103.58%
C. riegelii_AI5IRVT	y=-3.4318x + 38.134	0.9851	95.64%	97.81%
C. urealyticum_AI39TPL	y=-3.4644x + 38.825	0.9876	94.39%	97.19%
C. trachomatis_Ba04646249_s1	y=-3.3189x + 37.56	0.9874	100.16%	100.06%
N. gonorrhoeae_Ba04646252_s1	y=-3.6217x + 39.282	0.9859	88.84%	94.42%
T. vaginalis_Pr04646256_s1	y=-3.3929x + 38.757	0.9895	97.16%	98.56%
S. pasteuranus_APCE4P6	y=-3.5188x + 39.603	0.9884	92.42%	96.20%
S. pyogenes_AIVI6AD	y=-3.4669x + 38.708	0.9907	94.28%	97.14%
HHV6_AI1RW3J	y=-3.418x + 38.657	0.9817	96.14%	98.07%
HHV7-RK_Vi03453404_s1	y=-3.4439x + 38.18	0.9908	95.15%	97.57%

[1]*based on the regression line of the average Crt-value for each target organism, with formula y=mx+b where y is defined as the C_{rt}-value and x is defined as the number of DNA copies/2.5 µL*

List of Antibiotic Resistance Genes on UTI OpenArray Panel

ThermoFisher ABR Assay	ThermoFisher Assay ID
VanA	Ba04646147_s1
β-lactamase (class D)	Ba04646133_s1
AmpC β-lactamase (Class C)	Ba04646126_s1
Carbapenemase (Class A)	Ba04646151_s1
Carbapenemase (Class A)	Ba04646152_s1
Carbapenemase (Class B)	Ba04646131_s1
Carbapenemase (Class B)	Ba04931076_s1
Carbapenemase (Class D)	Ba04930816_s1
Extended Spectrum β-lactamase (Class A)	Ba04646140_s1
Extended Spectrum β-lactamase (Class A)	Ba04646153_s1
Extended-Spectrum-β-Lactamase	Ba04646149_s1
Extended-Spectrum-β-Lactamase	Ba04646142_s1
Extended-Spectrum-β-Lactamase	Ba04646154_s1
Extended-Spectrum-β-Lactamase	Ba04646127_s1
Macrolide Resistance	Pa04230913_s1
Quinolone Resistance	Ba04646160_s1
Quinolone Resistance	Ba04646145_s1
verona integron-encoded metallo-β-lactamase	Ba04646155_s1

Antibiotic Resistance Gene CARD Database Location

The chart in Supplement C provides the antibiotic resistance gene symbol, gene name, ThermoFisher Assay ID, and references to the Comprehensive Antibiotic Resistance Database (CARD). The CARD database link, drug class or classes, and resistance mechanism are described for each. Drug class or classes and resistance mechanism data are derived directly from the CARD database (https://card.mcmaster.ca/).

References

1. Lantz, P.G. et al. (1997) Removal of PCR inhibitors from human faecal samples through the use of an aqueous two-phase system for sample preparation prior to PCR. J. Microbiol. Meth. 28, 159–67. (Bile salts)
2. Al-Soud, W.A. and Rådström, P. (2001) Purification and characterization of PCR inhibitory components in blood cells. J. Clin. Microbiol. 39, 485–93. (Hemoglobin)
3. Bickley, J. et al. (1996) Polymerase chain reaction (PCR) detection of Listeria monocytogenes in diluted milk and reversal of PCR inhibition caused by calcium ions. Lett., Appl. Microbiol. 22, 153–8.
4. "Publish Real-Time PCR Results with Confidence." Accessed at https://www.thermofisher.com/us/en/home/life-science/pcr/real-time-pcr/real-time-pcr-assays/why-choose-taqman-assays/publish-real-time-pcr-results.html.

The Impact of Polymerase Chain Reaction Urine Testing on Clinical Decision-Making in the Management of Complex Urinary Tract Infections

Supplement B – PCR Efficiency and Linear Range

Julia Elia, Jason Hafron, Mara Holton, Connor Ervin, Mitchell B. Hollander, and Deepak A. Kapoor

A. baumannii	1st C_{rt} Value	2nd C_{rt} Value	3rd C_{rt} Value	Average C_{rt} Value
125,000 copies/2.5 µL (1:1000)	21.395	21.698	21.425	21.506
25,000 copies/2.5 µL (1:5000)	24.580	24.773	24.425	24.593
12,500 copies/2.5 µL (1:10,000)	24.918	24.687	25.066	24.890
1,250 copies/2.5 µL (1:100,000)	27.734	28.322	28.016	28.024
625 copies/2.5 µL (1:200,000)	29.297	29.575	30.684	29.852

Logarithmic curve	
DNA copies/2.5 µL	C_{rt} value
125,000	21.506
25,000	24.593
12,500	24.890
1,250	28.024
625	29.852

Standard curve data of average C_{rt} value		
DNA copies/2.5 µL	log DNA copies/ 2.5 µL	C_{rt} value
125,000	5.096910013	21.506
25,000	4.397940009	24.593
12,500	4.096910013	24.890
1,250	3.096910013	28.024
625	2.795880017	29.852

* Using the average Ct-value mean resulted in the same equation.

y=3.3992x + 39.019			
DNA copies/2.5 µL	log DNA copies/2.5µL (x)	C_{rt} value (y)	PCR Efficiency
11,643,254	7.0661	15	98.44%
5,914,084	6.7719	16	98.44%
3,004,005	6.4777	17	98.44%
1,525,857	6.1835	18	98.44%
775,045	5.8893	19	98.44%
393,677	5.5951	20	98.44%
199,965	5.3010	21	98.44%
101,570	5.0068	22	98.44%
51,592	4.7126	23	98.44%
26,206	4.4184	24	98.44%
13,311	4.1242	25	98.44%
6,761	3.8300	26	98.44%
3,434	3.5358	27	98.44%
1,744	3.2416	28	98.44%
886	2.9475	29	98.44%
450	2.6533	30	98.44%
229	2.3591	31	98.44%
116	2.0649	32	98.44%
59	1.7707	33	98.44%
30	1.4765	34	98.44%
15	1.1823	35	N/A

** cells highlighted in light blue are below detection threshold limit*

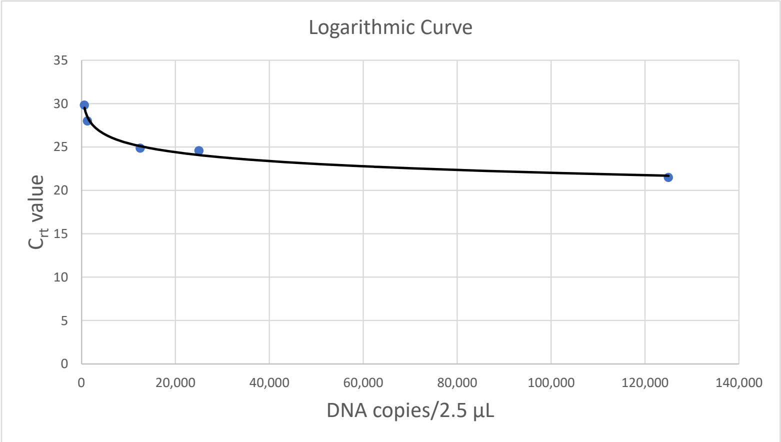

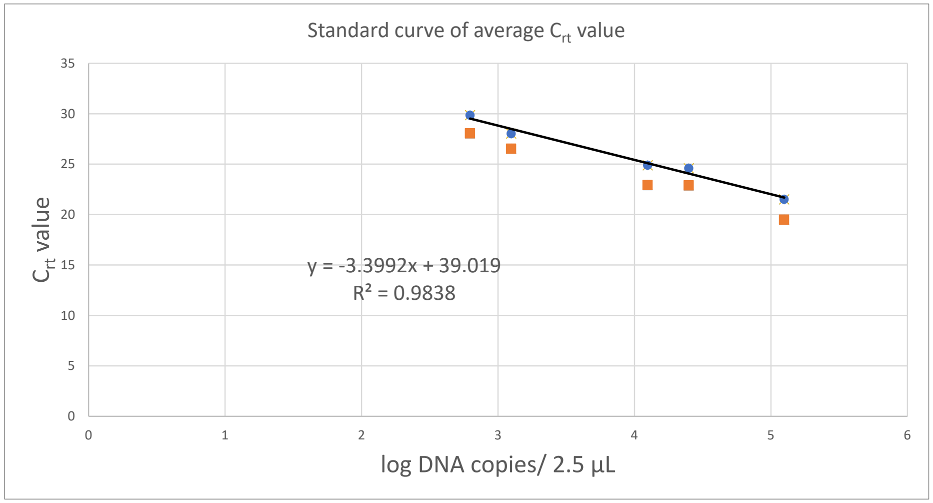

C. Albicans	1st C_{rt} Value	2nd C_{rt} Value	3rd C_{rt} Value	Average C_{rt} Value
125,000 copies/2.5 µL (1:1000)	19.501	19.581	19.437	19.506
25,000 copies/2.5 µL (1:5000)	23.008	23.258	22.457	22.908
12,500 copies/2.5 µL (1:10,000)	22.873	22.977	22.967	22.939
1,250 copies/2.5 µL (1:100,000)	27.012	26.321	26.299	26.544
625 copies/2.5 µL (1:200,000)	27.953	28.024	28.203	28.060

Logarithmic curve	
DNA copies/2.5 µL	C_{rt} value
125,000	19.506
25,000	22.908
12,500	22.939
1,250	26.544
625	28.060

Standard curve data of average C_{rt} value		
DNA copies/2.5 µL	log DNA copies/ 2.5 µL	C_{rt} value
125,000	5.096910013	19.506
25,000	4.397940009	22.908
12,500	4.096910013	22.939
1,250	3.096910013	26.544
625	2.795880017	28.060

* Using the average Ct-value mean resulted in the same equation.

y=-3.5323x + 37.756			
DNA copies/2.5 µL	log DNA copies/2.5µL (x)	C_{rt} value (y)	PCR Efficiency
2,768,608	6.4423	15	95.96%
1,442,646	6.1592	16	95.96%
751,723	5.8761	17	95.96%
391,703	5.5930	18	95.96%
204,106	5.3099	19	95.96%
106,354	5.0268	20	95.96%
55,418	4.7437	21	95.96%
28,877	4.4605	22	95.96%
15,047	4.1774	23	95.96%
7,841	3.8943	24	95.96%
4,085	3.6112	25	95.96%
2,129	3.3281	26	95.96%
1,109	3.0450	27	95.96%
578	2.7619	28	95.96%
301	2.4788	29	95.96%
157	2.1957	30	95.96%
82	1.9126	31	95.96%
43	1.6295	32	95.96%
22	1.3464	33	95.96%
12	1.0633	34	95.96%
6	0.7802	35	N/A

** cells highlighted in light blue are below detection threshold limit*

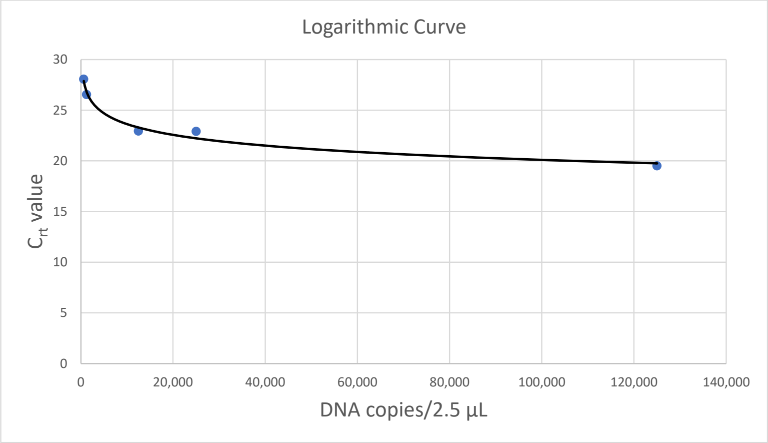

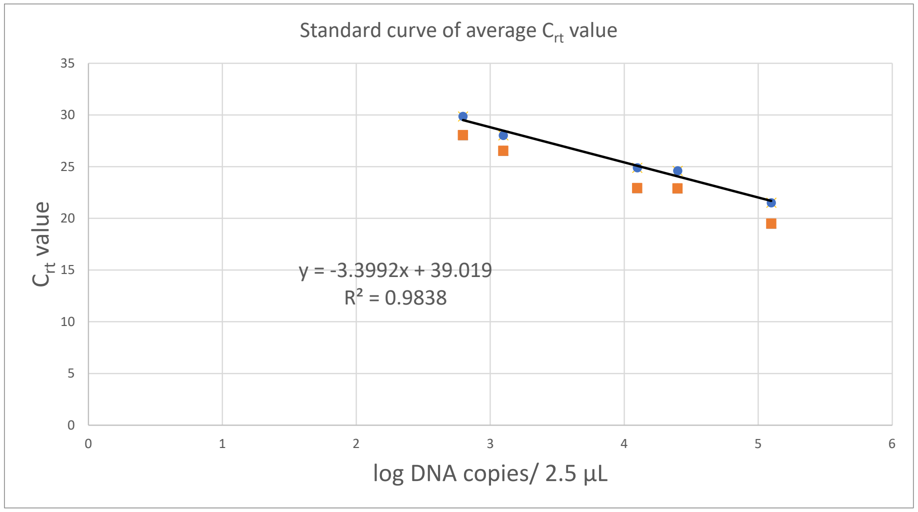

C. freundii	1st C_{rt} Value	2nd C_{rt} Value	3rd C_{rt} Value	Average C_{rt} Value
125,000 copies/2.5 µL (1:1000)	20.345	20.412	20.356	20.371
25,000 copies/2.5 µL (1:5000)	24.084	23.787	23.995	23.955
12,500 copies/2.5 µL (1:10,000)	23.761	24.153	23.990	23.968
1,250 copies/2.5 µL (1:100,000)	27.943	28.136	28.894	28.324
625 copies/2.5 µL (1:200,000)	29.678	28.226	28.026	28.643

Logarithmic curve	
DNA copies/2.5 µL	C_{rt} value
125,000	20.371
25,000	23.955
12,500	23.968
1,250	28.324
625	28.643

Standard curve data of average C_{rt} value		
DNA copies/2.5 µL	log DNA copies/ 2.5 µL	C_{rt} value
125,000	5.096910013	20.371
25,000	4.397940009	23.955
12,500	4.096910013	23.968
1,250	3.096910013	28.324
625	2.795880017	28.643

* Using the average Ct-value mean resulted in the same equation.

y=-3.6155x + 39.142			
DNA copies/2.5 µL	log DNA copies/2.5µL (x)	C_{rt} value (y)	PCR Efficiency
2,768,608	6.4423	15	95.96%
1,442,646	6.1592	16	95.96%
751,723	5.8761	17	95.96%
391,703	5.5930	18	95.96%
204,106	5.3099	19	95.96%
106,354	5.0268	20	95.96%
55,418	4.7437	21	95.96%
28,877	4.4605	22	95.96%
15,047	4.1774	23	95.96%
7,841	3.8943	24	95.96%
4,085	3.6112	25	95.96%
2,129	3.3281	26	95.96%
1,109	3.0450	27	95.96%
578	2.7619	28	95.96%
301	2.4788	29	95.96%
157	2.1957	30	95.96%
82	1.9126	31	95.96%
43	1.6295	32	95.96%
22	1.3464	33	95.96%
12	1.0633	34	95.96%
6	0.7802	35	N/A

** cells highlighted in light blue are below detection threshold limit*

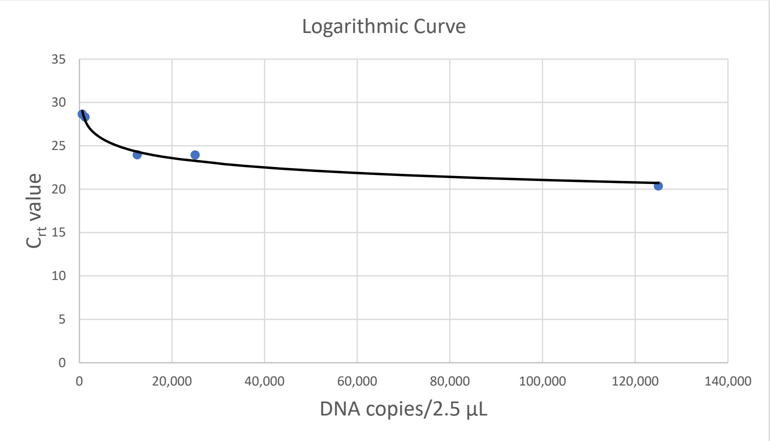

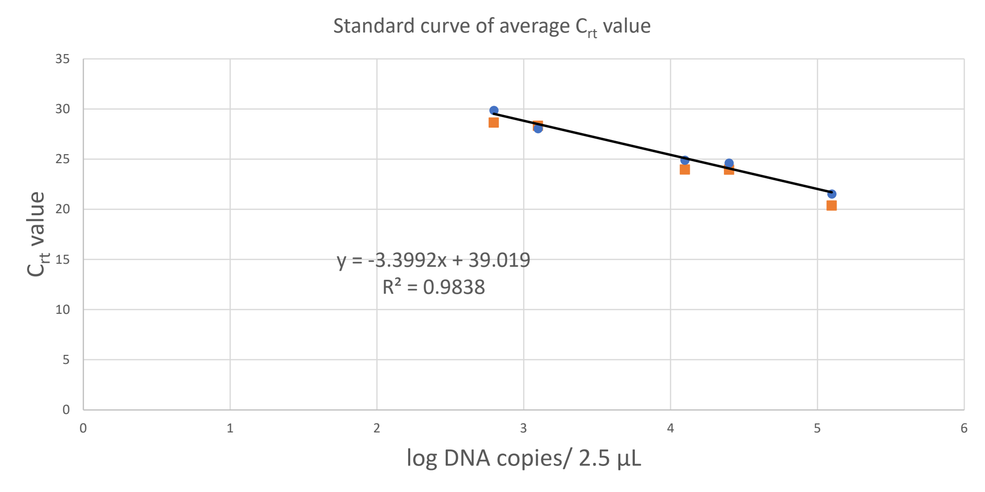

E. aerogenes	1st C_{rt} Value	2nd C_{rt} Value	3rd C_{rt} Value	Average C_{rt} Value
125,000 copies/2.5 µL (1:1000)	20.350	20.471	20.410	20.410
25,000 copies/2.5 µL (1:5000)	23.958	24.133	23.879	23.990
12,500 copies/2.5 µL (1:10,000)	23.970	24.075	24.006	24.017
1,250 copies/2.5 µL (1:100,000)	29.412	28.334	27.962	28.569
625 copies/2.5 µL (1:200,000)	30.721	28.330	29.074	29.375

Logarithmic curve	
DNA copies/2.5 µL	C_{rt} value
125,000	20.410
25,000	23.990
12,500	24.017
1,250	28.569
625	29.375

Standard curve data of average C_{rt} value		
DNA copies/2.5 µL	log DNA copies/ 2.5 µL	C_{rt} value
125,000	5.096910013	20.410
25,000	4.397940009	23.990
12,500	4.096910013	24.017
1,250	3.096910013	28.569
625	2.795880017	29.375

* Using the average Ct-value mean resulted in the same equation.

y=-3.8743x + 40.37			
DNA copies/2.5 µL	log DNA copies/2.5µL (x)	C_{rt} value (y)	PCR Efficiency
3,534,107	6.5483	15	90.59%
1,950,601	6.2902	16	90.59%
1,076,608	6.0321	17	90.59%
594,219	5.7739	18	90.59%
327,971	5.5158	19	90.59%
181,019	5.2577	20	90.59%
99,911	4.9996	21	90.59%
55,144	4.7415	22	90.59%
30,436	4.4834	23	90.59%
16,799	4.2253	24	90.59%
9,272	3.9672	25	90.59%
5,117	3.7091	26	90.59%
2,825	3.4509	27	90.59%
1,559	3.1928	28	90.59%
860	2.9347	29	90.59%
475	2.6766	30	90.59%
262	2.4185	31	90.59%
145	2.1604	32	90.59%
80	1.9023	33	90.59%
44	1.6442	34	90.59%
24	1.3861	35	N/A

** cells highlighted in light blue are below detection threshold limit*

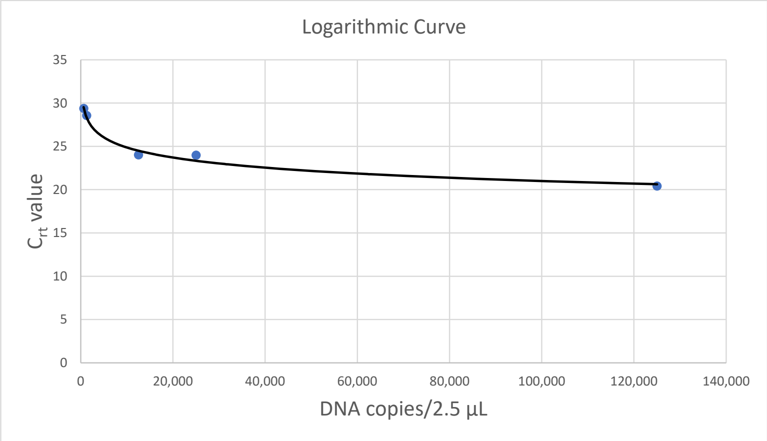

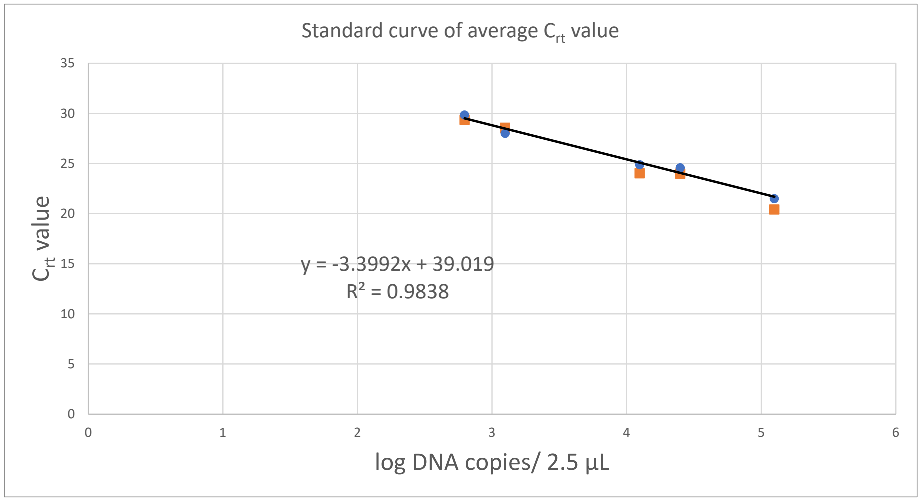

E. cloacae	1st C_{rt} Value	2nd C_{rt} Value	3rd C_{rt} Value	Average C_{rt} Value
125,000 copies/2.5 µL (1:1000)	20.456	20.829	20.981	20.755
25,000 copies/2.5 µL (1:5000)	24.017	24.395	23.607	24.006
12,500 copies/2.5 µL (1:10,000)	24.398	24.482	24.317	24.399
1,250 copies/2.5 µL (1:100,000)	27.934	28.572	27.463	27.990
625 copies/2.5 µL (1:200,000)	28.432	28.495	27.825	28.251

Logarithmic curve	
DNA copies/2.5 µL	C_{rt} value
125,000	20.755
25,000	24.006
12,500	24.399
1,250	27.990
625	28.251

Standard curve data of average C_{rt} value		
DNA copies/2.5 µL	log DNA copies/ 2.5 µL	C_{rt} value
125,000	5.096910013	20.755
25,000	4.397940009	24.006
12,500	4.096910013	24.399
1,250	3.096910013	27.990
625	2.795880017	28.251

* Using the average Ct-value mean resulted in the same equation.

y=-3.2603x + 37.785			
DNA copies/2.5 µL	log DNA copies/2.5µL (x)	C_{rt} value (y)	PCR Efficiency
9,741,384	6.9886	15	100.00%
4,807,291	6.6819	16	100.00%
2,372,358	6.3752	17	100.00%
1,170,739	6.0685	18	100.00%
577,750	5.7617	19	100.00%
285,115	5.4550	20	100.00%
140,702	5.1483	21	100.00%
69,435	4.8416	22	100.00%
34,266	4.5349	23	100.00%
16,910	4.2281	24	100.00%
8,345	3.9214	25	100.00%
4,118	3.6147	26	100.00%
2,032	3.3080	27	100.00%
1,003	3.0013	28	100.00%
495	2.6945	29	100.00%
244	2.3878	30	100.00%
121	2.0811	31	100.00%
59	1.7744	32	100%
29	1.4677	33	100%
14	1.1609	34	100%
7	0.8542	35	N/A

** cells highlighted in light blue are below detection threshold limit*

*** PCR efficiency is a calculation where the theoretical maximum is 100%. If the equation has calculated a PCR efficiency over 100%, the maximum value of 100% is represented.*

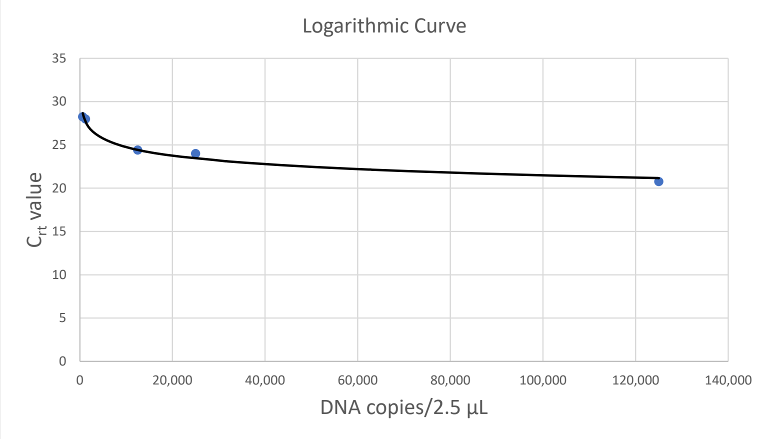

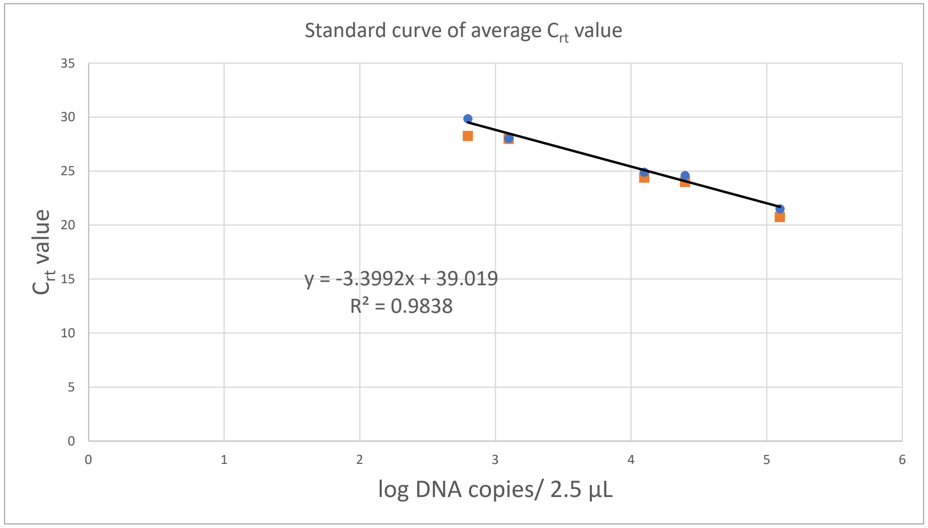

E. faecalis	1st C_{rt} Value	2nd C_{rt} Value	3rd C_{rt} Value	Average C_{rt} Value
125,000 copies/2.5 µL (1:1000)	19.907	20.010	19.872	19.930
25,000 copies/2.5 µL (1:5000)	23.615	23.525	22.656	23.265
12,500 copies/2.5 µL (1:10,000)	23.445	23.476	23.193	23.371
1,250 copies/2.5 µL (1:100,000)	26.608	27.697	26.508	26.938
625 copies/2.5 µL (1:200,000)	27.443	28.596	29.131	28.390

Logarithmic curve	
DNA copies/2.5 µL	C_{rt} value
125,000	19.930
25,000	23.265
12,500	23.371
1,250	26.938
625	28.390

Standard curve data of average C_{rt} value		
DNA copies/2.5 µL	log DNA copies/ 2.5 µL	C_{rt} value
125,000	5.096910013	19.930
25,000	4.397940009	23.265
12,500	4.096910013	23.371
1,250	3.096910013	26.938
625	2.795880017	28.390

* Using the average Ct-value mean resulted in the same equation.

y=-3.5057x + 38.04

DNA copies/2.5 µL	log DNA copies/2.5µL (x)	C_{rt} value (y)	PCR Efficiency
3,733,825	6.5722	15	96.43%
1,935,995	6.2869	16	96.43%
1,003,817	6.0017	17	96.43%
520,481	5.7164	18	96.43%
269,870	5.4312	19	96.43%
139,928	5.1459	20	96.43%
72,553	4.8607	21	96.43%
37,619	4.5754	22	96.43%
19,505	4.2902	23	96.43%
10,114	4.0049	24	96.43%
5,244	3.7197	25	96.43%
2,719	3.4344	26	96.43%
1,410	3.1492	27	96.43%
731	2.8639	28	96.43%
379	2.5787	29	96.43%
197	2.2934	30	96.43%
102	2.0082	31	96.43%
53	1.7229	32	96.43%
27	1.4377	33	96.43%
14	1.1524	34	96.43%
7	0.8672	35	N/A

** cells highlighted in light blue are below detection threshold limit*

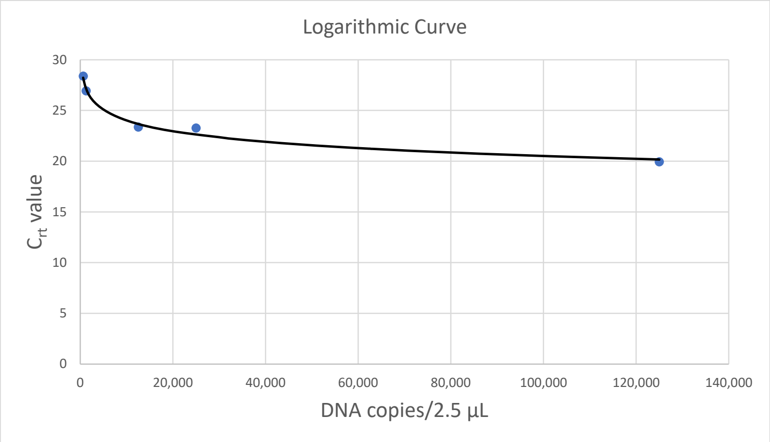

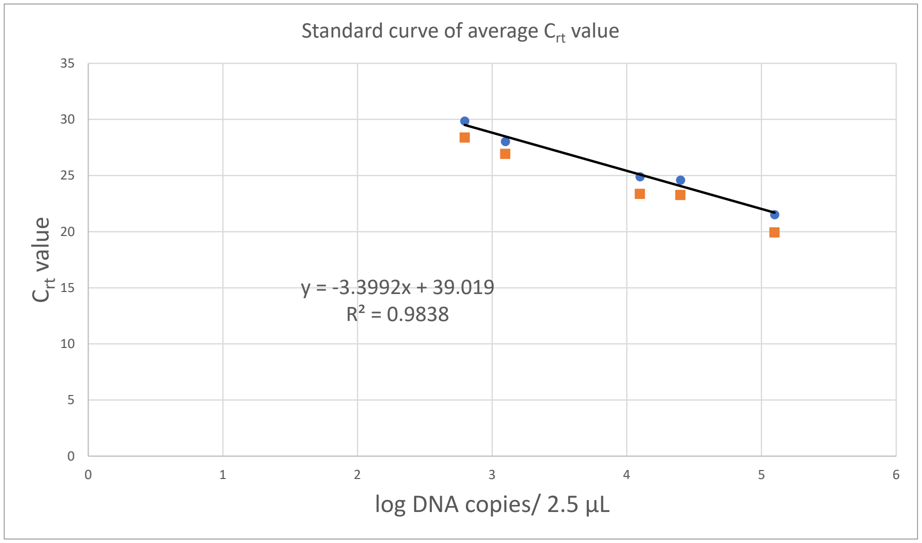

E. faecium	1st C_{rt} Value	2nd C_{rt} Value	3rd C_{rt} Value	Average C_{rt} Value
125,000 copies/2.5 µL (1:1000)	20.850	20.910	20.709	20.823
25,000 copies/2.5 µL (1:5000)	24.980	24.653	24.035	24.556
12,500 copies/2.5 µL (1:10,000)	24.347	24.630	24.649	24.542
1,250 copies/2.5 µL (1:100,000)	27.589	28.122	28.996	28.236
625 copies/2.5 µL (1:200,000)	29.450	29.668	30.530	29.883

Logarithmic curve	
DNA copies/2.5 µL	C_{rt} value
125,000	20.823
25,000	24.556
12,500	24.542
1,250	28.236
625	29.883

Standard curve data of average C_{rt} value		
DNA copies/2.5 µL	log DNA copies/ 2.5 µL	C_{rt} value
125,000	5.096910013	20.823
25,000	4.397940009	24.556
12,500	4.096910013	24.542
1,250	3.096910013	28.236
625	2.795880017	29.883

* Using the average Ct-value mean resulted in the same equation.

y=-3.7091x + 40.062			
DNA copies/2.5 µL	log DNA copies/2.5µL (x)	C_{rt} value (y)	PCR Efficiency
5,713,408	6.7569	15	93.02%
3,071,058	6.4873	16	93.02%
1,650,748	6.2177	17	93.02%
887,306	5.9481	18	93.02%
476,943	5.6785	19	93.02%
256,365	5.4089	20	93.02%
137,801	5.1393	21	93.02%
74,070	4.8696	22	93.02%
39,814	4.6000	23	93.02%
21,401	4.3304	24	93.02%
11,503	4.0608	25	93.02%
6,183	3.7912	26	93.02%
3,324	3.5216	27	93.02%
1,786	3.2520	28	93.02%
960	2.9824	29	93.02%
516	2.7128	30	93.02%
277	2.4432	31	93.02%
149	2.1736	32	93.02%
80	1.9040	33	93.02%
43	1.6344	34	93.02%
23	1.3648	35	N/A

** cells highlighted in light blue are below detection threshold limit*

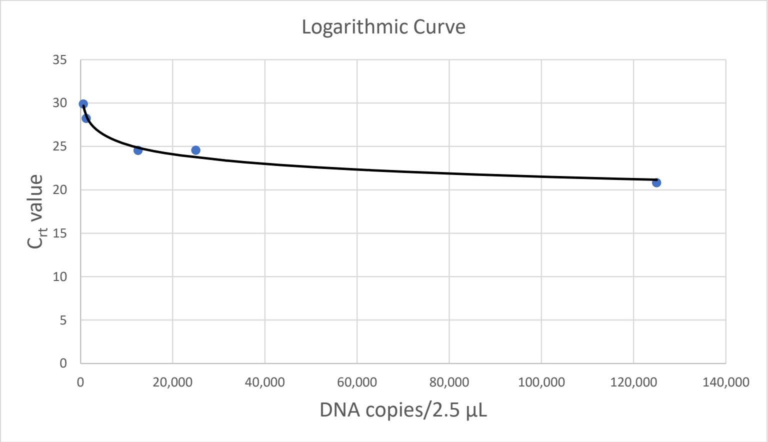

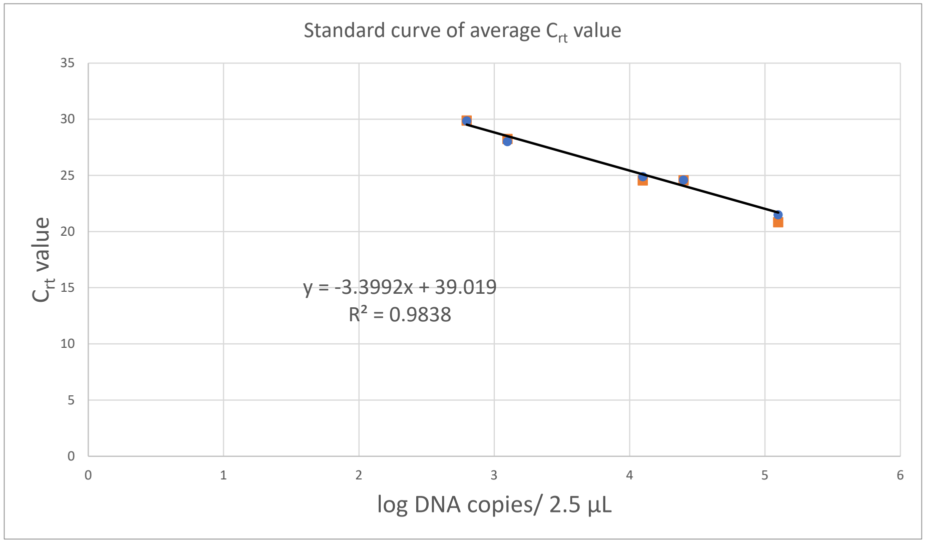

E.coli	1st C_{rt} Value	2nd C_{rt} Value	3rd C_{rt} Value	Average C_{rt} Value
125,000 copies/2.5 µL (1:1000)	20.627	20.594	20.594	20.605
25,000 copies/2.5 µL (1:5000)	24.109	24.305	23.806	24.073
12,500 copies/2.5 µL (1:10,000)	24.043	23.755	24.069	23.956
1,250 copies/2.5 µL (1:100,000)	27.641	27.203	28.351	27.732
625 copies/2.5 µL (1:200,000)	30.334	28.831	28.320	29.162

Logarithmic curve	
DNA copies/2.5 µL	C_{rt} value
125,000	20.605
25,000	24.073
12,500	23.956
1,250	27.732
625	29.162

Standard curve data of average C_{rt} value		
DNA copies/2.5 µL	log DNA copies/ 2.5 µL	C_{rt} value
125,000	5.096910013	20.605
25,000	4.397940009	24.073
12,500	4.096910013	23.956
1,250	3.096910013	27.732
625	2.795880017	29.162

* Using the average Ct-value mean resulted in the same equation.

y=-3.5483x + 38.933			
DNA copies/2.5 µL	log DNA copies/2.5µL (x)	C_{rt} value (y)	PCR Efficiency
5,558,020	6.7449	15	95.67%
2,904,658	6.4631	16	95.67%
1,517,993	6.1813	17	95.67%
793,313	5.8994	18	95.67%
414,591	5.6176	19	95.67%
216,668	5.3358	20	95.67%
113,232	5.0540	21	95.67%
59,176	4.7721	22	95.67%
30,926	4.4903	23	95.67%
16,162	4.2085	24	95.67%
8,446	3.9267	25	95.67%
4,414	3.6448	26	95.67%
2,307	3.3630	27	95.67%
1,206	3.0812	28	95.67%
630	2.7994	29	95.67%
329	2.5175	30	95.67%
172	2.2357	31	95.67%
90	1.9539	32	95.67%
47	1.6721	33	95.67%
25	1.3902	34	95.67%
13	1.1084	35	N/A

** cells highlighted in light blue are below detection threshold limit*

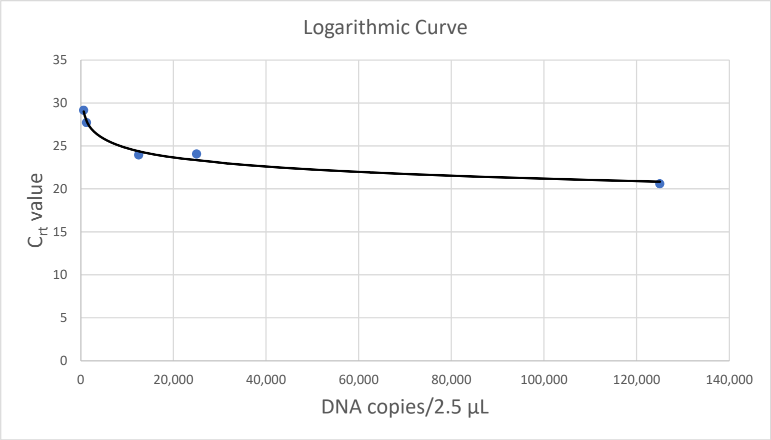

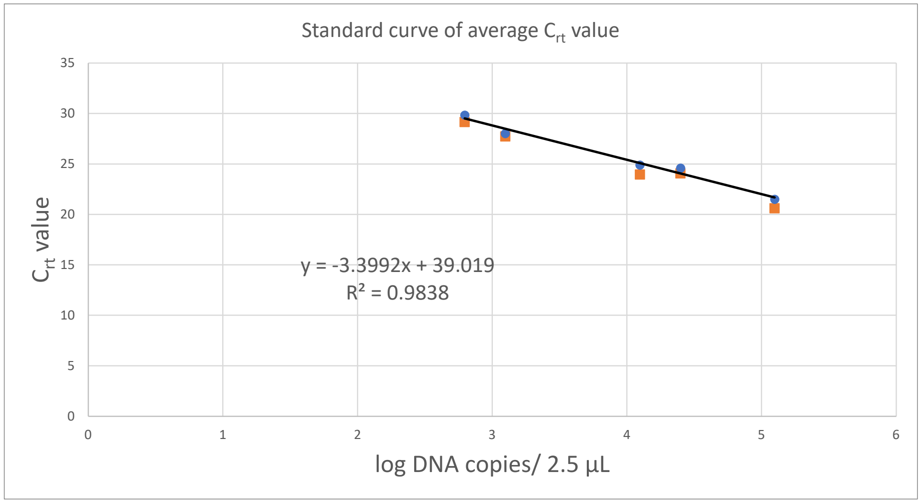

K. Oxytoca	1st C_{rt} Value	2nd C_{rt} Value	3rd C_{rt} Value	Average C_{rt} Value
125,000 copies/2.5 µL (1:1000)	20.946	21.316	21.000	21.087
25,000 copies/2.5 µL (1:5000)	24.700	24.441	24.018	24.386
12,500 copies/2.5 µL (1:10,000)	24.672	24.472	24.479	24.541
1,250 copies/2.5 µL (1:100,000)	28.341	27.570	27.565	27.825
625 copies/2.5 µL (1:200,000)	28.603	29.162	28.379	28.715

Logarithmic curve	
DNA copies/2.5 µL	C_{rt} value
125,000	21.087
25,000	24.386
12,500	24.541
1,250	27.825
625	28.715

Standard curve data of average C_{rt} value		
DNA copies/2.5 µL	log DNA copies/ 2.5 µL	C_{rt} value
125,000	5.096910013	21.087
25,000	4.397940009	24.386
12,500	4.096910013	24.541
1,250	3.096910013	27.825
625	2.795880017	28.715

* Using the average Ct-value mean resulted in the same equation.

y=-3.1939x + 37.757			
DNA copies/2.5 µL	log DNA copies/2.5µL (x)	C_{rt} value (y)	PCR Efficiency
13,339,661	7.1251	15	100.00%
6,487,061	6.8120	16	100.00%
3,154,650	6.4990	17	100.00%
1,534,102	6.1859	18	100.00%
746,032	5.8728	19	100.00%
362,794	5.5597	20	100.00%
176,426	5.2466	21	100.00%
85,796	4.9335	22	100.00%
41,722	4.6204	23	100.00%
20,290	4.3073	24	100.00%
9,867	3.9942	25	100.00%
4,798	3.6811	26	100.00%
2,333	3.3680	27	100.00%
1,135	3.0549	28	100.00%
552	2.7418	29	100.00%
268	2.4287	30	100.00%
130	2.1156	31	100.00%
63	1.8025	32	100.00%
31	1.4894	33	100.00%
15	1.1763	34	100.00%
7	0.8632	35	N/A

** cells highlighted in light blue are below detection threshold limit*

*** PCR efficiency is a calculation where the theoretical maximum is 100%. If the equation has calculated a PCR efficiency over 100%, the maximum value of 100% is represented.*

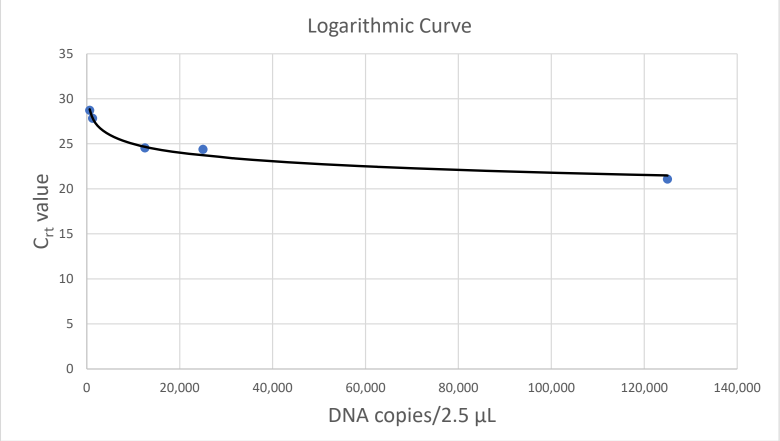

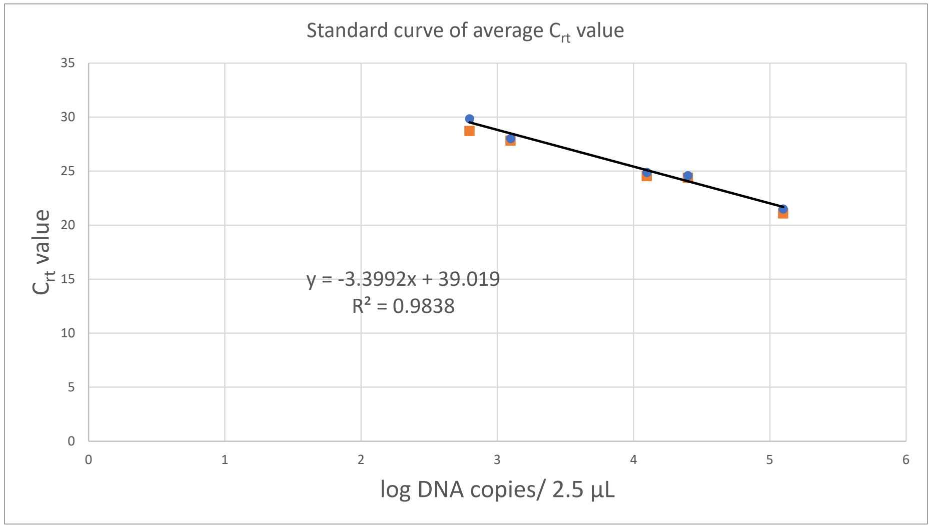

K. pneumoniae	1st C_{rt} Value	2nd C_{rt} Value	3rd C_{rt} Value	Average C_{rt} Value
125,000 copies/2.5 µL (1:1000)	19.917	20.149	20.059	20.042
25,000 copies/2.5 µL (1:5000)	23.548	23.643	22.722	23.304
12,500 copies/2.5 µL (1:10,000)	23.527	23.502	23.751	23.593
1,250 copies/2.5 µL (1:100,000)	27.140	27.317	26.901	27.119
625 copies/2.5 µL (1:200,000)	27.300	29.020	29.490	28.603

Logarithmic curve	
DNA copies/2.5 µL	C_{rt} value
125,000	20.042
25,000	23.304
12,500	23.593
1,250	27.119
625	28.603

Standard curve data of average C_{rt} value		
DNA copies/2.5 µL	log DNA copies/ 2.5 µL	C_{rt} value
125,000	5.096910013	20.042
25,000	4.397940009	23.304
12,500	4.096910013	23.593
1,250	3.096910013	27.119
625	2.795880017	28.603

* Using the average Ct-value mean resulted in the same equation.

y=-3.5564x + 38.391			
DNA copies/2.5 µL	log DNA copies/2.5µL (x)	C_{rt} value (y)	PCR Efficiency
3,777,084	6.5772	15	95.53%
1,976,849	6.2960	16	95.53%
1,034,642	6.0148	17	95.53%
541,511	5.7336	18	95.53%
283,416	5.4524	19	95.53%
148,334	5.1712	20	95.53%
77,635	4.8901	21	95.53%
40,633	4.6089	22	95.53%
21,266	4.3277	23	95.53%
11,130	4.0465	24	95.53%
5,825	3.7653	25	95.53%
3,049	3.4841	26	95.53%
1,596	3.2030	27	95.53%
835	2.9218	28	95.53%
437	2.6406	29	95.53%
229	2.3594	30	95.53%
120	2.0782	31	95.53%
63	1.7970	32	95.53%
33	1.5159	33	95.53%
17	1.2347	34	95.53%
9	0.9535	35	N/A

** cells highlighted in light blue are below detection threshold limit*

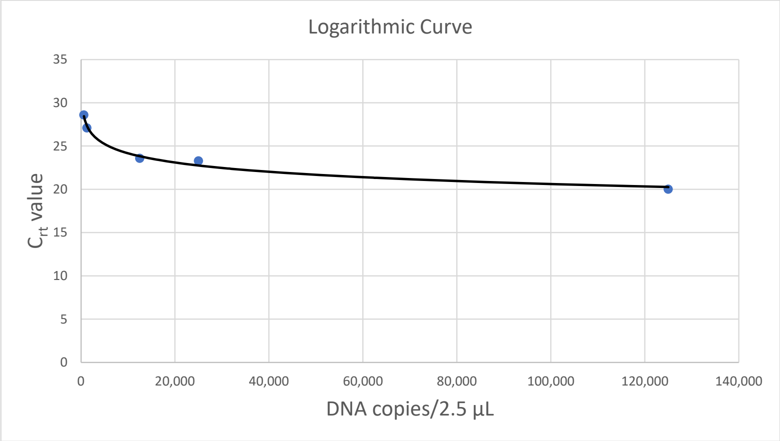

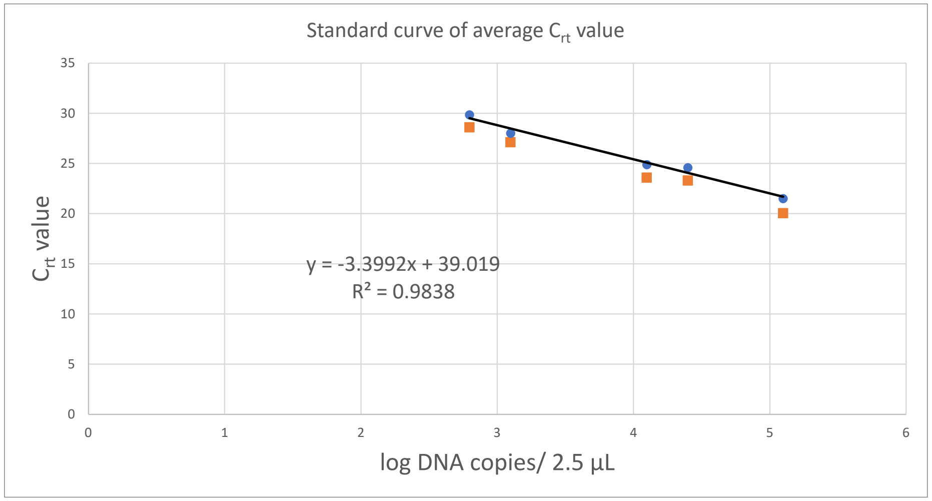

M. morganii	1st C_{rt} Value	2nd C_{rt} Value	3rd C_{rt} Value	Average C_{rt} Value
125,000 copies/2.5 µL (1:1000)	20.227	20.397	20.415	20.346
25,000 copies/2.5 µL (1:5000)	23.834	23.934	23.593	23.787
12,500 copies/2.5 µL (1:10,000)	24.148	23.706	24.044	23.966
1,250 copies/2.5 µL (1:100,000)	27.245	27.597	27.647	27.496
625 copies/2.5 µL (1:200,000)	28.192	28.086	28.252	28.177

Logarithmic curve	
DNA copies/2.5 µL	C_{rt} value
125,000	20.346
25,000	23.787
12,500	23.966
1,250	27.496
625	28.177

Standard curve data of average C_{rt} value		
DNA copies/2.5 µL	log DNA copies/ 2.5 µL	C_{rt} value
125,000	5.096910013	20.346
25,000	4.397940009	23.787
12,500	4.096910013	23.966
1,250	3.096910013	27.496
625	2.795880017	28.177

* Using the average Ct-value mean resulted in the same equation.

y=-3.3192x + 37.689

DNA copies/2.5 µL	log DNA copies/2.5µL (x)	C_{rt} value (y)	PCR Efficiency
6,849,885	6.8357	15	100.00%
3,422,992	6.5344	16	100.00%
1,710,521	6.2331	17	100.00%
854,773	5.9319	18	100.00%
427,143	5.6306	19	100.00%
213,450	5.3293	20	100.00%
106,664	5.0280	21	100.00%
53,302	4.7267	22	100.00%
26,636	4.4255	23	100.00%
13,310	4.1242	24	100.00%
6,651	3.8229	25	100.00%
3,324	3.5216	26	100.00%
1,661	3.2204	27	100.00%
830	2.9191	28	100.00%
415	2.6178	29	100.00%
207	2.3165	30	100.00%
104	2.0152	31	100.00%
52	1.7140	32	100.00%
26	1.4127	33	100.00%
13	1.1114	34	100.00%
6	0.8101	35	N/A

** cells highlighted in light blue are below detection threshold limit*

*** PCR efficiency is a calculation where the theoretical maximum is 100%. If the equation has calculated a PCR efficiency over 100%, the maximum value of 100% is represented.*

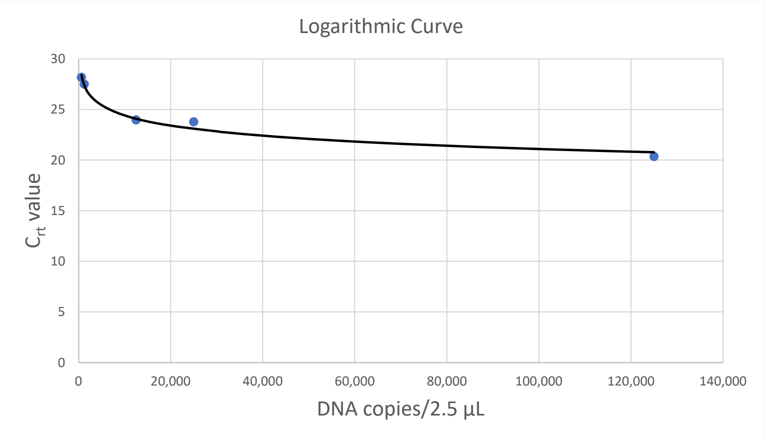

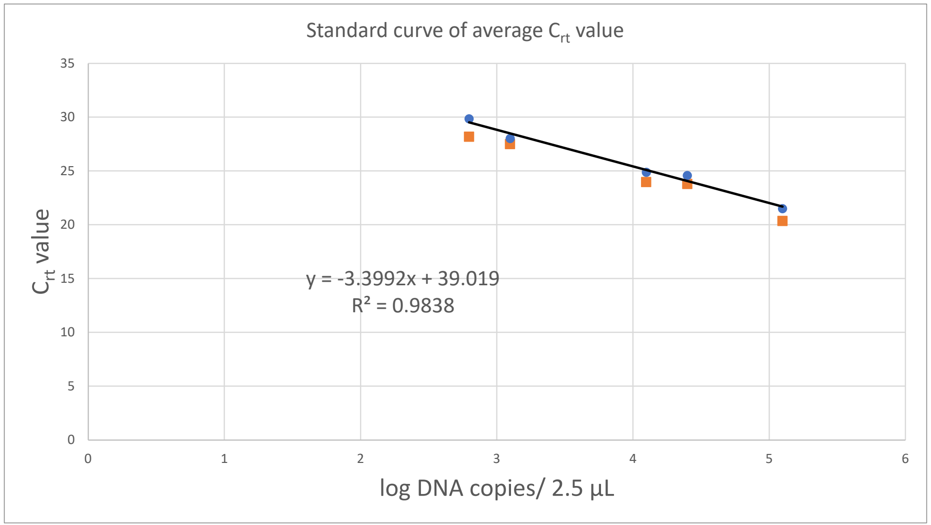

P. mirabilis	1st C_{rt} Value	2nd C_{rt} Value	3rd C_{rt} Value	Average C_{rt} Value
125,000 copies/2.5 µL (1:1000)	20.849	21.052	21.061	20.987
25,000 copies/2.5 µL (1:5000)	24.822	24.811	24.673	24.769
12,500 copies/2.5 µL (1:10,000)	24.886	24.786	24.672	24.781
1,250 copies/2.5 µL (1:100,000)	29.332	29.860	29.039	29.410
625 copies/2.5 µL (1:200,000)	28.638	28.985	29.177	28.933

Logarithmic curve	
DNA copies/2.5 µL	C_{rt} value
125,000	20.987
25,000	24.769
12,500	24.781
1,250	29.410
625	28.933

Standard curve data of average C_{rt} value		
DNA copies/2.5 µL	log DNA copies/ 2.5 µL	C_{rt} value
125,000	5.096910013	20.987
25,000	4.397940009	24.769
12,500	4.096910013	24.781
1,250	3.096910013	29.410
625	2.795880017	28.933

* Using the average Ct-value mean resulted in the same equation.

y=-3.5816x + 39.733

DNA copies/2.5 µL	log DNA copies/2.5µL (x)	C_{rt} value (y)	PCR Efficiency
6,849,885	6.8357	15	100.00%
3,422,992	6.5344	16	100.00%
1,710,521	6.2331	17	100.00%
854,773	5.9319	18	100.00%
427,143	5.6306	19	100.00%
213,450	5.3293	20	100.00%
106,664	5.0280	21	100.00%
53,302	4.7267	22	100.00%
26,636	4.4255	23	100.00%
13,310	4.1242	24	100.00%
6,651	3.8229	25	100.00%
3,324	3.5216	26	100.00%
1,661	3.2204	27	100.00%
830	2.9191	28	100.00%
415	2.6178	29	100.00%
207	2.3165	30	100.00%
104	2.0152	31	100.00%
52	1.7140	32	100.00%
26	1.4127	33	100.00%
13	1.1114	34	100.00%
6	0.8101	35	N/A

** cells highlighted in light blue are below detection threshold limit*

*** PCR efficiency is a calculation where the theoretical maximum is 100%. If the equation has calculated a PCR efficiency over 100%, the maximum value of 100% is represented.*

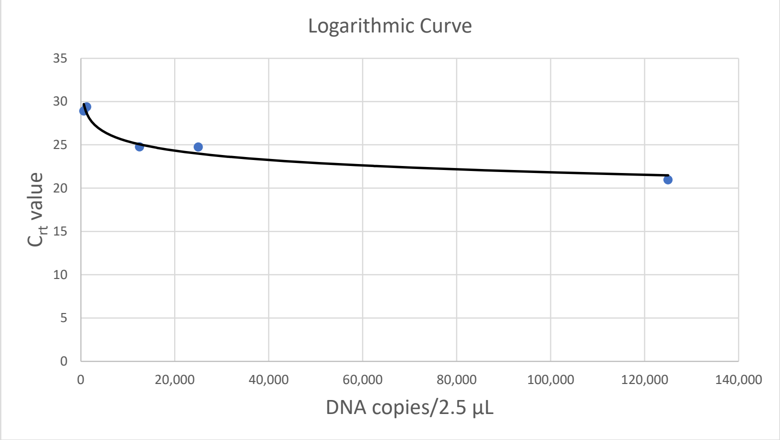

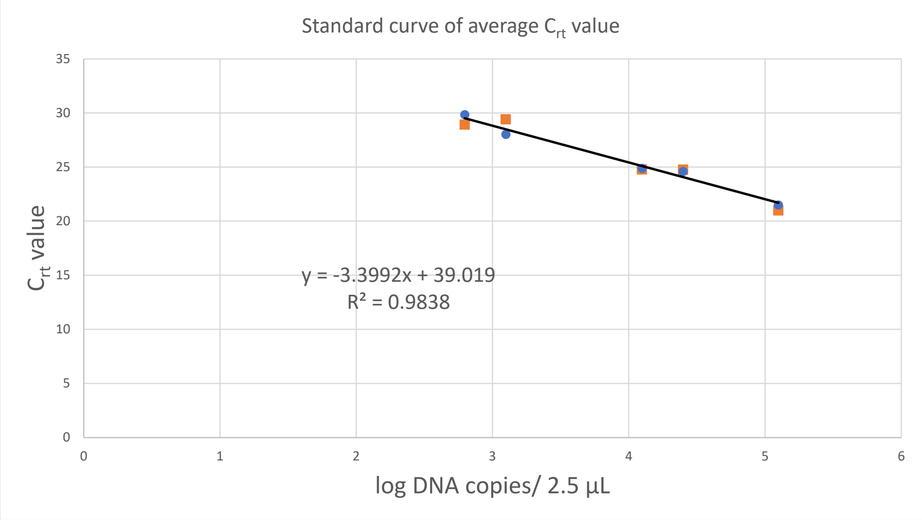

P. stuartii	1st C_{rt} Value	2nd C_{rt} Value	3rd C_{rt} Value	Average C_{rt} Value
125,000 copies/2.5 µL (1:1000)	20.334	20.564	20.381	20.426
25,000 copies/2.5 µL (1:5000)	23.668	23.527	23.170	23.455
12,500 copies/2.5 µL (1:10,000)	23.609	23.657	23.859	23.708
1,250 copies/2.5 µL (1:100,000)	26.660	28.060	27.064	27.261
625 copies/2.5 µL (1:200,000)	28.711	28.080	28.645	28.479

Logarithmic curve	
DNA copies/2.5 µL	C_{rt} value
125,000	20.426
25,000	23.455
12,500	23.708
1,250	27.261
625	28.479

Standard curve data of average C_{rt} value		
DNA copies/2.5 µL	log DNA copies/ 2.5 µL	C_{rt} value
125,000	5.096910013	20.426
25,000	4.397940009	23.455
12,500	4.096910013	23.708
1,250	3.096910013	27.261
625	2.795880017	28.479

* Using the average Ct-value mean resulted in the same equation.

y=-3.3935x + 37.890			
DNA copies/2.5 µL	log DNA copies/2.5µL (x)	C_{rt} value (y)	PCR Efficiency
5,562,221	6.7452	15	98.55%
2,822,067	6.4506	16	98.55%
1,431,813	6.1559	17	98.55%
726,449	5.8612	18	98.55%
368,574	5.5665	19	98.55%
187,001	5.2718	20	98.55%
94,877	4.9772	21	98.55%
48,137	4.6825	22	98.55%
24,423	4.3878	23	98.55%
12,391	4.0931	24	98.55%
6,287	3.7984	25	98.55%
3,190	3.5038	26	98.55%
1,618	3.2091	27	98.55%
821	2.9144	28	98.55%
417	2.6197	29	98.55%
211	2.3250	30	98.55%
107	2.0304	31	98.55%
54	1.7357	32	98.55%
28	1.4410	33	98.55%
14	1.1463	34	98.55%
7	0.8516	35	N/A

** cells highlighted in light blue are below detection threshold limit*

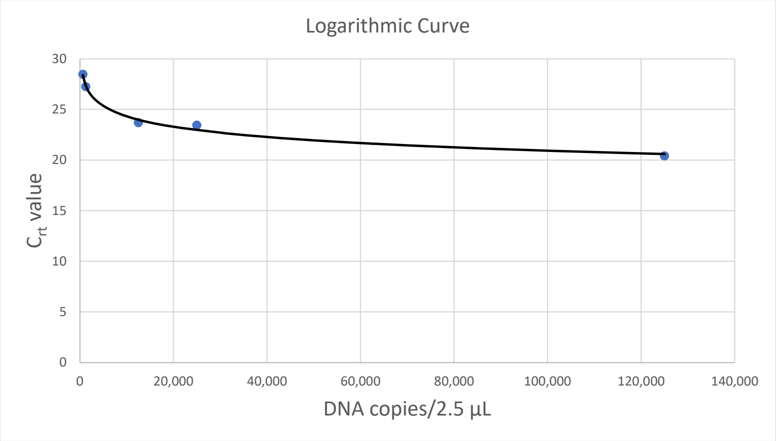

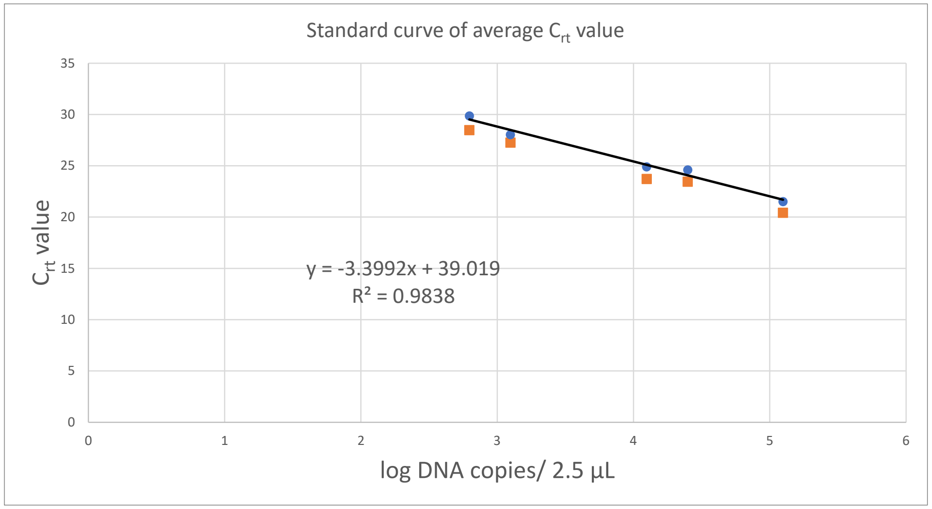

P. aeruginosa	1st C_{rt} Value	2nd C_{rt} Value	3rd C_{rt} Value	Average C_{rt} Value
125,000 copies/2.5 µL (1:1000)	20.537	20.695	20.698	20.643
25,000 copies/2.5 µL (1:5000)	24.278	23.995	23.503	23.925
12,500 copies/2.5 µL (1:10,000)	24.114	24.074	24.201	24.130
1,250 copies/2.5 µL (1:100,000)	27.922	28.020	27.071	27.671
625 copies/2.5 µL (1:200,000)	28.035	28.149	28.203	28.129

Logarithmic curve	
DNA copies/2.5 µL	C_{rt} value
125,000	20.643
25,000	23.925
12,500	24.130
1,250	27.671
625	28.129

Standard curve data of average C_{rt} value		
DNA copies/2.5 µL	log DNA copies/ 2.5 µL	C_{rt} value
125,000	5.096910013	20.643
25,000	4.397940009	23.925
12,500	4.096910013	24.130
1,250	3.096910013	27.671
625	2.795880017	28.129

* Using the average Ct-value mean resulted in the same equation.

y=-3.2156x + 37.431			
DNA copies/2.5 µL	log DNA copies/2.5µL (x)	C_{rt} value (y)	PCR Efficiency
9,455,425	6.9757	15	100.00%
4,620,586	6.6647	16	100.00%
2,257,944	6.3537	17	100.00%
1,103,390	6.0427	18	100.00%
539,194	5.7317	19	100.00%
263,488	5.4208	20	100.00%
128,759	5.1098	21	100.00%
62,921	4.7988	22	100.00%
30,747	4.4878	23	100.00%
15,025	4.1768	24	100.00%
7,342	3.8658	25	100.00%
3,588	3.5549	26	100.00%
1,753	3.2439	27	100.00%
857	2.9329	28	100.00%
419	2.6219	29	100.00%
205	2.3109	30	100.00%
100	1.9999	31	100.00%
49	1.6890	32	100.00%
24	1.3780	33	100.00%
12	1.0670	34	100.00%
6	0.7560	35	N/A

** cells highlighted in light blue are below detection threshold limit*

*** PCR efficiency is a calculation where the theoretical maximum is 100%. If the equation has calculated a PCR efficiency over 100%, the maximum value of 100% is represented.*

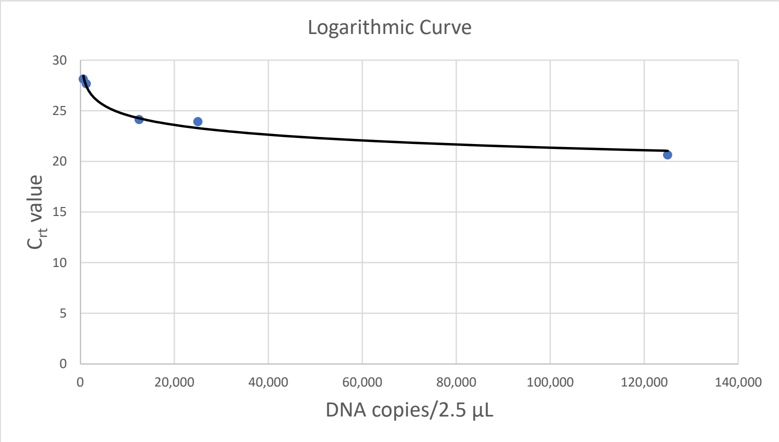

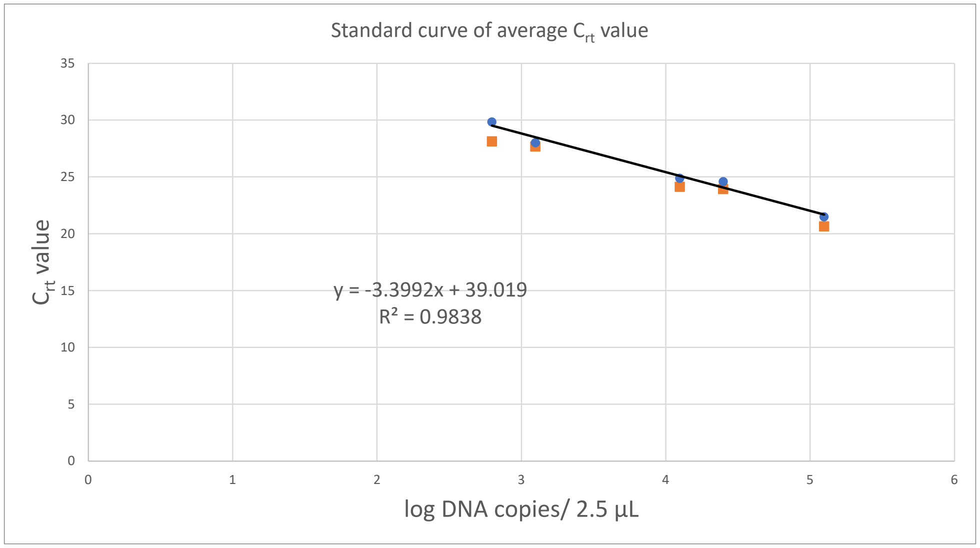

S. aureus	1st C_{rt} Value	2nd C_{rt} Value	3rd C_{rt} Value	Average C_{rt} Value
125,000 copies/2.5 µL (1:1000)	19.760	19.774	19.755	19.763
25,000 copies/2.5 µL (1:5000)	23.247	23.301	22.832	23.127
12,500 copies/2.5 µL (1:10,000)	23.563	23.573	23.481	23.539
1,250 copies/2.5 µL (1:100,000)	27.126	27.303	27.703	27.377
625 copies/2.5 µL (1:200,000)	27.605	28.656	27.490	27.917

Logarithmic curve	
DNA copies/2.5 µL	C_{rt} value
125,000	19.763
25,000	23.127
12,500	23.539
1,250	27.377
625	27.917

Standard curve data of average C_{rt} value		
DNA copies/2.5 µL	log DNA copies/ 2.5 µL	C_{rt} value
125,000	5.096910013	19.763
25,000	4.397940009	23.127
12,500	4.096910013	23.539
1,250	3.096910013	27.377
625	2.795880017	27.917

* Using the average Ct-value mean resulted in the same equation.

y=-3.5243x + 38.079			
DNA copies/2.5 µL	log DNA copies/2.5µL (x)	C_{rt} value (y)	PCR Efficiency
3,536,181	6.5485	15	96.10%
1,839,883	6.2648	16	96.10%
957,295	5.9810	17	96.10%
498,083	5.6973	18	96.10%
259,154	5.4136	19	96.10%
134,838	5.1298	20	96.10%
70,157	4.8461	21	96.10%
36,503	4.5623	22	96.10%
18,992	4.2786	23	96.10%
9,882	3.9948	24	96.10%
5,142	3.7111	25	96.10%
2,675	3.4273	26	96.10%
1,392	3.1436	27	96.10%
724	2.8599	28	96.10%
377	2.5761	29	96.10%
196	2.2924	30	96.10%
102	2.0086	31	96.10%
53	1.7249	32	96.10%
28	1.4411	33	96.10%
14	1.1574	34	96.10%
7	0.8736	35	N/A

** cells highlighted in light blue are below detection threshold limit*

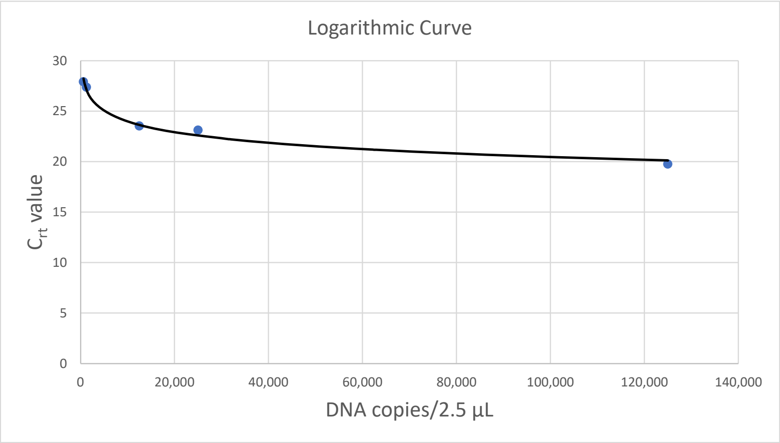

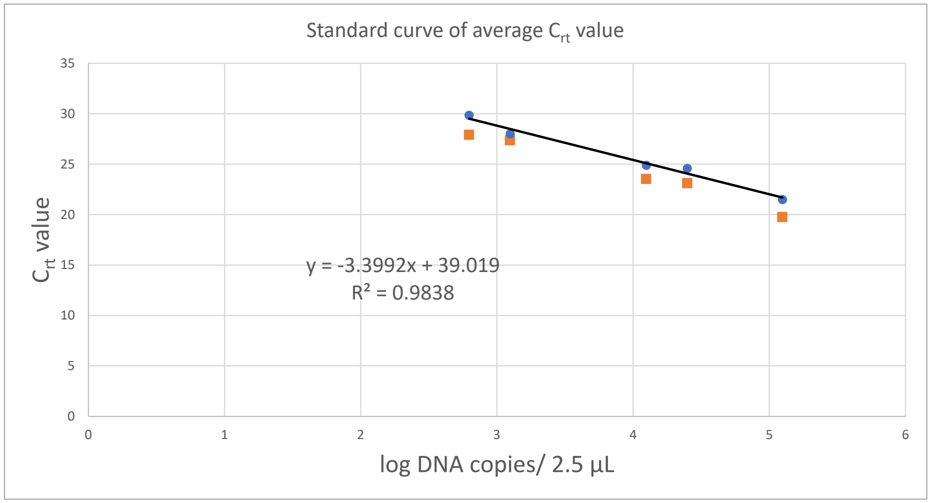

S. saprophyticus	1st C_{rt} Value	2nd C_{rt} Value	3rd C_{rt} Value	Average C_{rt} Value
125,000 copies/2.5 µL (1:1000)	20.824	20.970	21.020	20.938
25,000 copies/2.5 µL (1:5000)	24.618	24.409	24.492	24.506
12,500 copies/2.5 µL (1:10,000)	23.563	23.573	23.481	23.539
1,250 copies/2.5 µL (1:100,000)	28.618	28.130	28.222	28.323
625 copies/2.5 µL (1:200,000)	29.607	29.440	28.136	29.061

Logarithmic curve	
DNA copies/2.5 µL	C_{rt} value
125,000	20.938
25,000	24.506
12,500	23.539
1,250	28.323
625	29.061

Standard curve data of average C_{rt} value		
DNA copies/2.5 µL	log DNA copies/ 2.5 µL	C_{rt} value
125,000	5.096910013	20.938
25,000	4.397940009	24.506
12,500	4.096910013	23.539
1,250	3.096910013	28.323
625	2.795880017	29.061

* Using the average Ct-value mean resulted in the same equation.

y=-3.5007x + 38.915

DNA copies/2.5 µL	log DNA copies/2.5µL (x)	C_{rt} value (y)	PCR Efficiency
3,536,181	6.5485	15	96.10%
1,839,883	6.2648	16	96.10%
957,295	5.9810	17	96.10%
498,083	5.6973	18	96.10%
259,154	5.4136	19	96.10%
134,838	5.1298	20	96.10%
70,157	4.8461	21	96.10%
36,503	4.5623	22	96.10%
18,992	4.2786	23	96.10%
9,882	3.9948	24	96.10%
5,142	3.7111	25	96.10%
2,675	3.4273	26	96.10%
1,392	3.1436	27	96.10%
724	2.8599	28	96.10%
377	2.5761	29	96.10%
196	2.2924	30	96.10%
102	2.0086	31	96.10%
53	1.7249	32	96.10%
28	1.4411	33	96.10%
14	1.1574	34	96.10%
7	0.8736	35	N/A

** cells highlighted in light blue are below detection threshold limit*

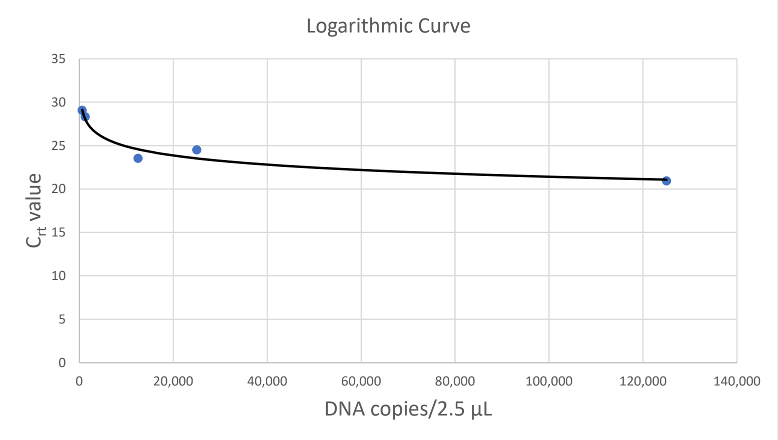

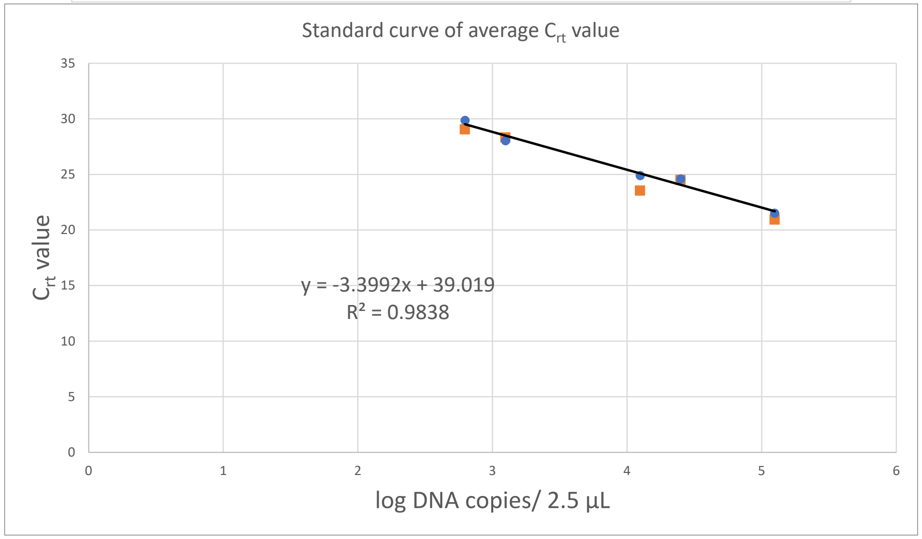

S. agalactiae	1st C_{rt} Value	2nd C_{rt} Value	3rd C_{rt} Value	Average C_{rt} Value
125,000 copies/2.5 µL (1:1000)	20.862	20.905	20.797	20.855
25,000 copies/2.5 µL (1:5000)	24.427	23.875	23.752	24.018
12,500 copies/2.5 µL (1:10,000)	24.415	24.595	24.254	24.421
1,250 copies/2.5 µL (1:100,000)	28.048	28.213	28.298	28.186
625 copies/2.5 µL (1:200,000)	28.461	28.563	28.302	28.442

Logarithmic curve	
DNA copies/2.5 µL	C_{rt} value
125,000	20.855
25,000	24.018
12,500	24.421
1,250	28.186
625	28.442

Standard curve data of average C_{rt} value		
DNA copies/2.5 µL	log DNA copies/ 2.5 µL	C_{rt} value
125,000	5.096910013	20.855
25,000	4.397940009	24.018
12,500	4.096910013	24.421
1,250	3.096910013	28.186
625	2.795880017	28.442

* Using the average Ct-value mean resulted in the same equation.

y=-3.3268x + 38.149			
DNA copies/2.5 µL	log DNA copies/2.5µL (x)	C_{rt} value (y)	PCR Efficiency
9,085,281	6.9583	15	99.90%
4,547,254	6.6577	16	99.90%
2,275,936	6.3572	17	99.90%
1,139,124	6.0566	18	99.90%
570,140	5.7560	19	99.90%
285,360	5.4554	20	99.90%
142,825	5.1548	21	99.90%
71,485	4.8542	22	99.90%
35,779	4.5536	23	99.90%
17,908	4.2530	24	99.90%
8,963	3.9524	25	99.90%
4,486	3.6519	26	99.90%
2,245	3.3513	27	99.90%
1,124	3.0507	28	99.90%
562	2.7501	29	99.90%
282	2.4495	30	99.90%
141	2.1489	31	99.90%
71	1.8483	32	99.90%
35	1.5477	33	99.90%
18	1.2471	34	99.90%
9	0.9466	35	N/A

** cells highlighted in light blue are below detection threshold limit*

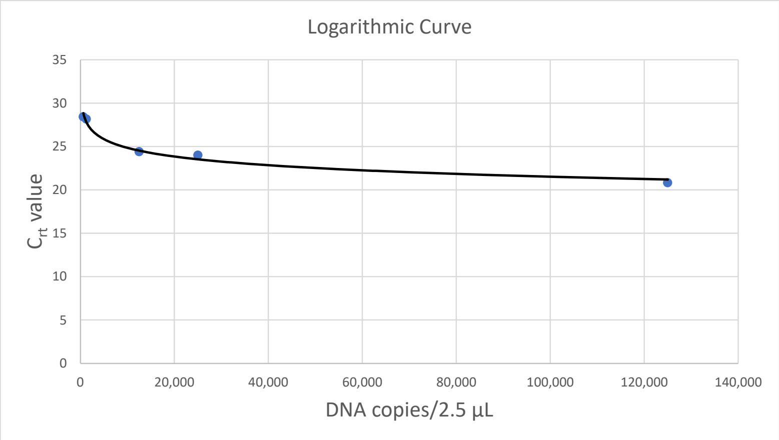

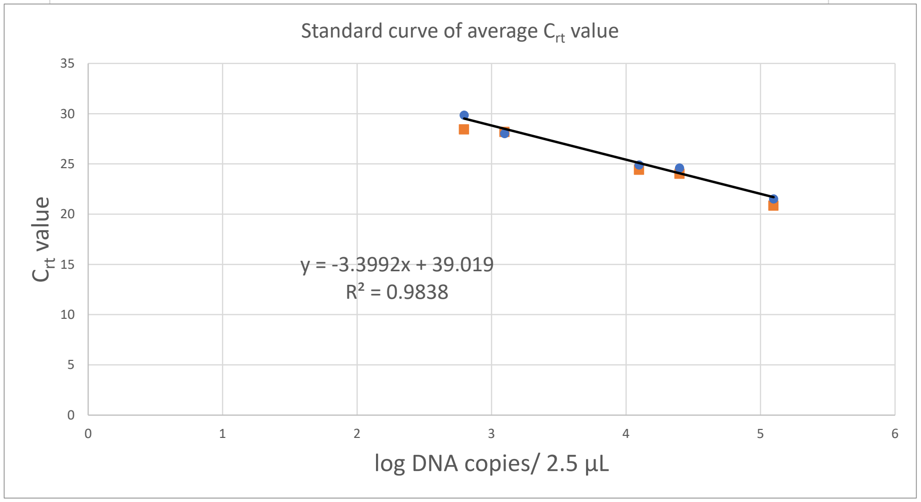

C. koseri	1st C_{rt} Value	2nd C_{rt} Value	3rd C_{rt} Value	Average C_{rt} Value
125,000 copies/2.5 µL (1:1000)	21.038	21.203	21.151	21.131
25,000 copies/2.5 µL (1:5000)	24.473	24.469	23.945	24.296
12,500 copies/2.5 µL (1:10,000)	24.595	24.577	24.551	24.574
1,250 copies/2.5 µL (1:100,000)	28.022	28.141	28.055	28.073
625 copies/2.5 µL (1:200,000)	29.379	28.852	28.712	28.981

Logarithmic curve	
DNA copies/2.5 µL	C_{rt} value
125,000	21.131
25,000	24.296
12,500	24.574
1,250	28.073
625	28.981

Standard curve data of average C_{rt} value		
DNA copies/2.5 µL	log DNA copies/ 2.5 µL	C_{rt} value
125,000	5.096910013	21.131
25,000	4.397940009	24.296
12,500	4.096910013	24.574
1,250	3.096910013	28.073
625	2.795880017	28.981

* Using the average Ct-value mean resulted in the same equation.

y=-3.3272x + 38.377			
DNA copies/2.5 µL	log DNA copies/2.5µL (x)	C_{rt} value (y)	PCR Efficiency
10,617,637	7.0260	15	99.89%
5,314,653	6.7255	16	99.89%
2,660,246	6.4249	17	99.89%
1,331,585	6.1244	18	99.89%
666,524	5.8238	19	99.89%
333,628	5.5233	20	99.89%
166,997	5.2227	21	99.89%
83,590	4.9222	22	99.89%
41,841	4.6216	23	99.89%
20,944	4.3211	24	99.89%
10,483	4.0205	25	99.89%
5,247	3.7199	26	99.89%
2,627	3.4194	27	99.89%
1,315	3.1188	28	99.89%
658	2.8183	29	99.89%
329	2.5177	30	99.89%
165	2.2172	31	99.89%
83	1.9166	32	99.89%
41	1.6161	33	99.89%
21	1.3155	34	99.89%
10	1.0150	35	N/A

** cells highlighted in light blue are below detection threshold limit*

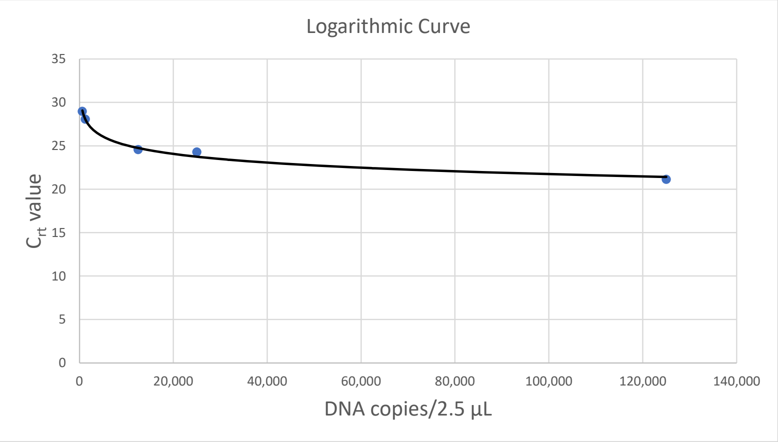

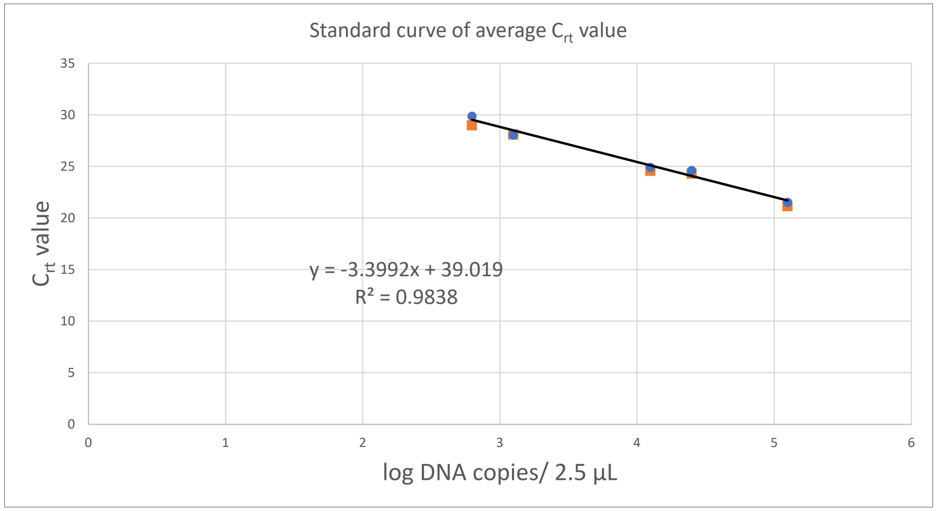

S. epidermidis	1st C_{rt} Value	2nd C_{rt} Value	3rd C_{rt} Value	Average C_{rt} Value
125,000 copies/2.5 µL (1:1000)	19.820	19.769	19.939	19.843
25,000 copies/2.5 µL (1:5000)	23.831	23.411	23.425	23.555
12,500 copies/2.5 µL (1:10,000)	23.365	23.380	23.676	23.474
1,250 copies/2.5 µL (1:100,000)	26.786	28.147	27.303	27.412
625 copies/2.5 µL (1:200,000)	29.224	28.233	27.588	28.348

Logarithmic curve	
DNA copies/2.5 µL	C_{rt} value
125,000	19.843
25,000	23.555
12,500	23.474
1,250	27.412
625	28.348

Standard curve data of average C_{rt} value		
DNA copies/2.5 µL	log DNA copies/ 2.5 µL	C_{rt} value
125,000	5.096910013	19.843
25,000	4.397940009	23.555
12,500	4.096910013	23.474
1,250	3.096910013	27.412
625	2.795880017	28.348

* Using the average Ct-value mean resulted in the same equation.

y=-3.5816x + 38.484			
DNA copies/2.5 µL	log DNA copies/2.5µL (x)	C_{rt} value (y)	PCR Efficiency
5,868,700	6.7685	15	96.83%
3,030,508	6.4815	16	96.83%
1,564,909	6.1945	17	96.83%
808,095	5.9075	18	96.83%
417,288	5.6204	19	96.83%
215,481	5.3334	20	96.83%
111,271	5.0464	21	96.83%
57,459	4.7594	22	96.83%
29,671	4.4723	23	96.83%
15,322	4.1853	24	96.83%
7,912	3.8983	25	96.83%
4,086	3.6113	26	96.83%
2,110	3.3242	27	96.83%
1,089	3.0372	28	96.83%
563	2.7502	29	96.83%
290	2.4631	30	96.83%
150	2.1761	31	96.83%
77	1.8891	32	96.83%
40	1.6021	33	96.83%
21	1.3150	34	96.83%
11	1.0280	35	N/A

** cells highlighted in light blue are below detection threshold limit*

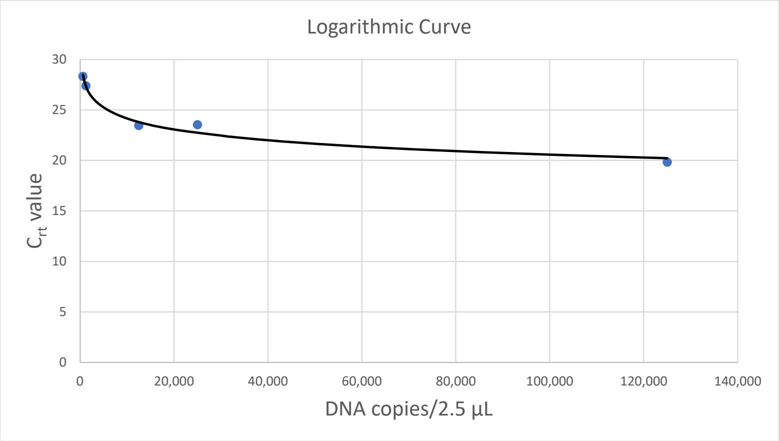

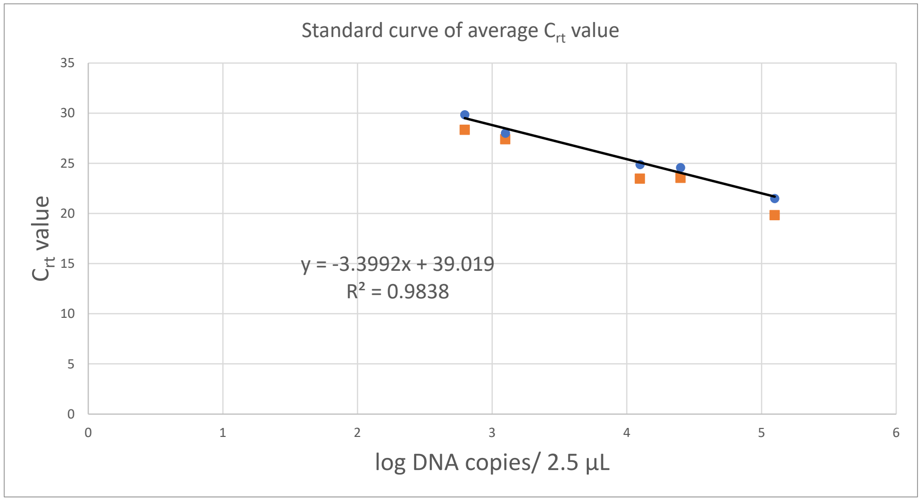

S. lugdunensis	1st C_{rt} Value	2nd C_{rt} Value	3rd C_{rt} Value	Average C_{rt} Value
125,000 copies/2.5 µL (1:1000)	20.374	20.409	20.580	20.454
25,000 copies/2.5 µL (1:5000)	24.091	23.526	23.567	23.728
12,500 copies/2.5 µL (1:10,000)	24.181	23.854	24.152	24.062
1,250 copies/2.5 µL (1:100,000)	27.804	27.757	27.210	27.590
625 copies/2.5 µL (1:200,000)	28.461	29.421	28.355	28.746

Logarithmic curve	
DNA copies/2.5 µL	C_{rt} value
125,000	20.454
25,000	23.728
12,500	24.062
1,250	27.590
625	28.746

Standard curve data of average C_{rt} value		
DNA copies/2.5 µL	log DNA copies/ 2.5 µL	C_{rt} value
125,000	5.096910013	20.454
25,000	4.397940009	23.728
12,500	4.096910013	24.062
1,250	3.096910013	27.590
625	2.795880017	28.746

* Using the average Ct-value mean resulted in the same equation.

y=-3.4817x + 38.484			
DNA copies/2.5 µL	log DNA copies/2.5µL (x)	C_{rt} value (y)	PCR Efficiency
5,558,798	6.7450	15	96.87%
2,869,227	6.4578	16	96.87%
1,480,979	6.1705	17	96.87%
764,421	5.8833	18	96.87%
394,563	5.5961	19	96.87%
203,658	5.3089	20	96.87%
105,120	5.0217	21	96.87%
54,259	4.7345	22	96.87%
28,006	4.4473	23	96.87%
14,456	4.1600	24	96.87%
7,461	3.8728	25	96.87%
3,851	3.5856	26	96.87%
1,988	3.2984	27	96.87%
1,026	3.0112	28	96.87%
530	2.7240	29	96.87%
273	2.4367	30	96.87%
141	2.1495	31	96.87%
73	1.8623	32	96.87%
38	1.5751	33	96.87%
19	1.2879	34	96.87%
10	1.0007	35	N/A

** cells highlighted in light blue are below detection threshold limit*

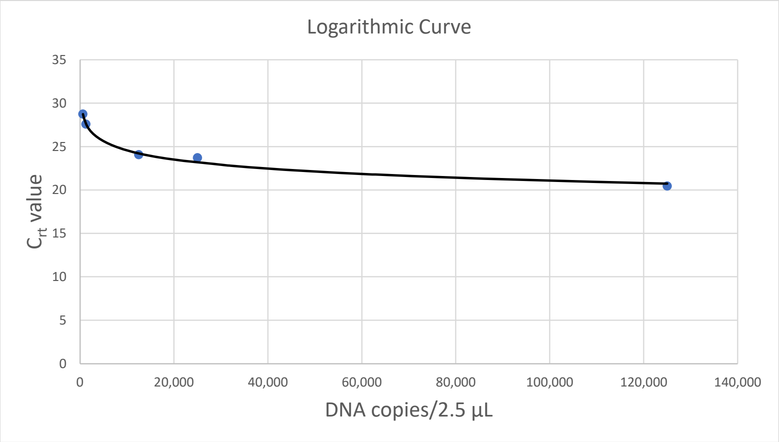

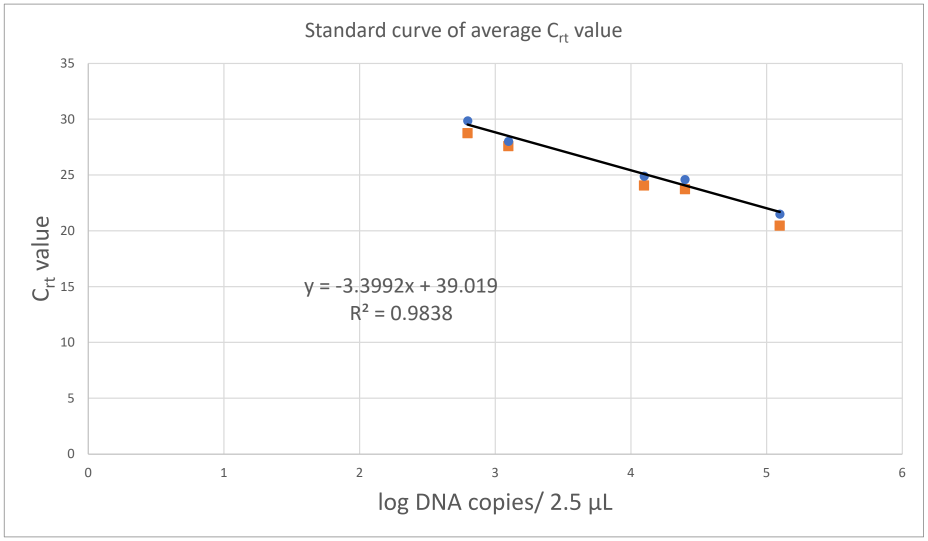

S. haemolyticus	1st C_{rt} Value	2nd C_{rt} Value	3rd C_{rt} Value	Average C_{rt} Value
125,000 copies/2.5 µL (1:1000)	19.903	20.001	19.749	19.884
25,000 copies/2.5 µL (1:5000)	23.590	23.061	22.793	23.148
12,500 copies/2.5 µL (1:10,000)	23.668	23.474	23.539	23.560
1,250 copies/2.5 µL (1:100,000)	27.157	27.137	26.885	27.060
625 copies/2.5 µL (1:200,000)	27.957	29.666	27.921	28.515

Logarithmic curve	
DNA copies/2.5 µL	C_{rt} value
125,000	19.884
25,000	23.148
12,500	23.560
1,250	27.060
625	28.515

Standard curve data of average C_{rt} value		
DNA copies/2.5 µL	log DNA copies/ 2.5 µL	C_{rt} value
125,000	5.096910013	19.884
25,000	4.397940009	23.148
12,500	4.096910013	23.560
1,250	3.096910013	27.060
625	2.795880017	28.515

* Using the average Ct-value mean resulted in the same equation.

y=-3.5922x + 38.432			
DNA copies/2.5 µL	log DNA copies/2.5µL (x)	C_{rt} value (y)	PCR Efficiency
3,334,434	6.5230	15	94.92%
1,756,472	6.2446	16	94.92%
925,252	5.9663	17	94.92%
487,393	5.6879	18	94.92%
256,743	5.4095	19	94.92%
135,244	5.1311	20	94.92%
71,242	4.8527	21	94.92%
37,528	4.5744	22	94.92%
19,769	4.2960	23	94.92%
10,413	4.0176	24	94.92%
5,485	3.7392	25	94.92%
2,890	3.4608	26	94.92%
1,522	3.1825	27	94.92%
802	2.9041	28	94.92%
422	2.6257	29	94.92%
222	2.3473	30	94.92%
117	2.0689	31	94.92%
62	1.7905	32	94.92%
33	1.5122	33	94.92%
17	1.2338	34	94.92%
9	0.9554	35	N/A

** cells highlighted in light blue are below detection threshold limit*

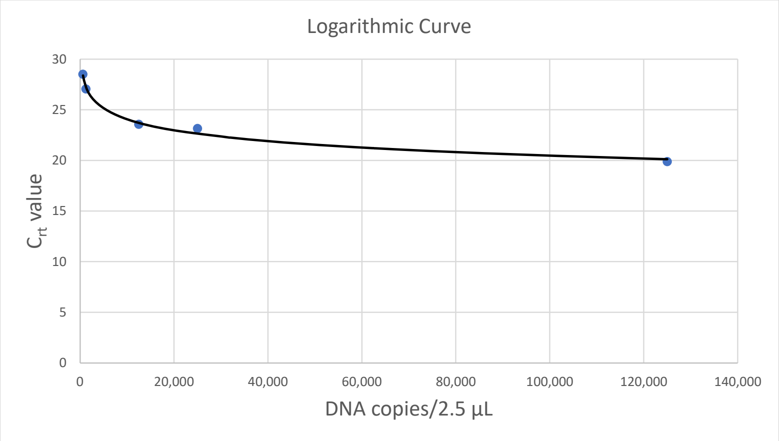

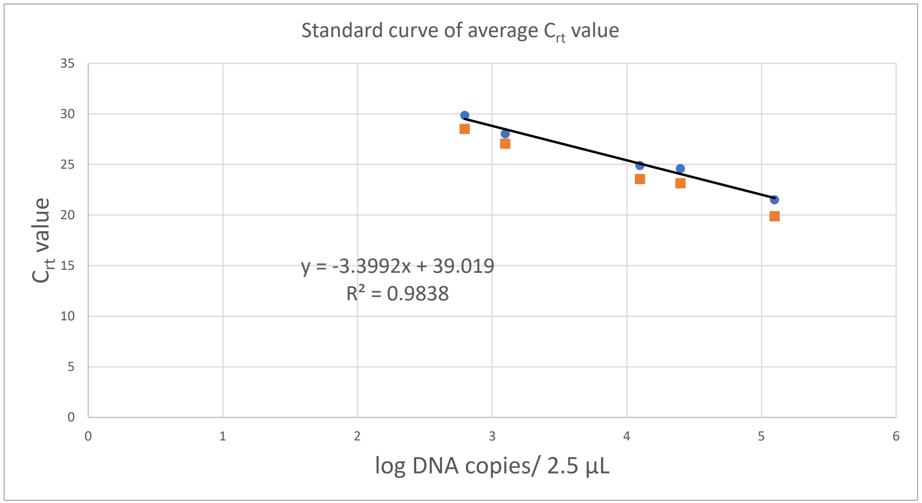

S. marcescens	1st C_{rt} Value	2nd C_{rt} Value	3rd C_{rt} Value	Average C_{rt} Value
125,000 copies/2.5 µL (1:1000)	20.051	20.261	20.268	20.193
25,000 copies/2.5 µL (1:5000)	23.555	23.698	22.859	23.371
12,500 copies/2.5 µL (1:10,000)	23.689	23.629	23.704	23.674
1,250 copies/2.5 µL (1:100,000)	27.673	27.913	27.355	27.647
625 copies/2.5 µL (1:200,000)	28.096	28.761	27.047	27.968

Logarithmic curve	
DNA copies/2.5 µL	C_{rt} value
125,000	20.193
25,000	23.371
12,500	23.674
1,250	27.647
625	27.968

Standard curve data of average C_{rt} value		
DNA copies/2.5 µL	log DNA copies/ 2.5 µL	C_{rt} value
125,000	5.096910013	20.193
25,000	4.397940009	23.371
12,500	4.096910013	23.674
1,250	3.096910013	27.647
625	2.795880017	27.968

* Using the average Ct-value mean resulted in the same equation.

y=-3.4144x + 38.876			
DNA copies/2.5 µL	log DNA copies/2.5µL (x)	C_{rt} value (y)	PCR Efficiency
9,834,146	6.9927	15	98.14%
5,010,250	6.6999	16	98.14%
2,552,597	6.4070	17	98.14%
1,300,484	6.1141	18	98.14%
662,564	5.8212	19	98.14%
337,560	5.5284	20	98.14%
171,978	5.2355	21	98.14%
87,619	4.9426	22	98.14%
44,639	4.6497	23	98.14%
22,743	4.3568	24	98.14%
11,587	4.0640	25	98.14%
5,903	3.7711	26	98.14%
3,008	3.4782	27	98.14%
1,532	3.1853	28	98.14%
781	2.8925	29	98.14%
398	2.5996	30	98.14%
203	2.3067	31	98.14%
103	2.0138	32	98.14%
53	1.7209	33	98.14%
27	1.4281	34	98.14%
14	1.1352	35	N/A

** cells highlighted in light blue are below detection threshold limit*

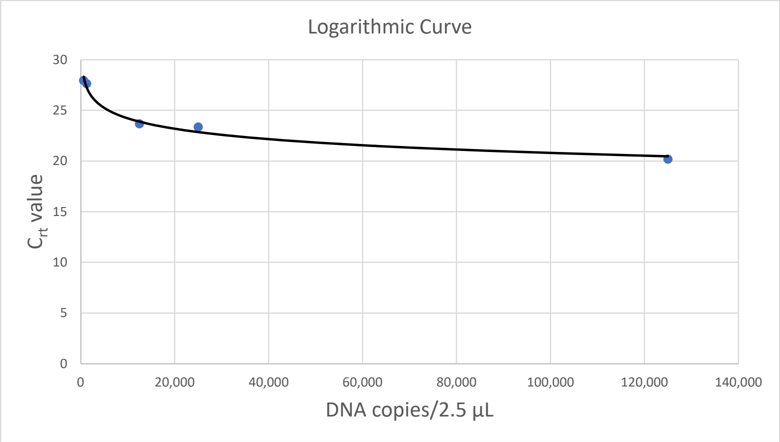

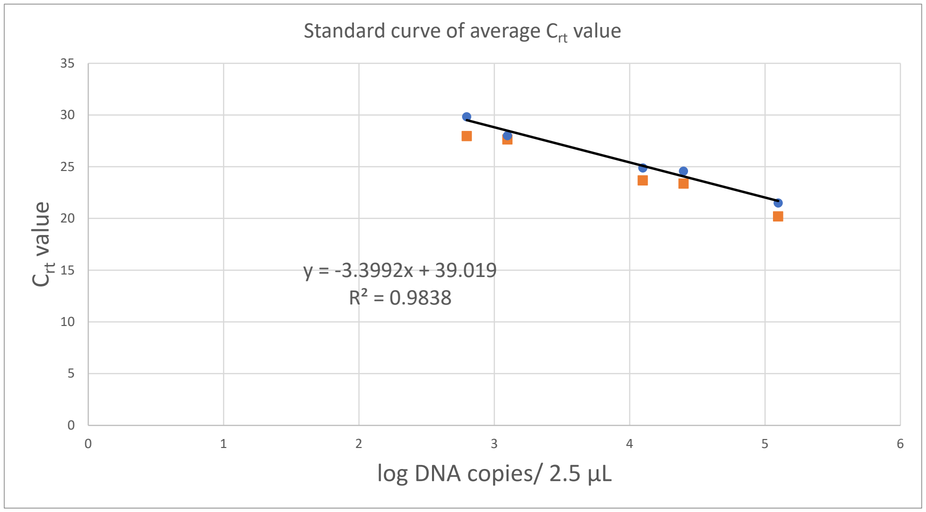

C. amalonaticus	1st C_{rt} Value	2nd C_{rt} Value	3rd C_{rt} Value	Average C_{rt} Value
125,000 copies/2.5 µL (1:1000)	19.621	19.692	19.737	19.683
25,000 copies/2.5 µL (1:5000)	23.213	22.946	22.570	22.909
12,500 copies/2.5 µL (1:10,000)	23.065	23.250	23.528	23.281
1,250 copies/2.5 µL (1:100,000)	26.713	27.019	26.973	26.902
625 copies/2.5 µL (1:200,000)	28.951	27.874	29.062	28.629

Logarithmic curve	
DNA copies/2.5 µL	C_{rt} value
125,000	19.683
25,000	22.909
12,500	23.281
1,250	26.902
625	28.629

Standard curve data of average C_{rt} value		
DNA copies/2.5 µL	log DNA copies/ 2.5 µL	C_{rt} value
125,000	5.096910013	19.683
25,000	4.397940009	22.909
12,500	4.096910013	23.281
1,250	3.096910013	26.902
625	2.795880017	28.629

* Using the average Ct-value mean resulted in the same equation.

y=-3.7084x + 38.732			
DNA copies/2.5 µL	log DNA copies/2.5µL (x)	C_{rt} value (y)	PCR Efficiency
2,509,143	6.3995	15	93.03%
1,348,551	6.1299	16	93.03%
724,785	5.8602	17	93.03%
389,539	5.5906	18	93.03%
209,360	5.3209	19	93.03%
112,521	5.0512	20	93.03%
60,475	4.7816	21	93.03%
32,503	4.5119	22	93.03%
17,469	4.2423	23	93.03%
9,389	3.9726	24	93.03%
5,046	3.7029	25	93.03%
2,712	3.4333	26	93.03%
1,458	3.1636	27	93.03%
783	2.8940	28	93.03%
421	2.6243	29	93.03%
226	2.3547	30	93.03%
122	2.0850	31	93.03%
65	1.8153	32	93.03%
35	1.5457	33	93.03%
19	1.2760	34	93.03%
10	1.0064	35	N/A

** cells highlighted in light blue are below detection threshold limit*

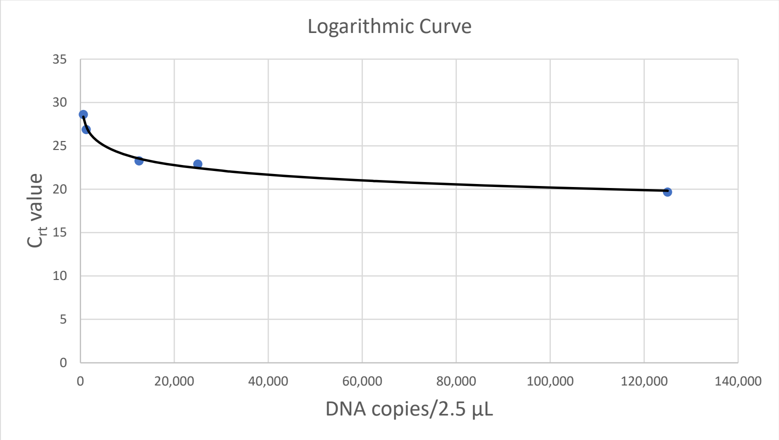

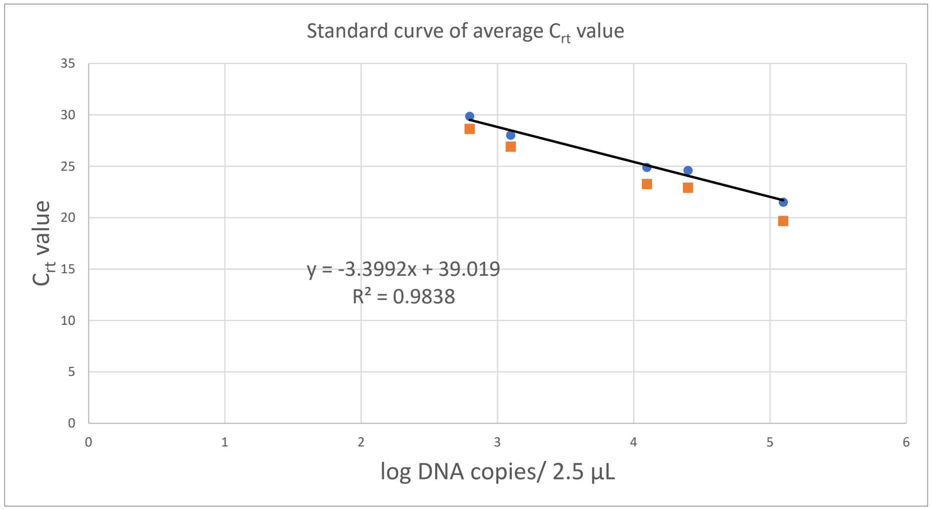

S. oralis	1st C_{rt} Value	2nd C_{rt} Value	3rd C_{rt} Value	Average C_{rt} Value
125,000 copies/2.5 µL (1:1000)	19.769	19.831	19.906	19.835
25,000 copies/2.5 µL (1:5000)	23.766	23.637	23.473	23.625
12,500 copies/2.5 µL (1:10,000)	23.788	23.797	23.941	23.842
1,250 copies/2.5 µL (1:100,000)	27.005	26.280	26.805	26.697
625 copies/2.5 µL (1:200,000)	28.604		28.591	28.598

Logarithmic curve	
DNA copies/2.5 µL	C_{rt} value
125,000	19.835
25,000	23.625
12,500	23.842
1,250	26.697
625	28.598

Standard curve data of average C_{rt} value		
DNA copies/2.5 µL	log DNA copies/ 2.5 µL	C_{rt} value
125,000	5.096910013	19.835
25,000	4.397940009	23.625
12,500	4.096910013	23.842
1,250	3.096910013	26.697
625	2.795880017	28.598

* Using the average Ct-value mean resulted in the same equation.

y=-3.4706x + 38.044			
DNA copies/2.5 µL	log DNA copies/2.5µL (x)	C_{rt} value (y)	PCR Efficiency
4,362,888	6.6398	15	97.07%
2,247,188	6.3516	16	97.07%
1,157,457	6.0635	17	97.07%
596,170	5.7754	18	97.07%
307,069	5.4872	19	97.07%
158,162	5.1991	20	97.07%
81,464	4.9110	21	97.07%
41,960	4.6228	22	97.07%
21,612	4.3347	23	97.07%
11,132	4.0466	24	97.07%
5,734	3.7584	25	97.07%
2,953	3.4703	26	97.07%
1,521	3.1822	27	97.07%
783	2.8940	28	97.07%
404	2.6059	29	97.07%
208	2.3178	30	97.07%
107	2.0296	31	97.07%
55	1.7415	32	97.07%
28	1.4534	33	97.07%
15	1.1652	34	97.07%
8	0.8771	35	N/A

** cells highlighted in light blue are below detection threshold limit*

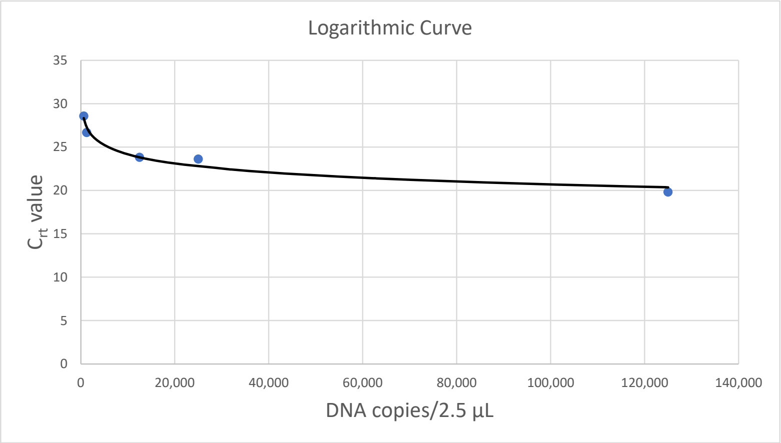

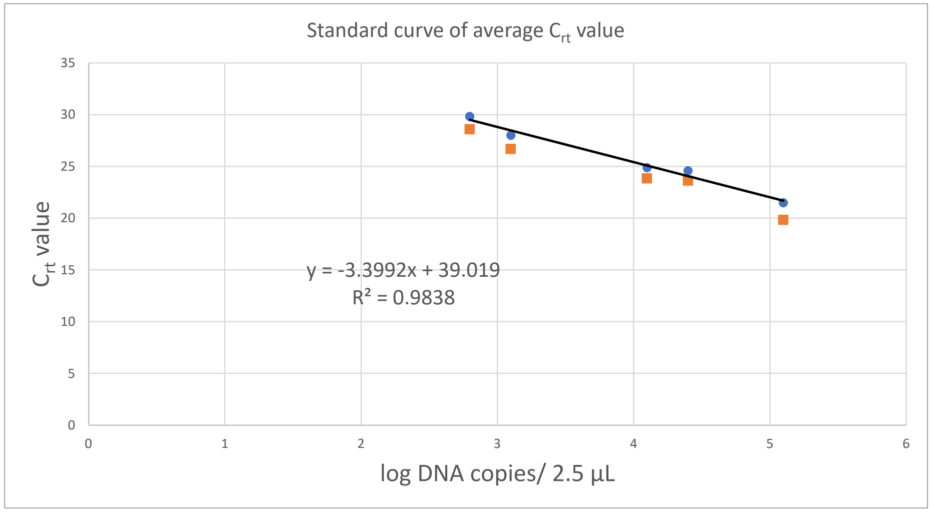

C. glabrata	1st C_{rt} Value	2nd C_{rt} Value	3rd C_{rt} Value	Average C_{rt} Value
125,000 copies/2.5 µL (1:1000)	20.910	20.983	21.194	21.029
25,000 copies/2.5 µL (1:5000)	24.323	24.121	24.017	24.154
12,500 copies/2.5 µL (1:10,000)	24.287	24.510	24.646	24.481
1,250 copies/2.5 µL (1:100,000)	27.766	29.017	27.873	28.219
625 copies/2.5 µL (1:200,000)	29.438	29.029	29.946	29.471

Logarithmic curve	
DNA copies/2.5 µL	C_{rt} value
125,000	21.029
25,000	24.154
12,500	24.481
1,250	28.219
625	29.471

Standard curve data of average C_{rt} value		
DNA copies/2.5 µL	log DNA copies/ 2.5 µL	C_{rt} value
125,000	5.096910013	21.029
25,000	4.397940009	24.154
12,500	4.096910013	24.481
1,250	3.096910013	28.219
625	2.795880017	29.471

* Using the average Ct-value mean resulted in the same equation.

y=-3.5695x + 39.381			
DNA copies/2.5 µL	log DNA copies/2.5µL (x)	C_{rt} value (y)	PCR Efficiency
6,766,567	6.8304	15	95.31%
3,549,908	6.5502	16	95.31%
1,862,369	6.2701	17	95.31%
977,045	5.9899	18	95.31%
512,582	5.7098	19	95.31%
268,913	5.4296	20	95.31%
141,078	5.1495	21	95.31%
74,013	4.8693	22	95.31%
38,829	4.5892	23	95.31%
20,371	4.3090	24	95.31%
10,687	4.0289	25	95.31%
5,607	3.7487	26	95.31%
2,941	3.4686	27	95.31%
1,543	3.1884	28	95.31%
810	2.9083	29	95.31%
425	2.6281	30	95.31%
223	2.3479	31	95.31%
117	2.0678	32	95.31%
61	1.7876	33	95.31%
32	1.5075	34	95.31%
17	1.2273	35	N/A

** cells highlighted in light blue are below detection threshold limit*

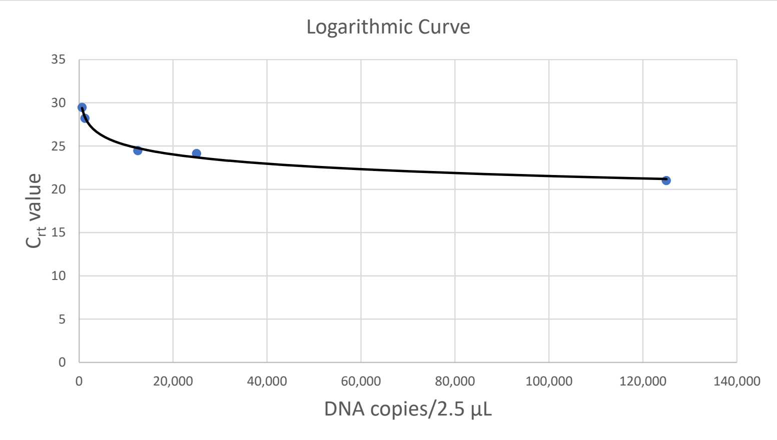

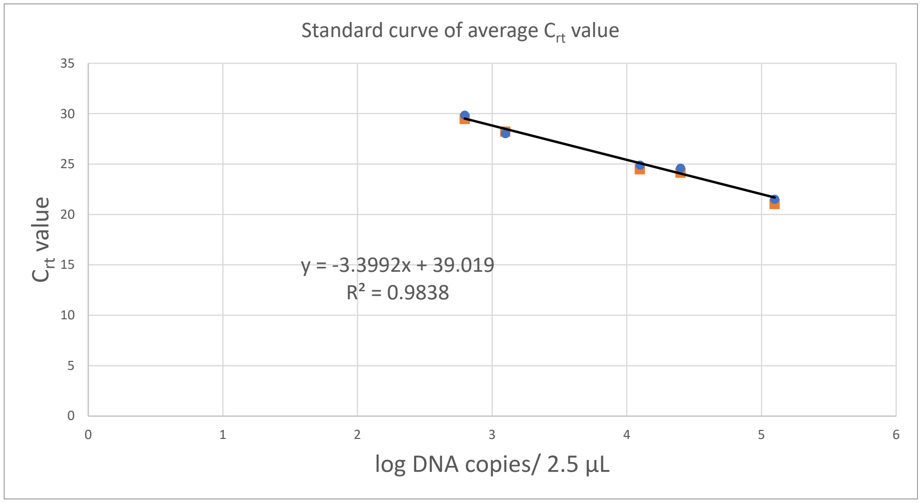

M. tuberculosis	1st C_{rt} Value	2nd C_{rt} Value	3rd C_{rt} Value	Average C_{rt} Value
125,000 copies/2.5 µL (1:1000)	19.475	19.620	19.718	19.604
25,000 copies/2.5 µL (1:5000)	22.667	23.060	22.557	22.761
12,500 copies/2.5 µL (1:10,000)	23.142	23.204	23.421	23.256
1,250 copies/2.5 µL (1:100,000)	26.751	26.684	26.216	26.550
625 copies/2.5 µL (1:200,000)	27.970	28.442	26.787	27.733

Logarithmic curve	
DNA copies/2.5 µL	C_{rt} value
125,000	19.604
25,000	22.761
12,500	23.256
1,250	26.550
625	27.733

Standard curve data of average C_{rt} value		
DNA copies/2.5 µL	log DNA copies/ 2.5 µL	C_{rt} value
125,000	5.096910013	19.604
25,000	4.397940009	22.761
12,500	4.096910013	23.256
1,250	3.096910013	26.550
625	2.795880017	27.733

* Using the average Ct-value mean resulted in the same equation.

y=-3.4032x + 37.243			
DNA copies/2.5 µL	log DNA copies/2.5µL (x)	C_{rt} value (y)	PCR Efficiency
3,434,847	6.5359	15	98.36%
1,746,089	6.2421	16	98.36%
887,616	5.9482	17	98.36%
451,216	5.6544	18	98.36%
229,373	5.3605	19	98.36%
116,601	5.0667	20	98.36%
59,274	4.7729	21	98.36%
30,131	4.4790	22	98.36%
15,317	4.1852	23	98.36%
7,786	3.8913	24	98.36%
3,958	3.5975	25	98.36%
2,012	3.3037	26	98.36%
1,023	3.0098	27	98.36%
520	2.7160	28	98.36%
264	2.4221	29	98.36%
134	2.1283	30	98.36%
68	1.8344	31	98.36%
35	1.5406	32	98.36%
18	1.2468	33	98.36%
9	0.9529	34	98.36%
5	0.6591	35	N/A

** cells highlighted in light blue are below detection threshold limit*

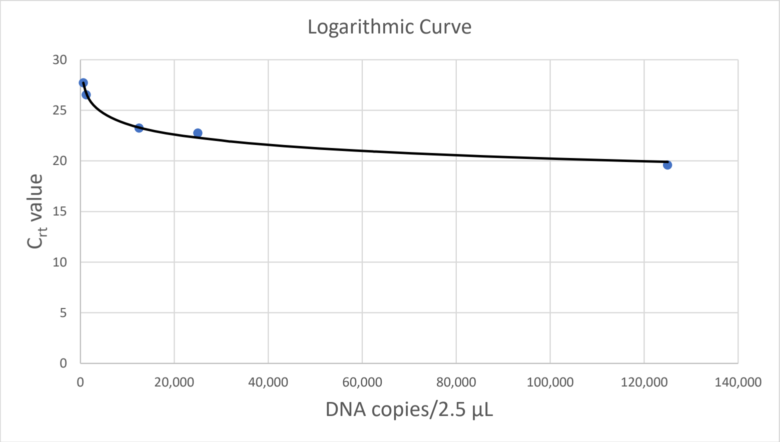

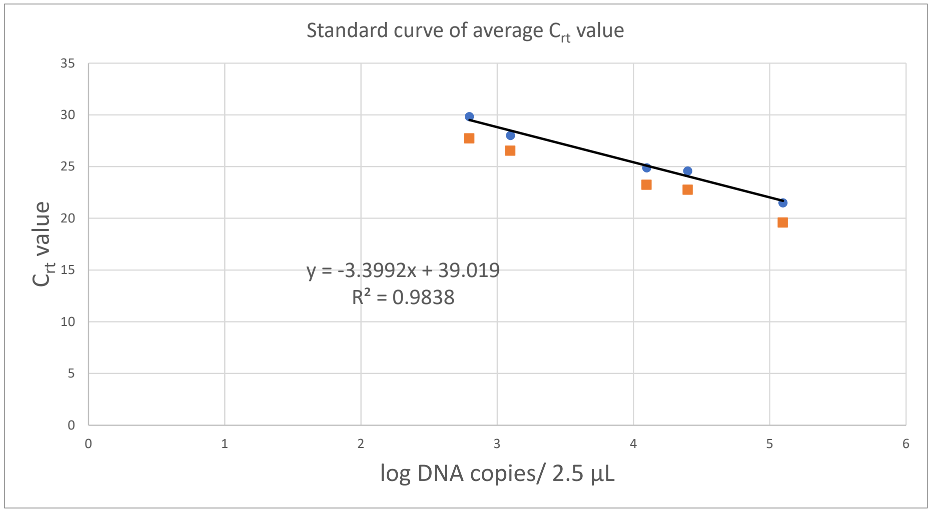

M. genitalium	1st C_{rt} Value	2nd C_{rt} Value	3rd C_{rt} Value	Average C_{rt} Value
125,000 copies/2.5 µL (1:1000)	20.286	20.369	20.447	20.367
25,000 copies/2.5 µL (1:5000)	23.699	23.514	23.008	23.407
12,500 copies/2.5 µL (1:10,000)	23.868	23.918	23.806	23.864
1,250 copies/2.5 µL (1:100,000)	26.678	28.070	28.115	27.621
625 copies/2.5 µL (1:200,000)	29.937	28.402	28.951	29.097

Logarithmic curve	
DNA copies/2.5 µL	C_{rt} value
125,000	20.367
25,000	23.407
12,500	23.864
1,250	27.621
625	29.097

Standard curve data of average C_{rt} value		
DNA copies/2.5 µL	log DNA copies/ 2.5 µL	C_{rt} value
125,000	5.096910013	20.367
25,000	4.397940009	23.407
12,500	4.096910013	23.864
1,250	3.096910013	27.621
625	2.795880017	29.097

y=-3.6815x + 39.218			
DNA copies/2.5 µL	log DNA copies/2.5µL (x)	C_{rt} value (y)	PCR Efficiency
3,787,014	6.5783	15	93.45%
2,026,135	6.3067	16	93.45%
1,084,027	6.0350	17	93.45%
579,978	5.7634	18	93.45%
310,301	5.4918	19	93.45%
166,018	5.2202	20	93.45%
88,823	4.9485	21	93.45%
47,522	4.6769	22	93.45%
25,426	4.4053	23	93.45%
13,603	4.1336	24	93.45%
7,278	3.8620	25	93.45%
3,894	3.5904	26	93.45%
2,083	3.3188	27	93.45%
1,115	3.0471	28	93.45%
596	2.7755	29	93.45%
319	2.5039	30	93.45%
171	2.2322	31	93.45%
91	1.9606	32	93.45%
49	1.6890	33	93.45%
26	1.4174	34	93.45%
14	1.1457	35	N/A

** cells highlighted in light blue are below detection threshold limit*

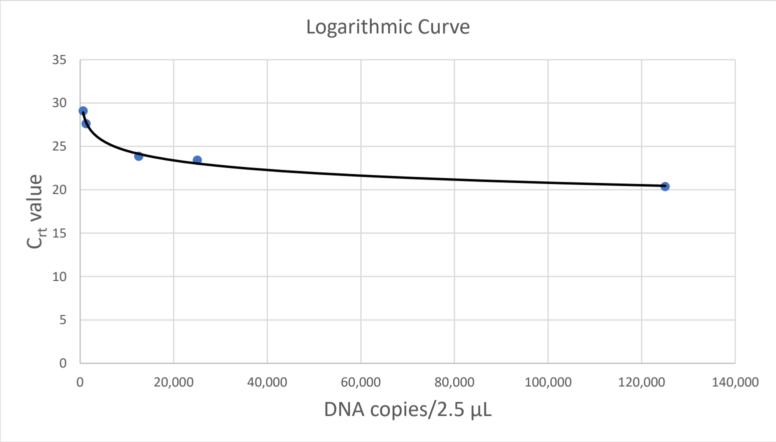

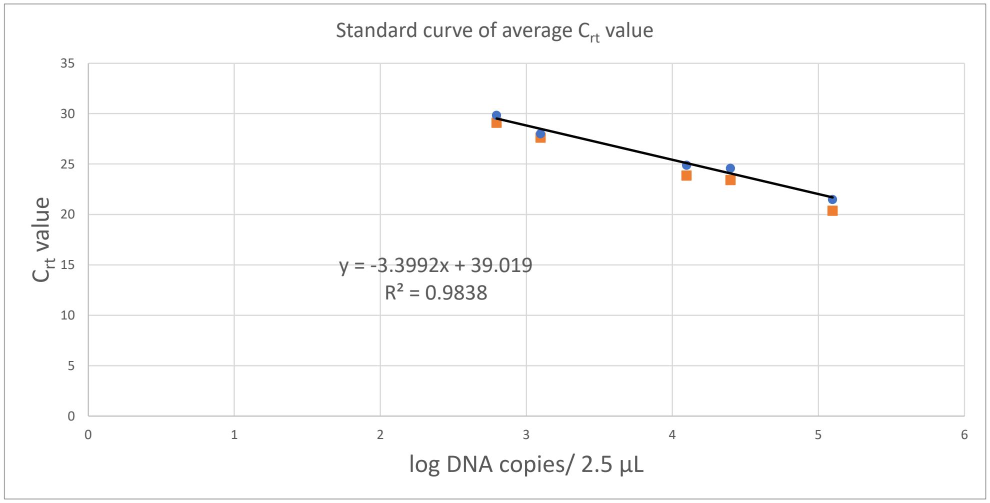

M. hominis	1st C_{rt} Value	2nd C_{rt} Value	3rd C_{rt} Value	Average C_{rt} Value
125,000 copies/2.5 µL (1:1000)	19.361	19.484	19.613	19.486
25,000 copies/2.5 µL (1:5000)	22.947	22.574	22.482	22.668
12,500 copies/2.5 µL (1:10,000)	22.808	23.315	23.080	23.068
1,250 copies/2.5 µL (1:100,000)	27.387	27.088	27.396	27.290
625 copies/2.5 µL (1:200,000)	26.596	28.296	28.047	27.646

Logarithmic curve	
DNA copies/2.5 µL	C_{rt} value
125,000	19.486
25,000	22.668
12,500	23.068
1,250	27.290
625	27.646

Standard curve data of average C_{rt} value		
DNA copies/2.5 µL	log DNA copies/ 2.5 µL	C_{rt} value
125,000	5.096910013	19.486
25,000	4.397940009	22.668
12,500	4.096910013	23.068
1,250	3.096910013	27.290
625	2.795880017	27.646

* Using the average Ct-value mean resulted in the same equation.

y=-3.605x + 38.08			
DNA copies/2.5 µL	log DNA copies/2.5µL (x)	C_{rt} value (y)	PCR Efficiency
2,524,754	6.4022	15	94.70%
1,332,989	6.1248	16	94.70%
703,775	5.8474	17	94.70%
371,571	5.5700	18	94.70%
196,177	5.2926	19	94.70%
103,575	5.0153	20	94.70%
54,684	4.7379	21	94.70%
28,872	4.4605	22	94.70%
15,243	4.1831	23	94.70%
8,048	3.9057	24	94.70%
4,249	3.6283	25	94.70%
2,243	3.3509	26	94.70%
1,184	3.0735	27	94.70%
625	2.7961	28	94.70%
330	2.5187	29	94.70%
174	2.2413	30	94.70%
92	1.9639	31	94.70%
49	1.6865	32	94.70%
26	1.4092	33	94.70%
14	1.1318	34	94.70%
7	0.8544	35	N/A

** cells highlighted in light blue are below detection threshold limit*

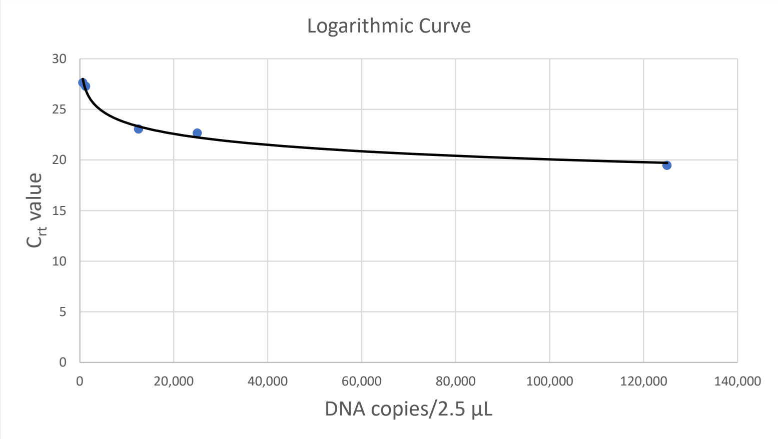

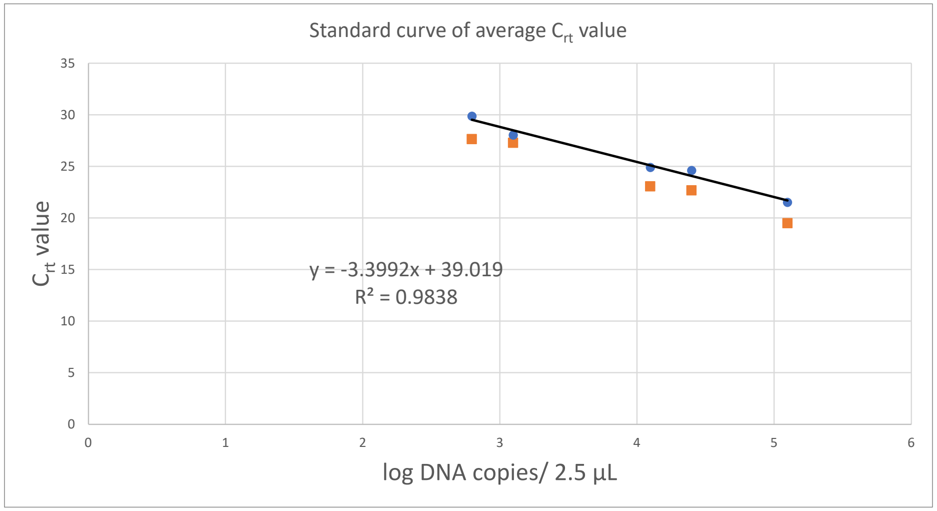

P. agglomerans	1st C_{rt} Value	2nd C_{rt} Value	3rd C_{rt} Value	Average C_{rt} Value
125,000 copies/2.5 µL (1:1000)	20.741	20.764	20.879	20.795
25,000 copies/2.5 µL (1:5000)	24.405	23.908	23.951	24.088
12,500 copies/2.5 µL (1:10,000)	24.259	24.203	24.529	24.330
1,250 copies/2.5 µL (1:100,000)	27.244	27.691	28.781	27.905
625 copies/2.5 µL (1:200,000)	29.928	28.809	28.919	29.219

Logarithmic curve	
DNA copies/2.5 µL	C_{rt} value
125,000	20.795
25,000	24.088
12,500	24.330
1,250	27.905
625	29.219

Standard curve data of average C_{rt} value		
DNA copies/2.5 µL	log DNA copies/ 2.5 µL	C_{rt} value
125,000	5.096910013	20.795
25,000	4.397940009	24.088
12,500	4.096910013	24.330
1,250	3.096910013	27.905
625	2.795880017	29.219

* Using the average Ct-value mean resulted in the same equation.

y=-3.5181x + 38.977			
DNA copies/2.5 µL	log DNA copies/2.5µL (x)	C_{rt} value (y)	PCR Efficiency
6,536,217	6.8153	15	96.21%
3,396,895	6.5311	16	96.21%
1,765,378	6.2468	17	96.21%
917,473	5.9626	18	96.21%
476,814	5.6783	19	96.21%
247,802	5.3941	20	96.21%
128,784	5.1099	21	96.21%
66,929	4.8256	22	96.21%
34,783	4.5414	23	96.21%
18,077	4.2571	24	96.21%
9,395	3.9729	25	96.21%
4,882	3.6886	26	96.21%
2,537	3.4044	27	96.21%
1,319	3.1202	28	96.21%
685	2.8359	29	96.21%
356	2.5517	30	96.21%
185	2.2674	31	96.21%
96	1.9832	32	96.21%
50	1.6989	33	96.21%
26	1.4147	34	96.21%
14	1.1304	35	N/A

** cells highlighted in light blue are below detection threshold limit*

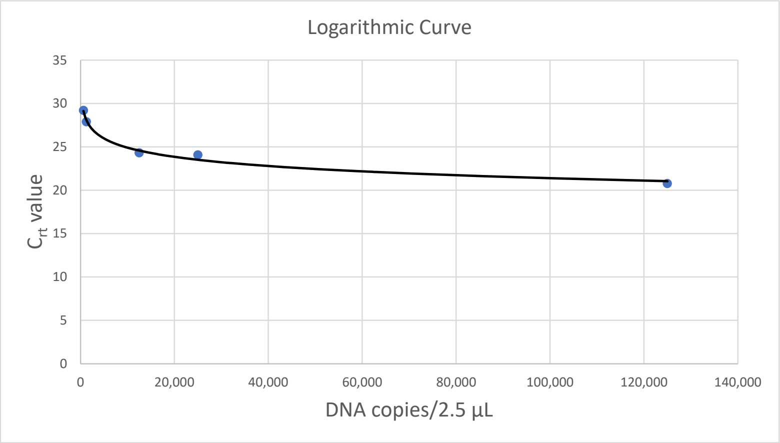

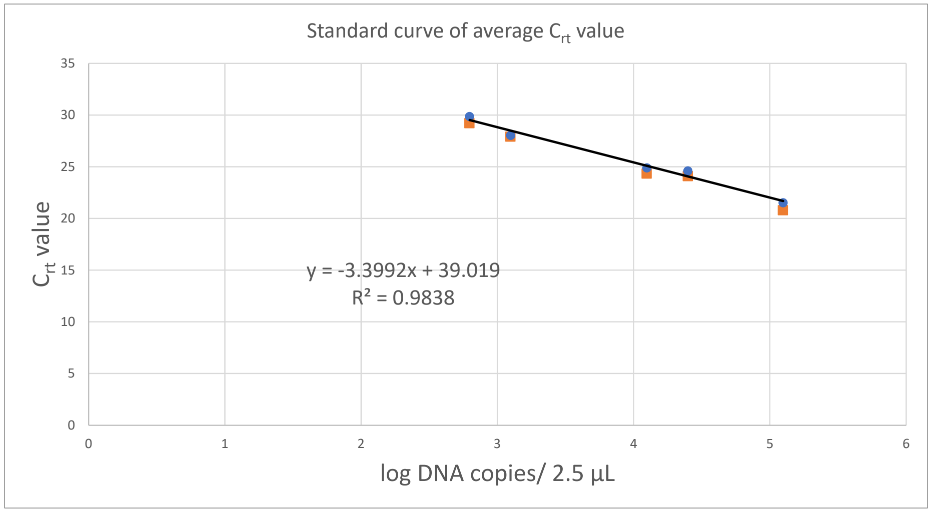

U. urealyticum	1st C_{rt} Value	2nd C_{rt} Value	3rd C_{rt} Value	Average C_{rt} Value
125,000 copies/2.5 µL (1:1000)	19.612	19.695	19.701	19.669
25,000 copies/2.5 µL (1:5000)	23.219	22.486	22.679	22.795
12,500 copies/2.5 µL (1:10,000)	22.873	23.390	23.035	23.099
1,250 copies/2.5 µL (1:100,000)	26.779	26.770	26.647	26.732
625 copies/2.5 µL (1:200,000)	27.559	26.769	27.473	27.267

Logarithmic curve	
DNA copies/2.5 µL	C_{rt} value
125,000	19.669
25,000	22.795
12,500	23.099
1,250	26.732
625	27.267

Standard curve data of average C_{rt} value		
DNA copies/2.5 µL	log DNA copies/ 2.5 µL	C_{rt} value
125,000	5.096910013	19.669
25,000	4.397940009	22.795
12,500	4.096910013	23.099
1,250	3.096910013	26.732
625	2.795880017	27.267

* Using the average Ct-value mean resulted in the same equation.

y=-3.2829x + 36.706			
DNA copies/2.5 µL	log DNA copies/2.5µL (x)	C_{rt} value (y)	PCR Efficiency
4,091,072	6.6118	15	100.00%
2,028,749	6.3072	16	100.00%
1,006,050	6.0026	17	100.00%
498,897	5.6980	18	100.00%
247,401	5.3934	19	100.00%
122,686	5.0888	20	100.00%
60,839	4.7842	21	100.00%
30,170	4.4796	22	100.00%
14,961	4.1750	23	100.00%
7,419	3.8704	24	100.00%
3,679	3.5657	25	100.00%
1,824	3.2611	26	100.00%
905	2.9565	27	100.00%
449	2.6519	28	100.00%
222	2.3473	29	100.00%
110	2.0427	30	100.00%
55	1.7381	31	100.00%
27	1.4335	32	100.00%
13	1.1289	33	100.00%
7	0.8243	34	100.00%
3	0.5197	35	N/A

** cells highlighted in light blue are below detection threshold limit*

*** PCR efficiency is a calculation where the theoretical maximum is 100%. If the equation has calculated a PCR efficiency over 100%, the maximum value of 100% is represented.*

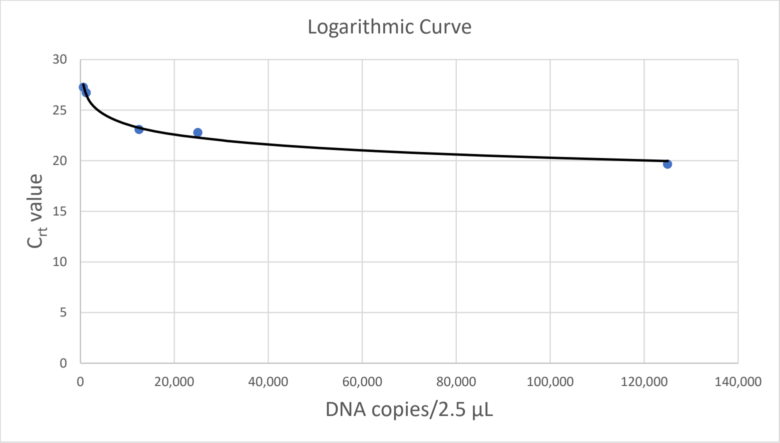

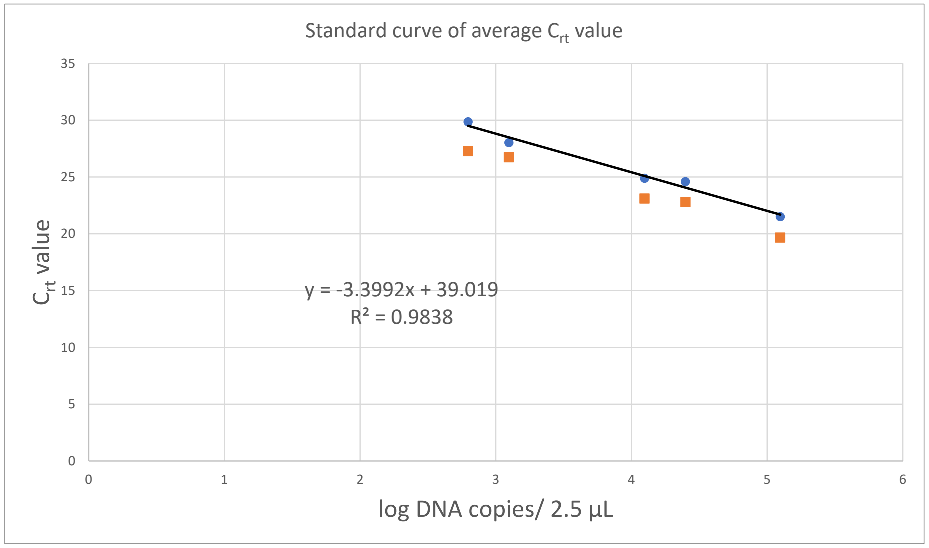

CMV	1st C_{rt} Value	2nd C_{rt} Value	3rd C_{rt} Value	Average C_{rt} Value
125,000 copies/2.5 µL (1:1000)	20.179	20.255	20.481	20.305
25,000 copies/2.5 µL (1:5000)	23.906	23.623	23.874	23.801
12,500 copies/2.5 µL (1:10,000)	23.669	24.013	23.822	23.835
1,250 copies/2.5 µL (1:100,000)	27.559	27.619	28.533	27.904
625 copies/2.5 µL (1:200,000)	27.836	28.144	28.179	28.053

Logarithmic curve	
DNA copies/2.5 µL	C_{rt} value
125,000	20.305
25,000	23.801
12,500	23.835
1,250	27.904
625	28.053

Standard curve data of average C_{rt} value		
DNA copies/2.5 µL	log DNA copies/ 2.5 µL	C_{rt} value
125,000	5.096910013	20.305
25,000	4.397940009	23.801
12,500	4.096910013	23.835
1,250	3.096910013	27.904
625	2.795880017	28.053

* Using the average Ct-value mean resulted in the same equation.

y=-3.3914x + 37.995			
DNA copies/2.5 µL	log DNA copies/2.5µL (x)	C_{rt} value (y)	PCR Efficiency
6,030,949	6.7804	15	98.59%
3,058,597	6.4855	16	98.59%
1,551,168	6.1907	17	98.59%
786,675	5.8958	18	98.59%
398,962	5.6009	19	98.59%
202,334	5.3061	20	98.59%
102,614	5.0112	21	98.59%
52,040	4.7163	22	98.59%
26,392	4.4215	23	98.59%
13,385	4.1266	24	98.59%
6,788	3.8318	25	98.59%
3,443	3.5369	26	98.59%
1,746	3.2420	27	98.59%
885	2.9472	28	98.59%
449	2.6523	29	98.59%
228	2.3574	30	98.59%
115	2.0626	31	98.59%
59	1.7677	32	98.59%
30	1.4728	33	98.59%
15	1.1780	34	98.59%
8	0.8831	35	N/A

** cells highlighted in light blue are below detection threshold limit*

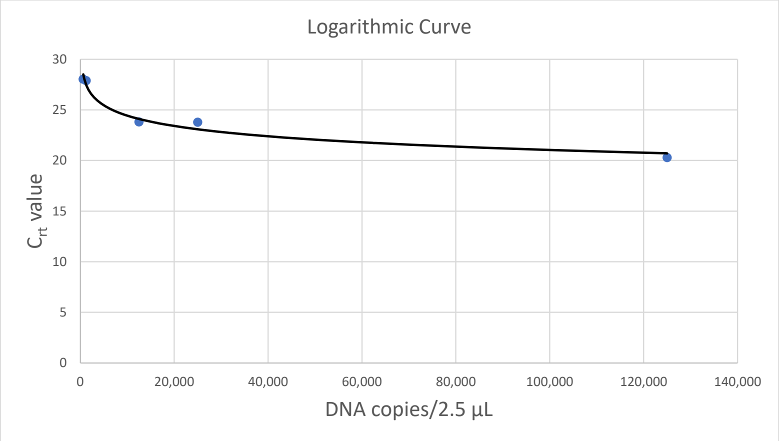

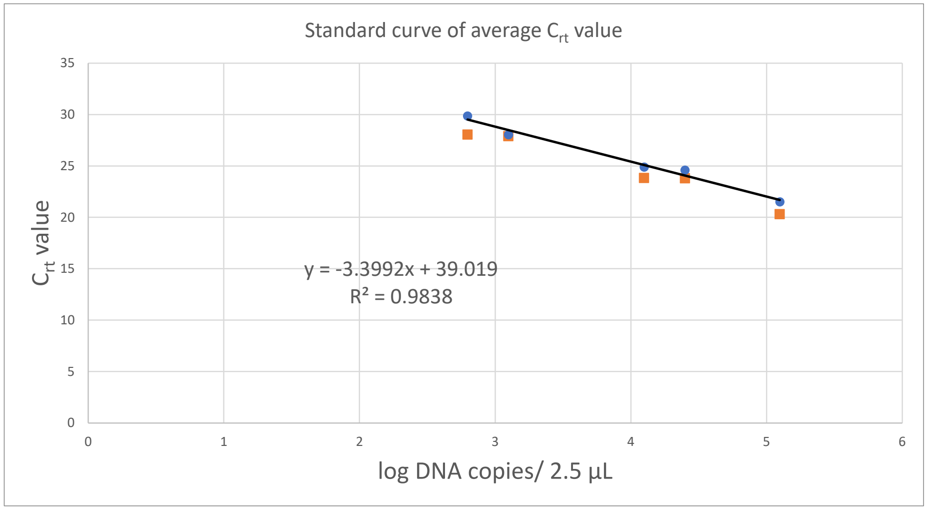

HSV1	1st C_{rt} Value	2nd C_{rt} Value	3rd C_{rt} Value	Average C_{rt} Value
125,000 copies/2.5 µL (1:1000)	21.265	21.238	21.426	21.310
25,000 copies/2.5 µL (1:5000)	24.019	23.894	23.944	23.952
12,500 copies/2.5 µL (1:10,000)	24.919	24.687	24.740	24.782
1,250 copies/2.5 µL (1:100,000)	28.445	27.977	28.497	28.306
625 copies/2.5 µL (1:200,000)	29.143	29.162	28.751	29.019

Logarithmic curve	
DNA copies/2.5 µL	C_{rt} value
125,000	21.310
25,000	23.952
12,500	24.782
1,250	28.306
625	29.019

Standard curve data of average C_{rt} value		
DNA copies/2.5 µL	log DNA copies/ 2.5 µL	C_{rt} value
125,000	5.096910013	21.310
25,000	4.397940009	23.952
12,500	4.096910013	24.782
1,250	3.096910013	28.306
625	2.795880017	29.019

* Using the average Ct-value mean resulted in the same equation.

y=-3.3675x + 38.597			
DNA copies/2.5 µL	log DNA copies/2.5µL (x)	C_{rt} value (y)	PCR Efficiency
10,168,934	7.0073	15	99.07%
5,132,385	6.7103	16	99.07%
2,590,377	6.4134	17	99.07%
1,307,395	6.1164	18	99.07%
659,858	5.8195	19	99.07%
333,038	5.5225	20	99.07%
168,089	5.2255	21	99.07%
84,836	4.9286	22	99.07%
42,818	4.6316	23	99.07%
21,611	4.3347	24	99.07%
10,907	4.0377	25	99.07%
5,505	3.7408	26	99.07%
2,778	3.4438	27	99.07%
1,402	3.1468	28	99.07%
708	2.8499	29	99.07%
357	2.5529	30	99.07%
180	2.2560	31	99.07%
91	1.9590	32	99.07%
46	1.6621	33	99.07%
23	1.3651	34	99.07%
12	1.0682	35	N/A

** cells highlighted in light blue are below detection threshold limit*

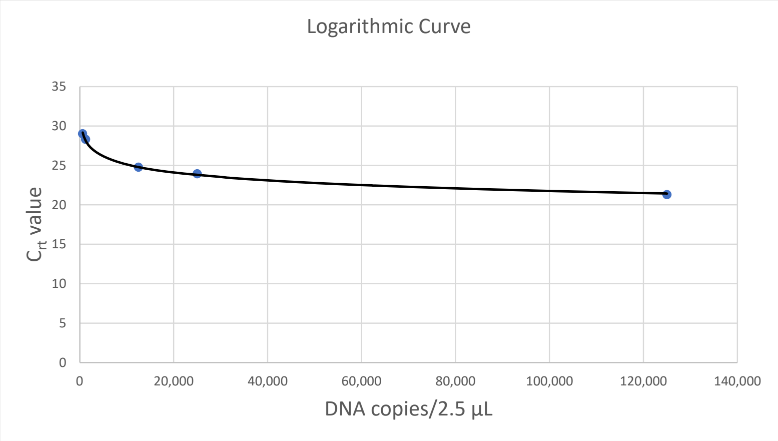

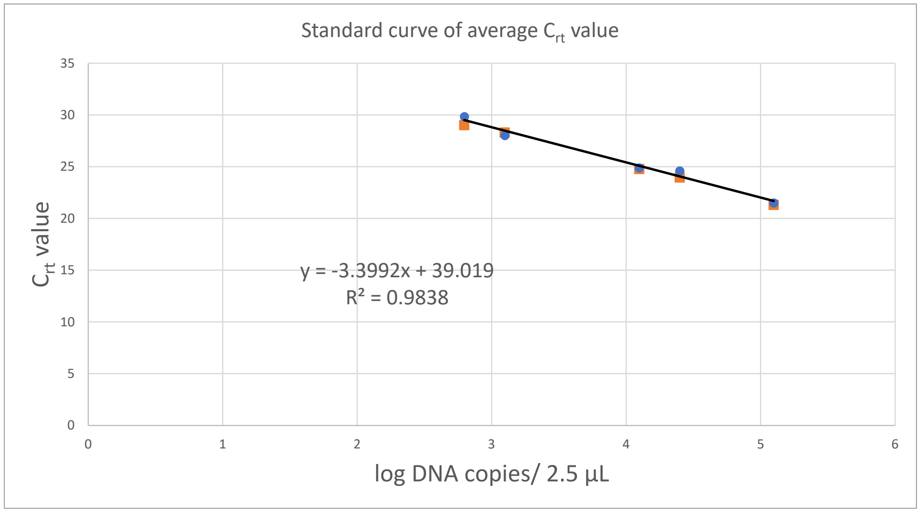

HSV2	1st C_{rt} Value	2nd C_{rt} Value	3rd C_{rt} Value	Average C_{rt} Value
125,000 copies/2.5 µL (1:1000)	19.748	19.812	19.838	19.799
25,000 copies/2.5 µL (1:5000)	23.132	23.012	22.844	22.996
12,500 copies/2.5 µL (1:10,000)	22.984	23.348	23.419	23.250
1,250 copies/2.5 µL (1:100,000)	27.257	27.304	26.235	26.932
625 copies/2.5 µL (1:200,000)	28.162	27.392	27.199	27.584

Logarithmic curve	
DNA copies/2.5 µL	C_{rt} value
125,000	19.799
25,000	22.996
12,500	23.250
1,250	26.932
625	27.584

Standard curve data of average C_{rt} value		
DNA copies/2.5 µL	log DNA copies/ 2.5 µL	C_{rt} value
125,000	5.096910013	19.799
25,000	4.397940009	22.996
12,500	4.096910013	23.250
1,250	3.096910013	26.932
625	2.795880017	27.584

* Using the average Ct-value mean resulted in the same equation.

y=-3.3449x + 37.147			
DNA copies/2.5 µL	log DNA copies/2.5µL (x)	C_{rt} value (y)	PCR Efficiency
4,179,504	6.6211	15	99.53%
2,099,723	6.3222	16	99.53%
1,054,871	6.0232	17	99.53%
529,952	5.7242	18	99.53%
266,241	5.4253	19	99.53%
133,756	5.1263	20	99.53%
67,197	4.8273	21	99.53%
33,759	4.5284	22	99.53%
16,960	4.2294	23	99.53%
8,520	3.9305	24	99.53%
4,281	3.6315	25	99.53%
2,150	3.3325	26	99.53%
1,080	3.0336	27	99.53%
543	2.7346	28	99.53%
273	2.4356	29	99.53%
137	2.1367	30	99.53%
69	1.8377	31	99.53%
35	1.5388	32	99.53%
17	1.2398	33	99.53%
9	0.9408	34	99.53%
4	0.6419	35	N/A

** cells highlighted in light blue are below detection threshold limit*

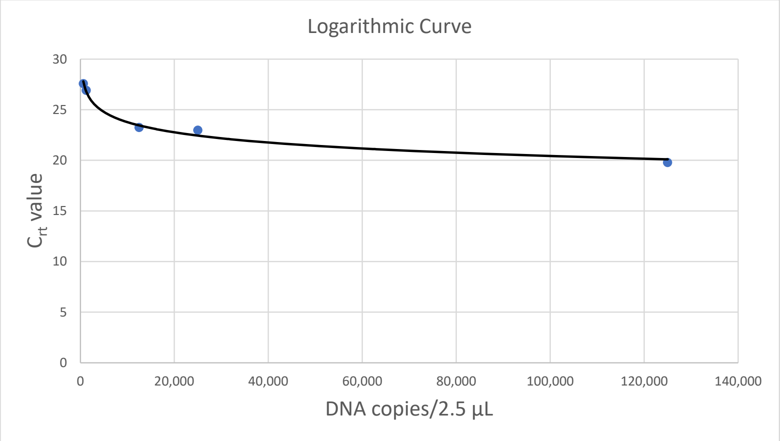

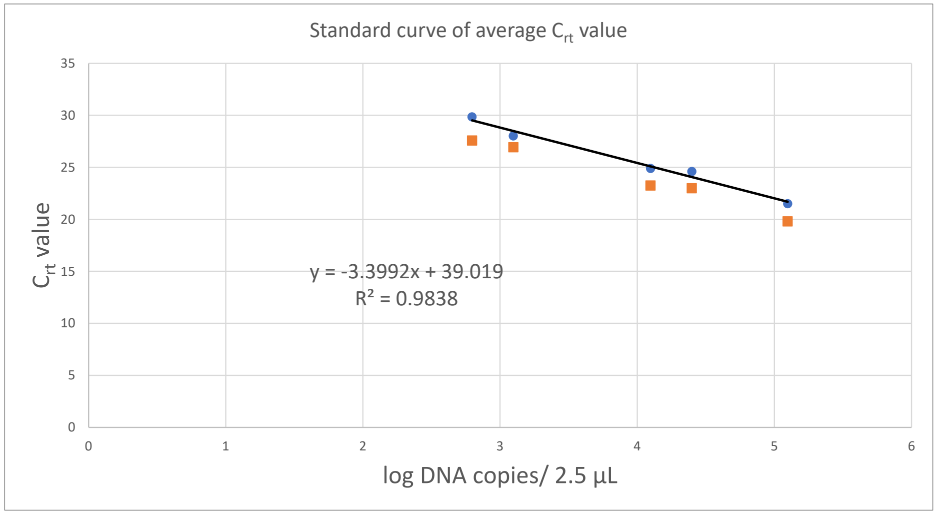

A. schaalii	1st C_{rt} Value	2nd C_{rt} Value	3rd C_{rt} Value	Average C_{rt} Value
125,000 copies/2.5 µL (1:1000)	20.554	20.544	20.574	20.557
25,000 copies/2.5 µL (1:5000)	24.124	23.542	23.405	23.690
12,500 copies/2.5 µL (1:10,000)	24.148	24.314	24.396	24.286
1,250 copies/2.5 µL (1:100,000)	27.933	28.035	28.129	28.032
625 copies/2.5 µL (1:200,000)	28.064	28.178	29.450	28.564

Logarithmic curve	
DNA copies/2.5 µL	C_{rt} value
125,000	20.557
25,000	23.690
12,500	24.286
1,250	28.032
625	28.564

Standard curve data of average C_{rt} value		
DNA copies/2.5 µL	log DNA copies/ 2.5 µL	C_{rt} value
125,000	5.096910013	20.557
25,000	4.397940009	23.690
12,500	4.096910013	24.286
1,250	3.096910013	28.032
625	2.795880017	28.564

* Using the average Ct-value mean resulted in the same equation.

y=-3.34829x + 38.598

DNA copies/2.5 µL	log DNA copies/2.5µL (x)	C_{rt} value (y)	PCR Efficiency
5,961,950	6.7754	15	96.85%
3,078,019	6.4883	16	96.85%
1,589,111	6.2012	17	96.85%
820,422	5.9140	18	96.85%
423,565	5.6269	19	96.85%
218,677	5.3398	20	96.85%
112,898	5.0527	21	96.85%
58,287	4.7656	22	96.85%
30,092	4.4785	23	96.85%
15,536	4.1913	24	96.85%
8,021	3.9042	25	96.85%
4,141	3.6171	26	96.85%
2,138	3.3300	27	96.85%
1,104	3.0429	28	96.85%
570	2.7557	29	96.85%
294	2.4686	30	96.85%
152	2.1815	31	96.85%
78	1.8944	32	96.85%
40	1.6073	33	96.85%
21	1.3202	34	96.85%
11	1.0330	35	N/A

** cells highlighted in light blue are below detection threshold limit*

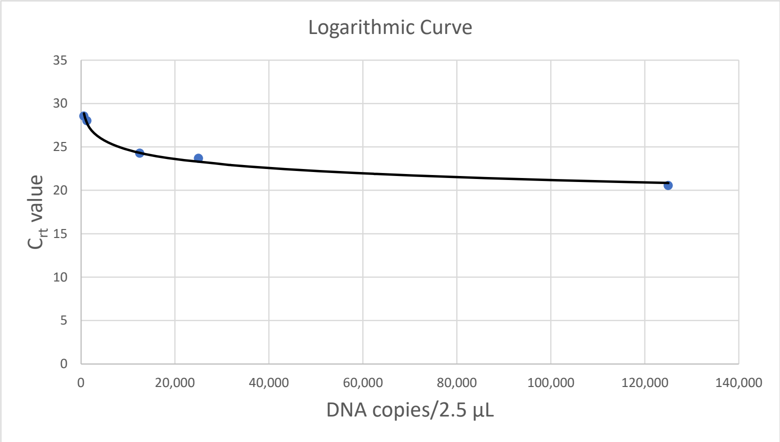

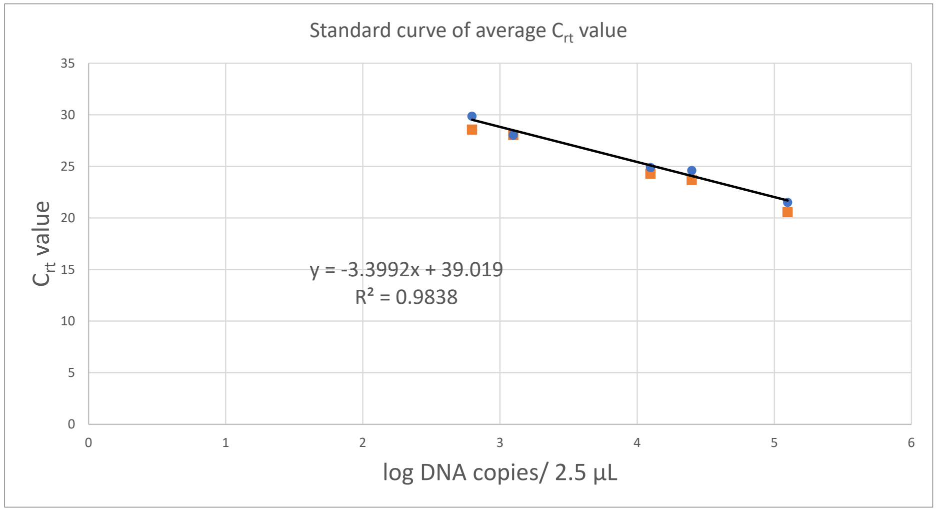

A. urinae	1st C_{rt} Value	2nd C_{rt} Value	3rd C_{rt} Value	Average C_{rt} Value
125,000 copies/2.5 µL (1:1000)	19.803	19.827	19.872	19.834
25,000 copies/2.5 µL (1:5000)	23.370	22.830	23.400	23.200
12,500 copies/2.5 µL (1:10,000)	23.148	23.763	23.571	23.494
1,250 copies/2.5 µL (1:100,000)	26.728	27.224	27.536	27.163
625 copies/2.5 µL (1:200,000)	28.443	27.307	27.001	27.584

Logarithmic curve	
DNA copies/2.5 µL	C_{rt} value
125,000	19.834
25,000	23.200
12,500	23.494
1,250	27.163
625	27.584

Standard curve data of average C_{rt} value		
DNA copies/2.5 µL	log DNA copies/ 2.5 µL	C_{rt} value
125,000	5.096910013	19.834
25,000	4.397940009	23.200
12,500	4.096910013	23.494
1,250	3.096910013	27.163
625	2.795880017	27.584

* Using the average Ct-value mean resulted in the same equation.

y=-3.3424x + 37.28			
DNA copies/2.5 µL	log DNA copies/2.5µL (x)	C_{rt} value (y)	PCR Efficiency
4,633,070	6.6659	15	99.58%
2,326,391	6.3667	16	99.58%
1,168,144	6.0675	17	99.58%
586,557	5.7683	18	99.58%
294,526	5.4691	19	99.58%
147,890	5.1699	20	99.58%
74,259	4.8708	21	99.58%
37,288	4.5716	22	99.58%
18,723	4.2724	23	99.58%
9,401	3.9732	24	99.58%
4,721	3.6740	25	99.58%
2,370	3.3748	26	99.58%
1,190	3.0756	27	99.58%
598	2.7764	28	99.58%
300	2.4773	29	99.58%
151	2.1781	30	99.58%
76	1.8789	31	99.58%
38	1.5797	32	99.58%
19	1.2805	33	99.58%
10	0.9813	34	99.58%
5	0.6821	35	N/A

** cells highlighted in light blue are below detection threshold limit*

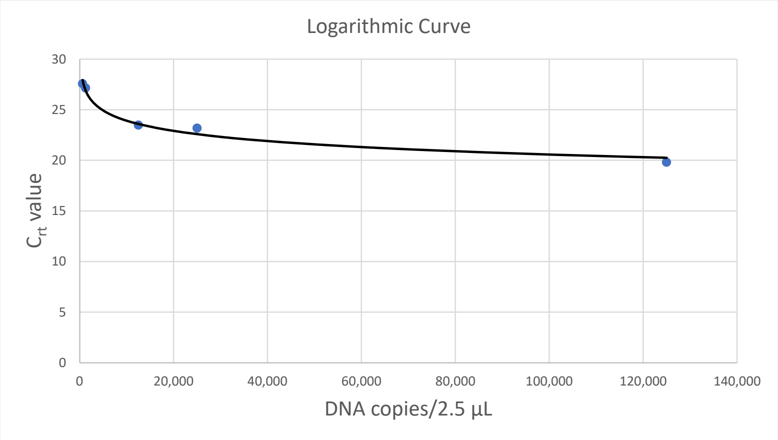

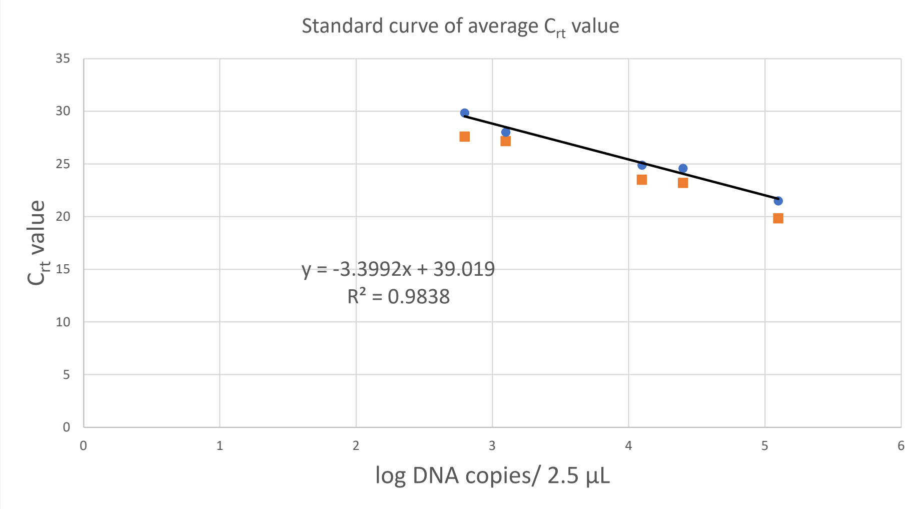

A. ominicolens	1st C_{rt} Value	2nd C_{rt} Value	3rd C_{rt} Value	Average C_{rt} Value
125,000 copies/2.5 µL (1:1000)	20.252	20.178	20.357	20.262
25,000 copies/2.5 µL (1:5000)	23.056	23.052	23.625	23.244
12,500 copies/2.5 µL (1:10,000)	23.807	23.766	23.840	23.804
1,250 copies/2.5 µL (1:100,000)	27.223	26.875	26.889	26.996
625 copies/2.5 µL (1:200,000)	28.316	28.155	28.391	28.287

Logarithmic curve	
DNA copies/2.5 µL	C_{rt} value
125,000	20.262
25,000	23.244
12,500	23.804
1,250	26.996
625	28.287

Standard curve data of average C_{rt} value		
DNA copies/2.5 µL	log DNA copies/ 2.5 µL	C_{rt} value
125,000	5.096910013	20.262
25,000	4.397940009	23.244
12,500	4.096910013	23.804
1,250	3.096910013	26.996
625	2.795880017	28.287

* Using the average Ct-value mean resulted in the same equation.

y=-3.3545x + 37.591			
DNA copies/2.5 µL	log DNA copies/2.5µL (x)	C_{rt} value (y)	PCR Efficiency
5,426,699	6.7345	15	99.33%
2,731,673	6.4364	16	99.33%
1,375,060	6.1383	17	99.33%
692,173	5.8402	18	99.33%
348,424	5.5421	19	99.33%
175,388	5.2440	20	99.33%
88,286	4.9459	21	99.33%
44,441	4.6478	22	99.33%
22,371	4.3497	23	99.33%
11,261	4.0516	24	99.33%
5,668	3.7535	25	99.33%
2,853	3.4554	26	99.33%
1,436	3.1573	27	99.33%
723	2.8591	28	99.33%
364	2.5610	29	99.33%
183	2.2629	30	99.33%
92	1.9648	31	99.33%
46	1.6667	32	99.33%
23	1.3686	33	99.33%
12	1.0705	34	99.33%
6	0.7724	35	N/A

** cells highlighted in light blue are below detection threshold limit*

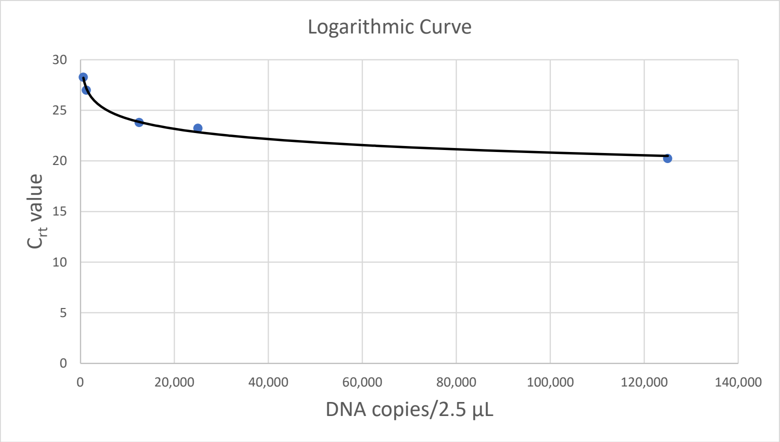

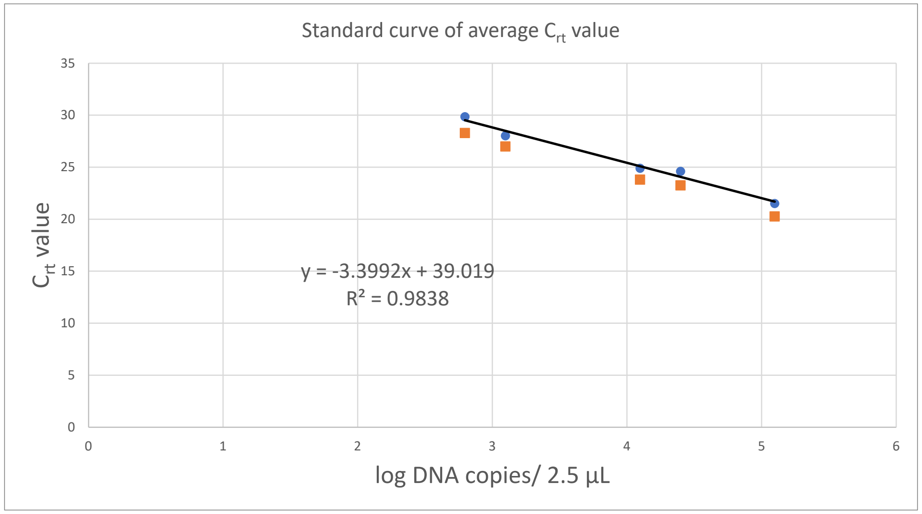

C. parapsilosis	1st C_{rt} Value	2nd C_{rt} Value	3rd C_{rt} Value	Average C_{rt} Value
125,000 copies/2.5 µL (1:1000)	20.741	20.862	20.952	20.852
25,000 copies/2.5 µL (1:5000)	24.790	23.947	23.695	24.144
12,500 copies/2.5 µL (1:10,000)	24.128	24.228	24.136	24.164
1,250 copies/2.5 µL (1:100,000)	28.433	28.259	27.585	28.092
625 copies/2.5 µL (1:200,000)	28.107	27.484	28.346	27.979

Logarithmic curve	
DNA copies/2.5 µL	C_{rt} value
125,000	20.852
25,000	24.144
12,500	24.164
1,250	28.092
625	27.979

Standard curve data of average C_{rt} value		
DNA copies/2.5 µL	log DNA copies/ 2.5 µL	C_{rt} value
125,000	5.096910013	20.852
25,000	4.397940009	24.144
12,500	4.096910013	24.164
1,250	3.096910013	28.092
625	2.795880017	27.979

* Using the average Ct-value mean resulted in the same equation.

y=-3.1613x + 37.365			
DNA copies/2.5 µL	log DNA copies/2.5µL (x)	C_{rt} value (y)	PCR Efficiency
11,874,660	7.0746	15	100.00%
5,731,861	6.7583	16	100.00%
2,766,751	6.4420	17	100.00%
1,335,502	6.1256	18	100.00%
644,643	5.8093	19	100.00%
311,167	5.4930	20	100.00%
150,199	5.1767	21	100.00%
72,501	4.8603	22	100.00%
34,996	4.5440	23	100.00%
16,892	4.2277	24	100.00%
8,154	3.9114	25	100.00%
3,936	3.5950	26	100.00%
1,900	3.2787	27	100.00%
917	2.9624	28	100.00%
443	2.6461	29	100.00%
214	2.3297	30	100.00%
103	2.0134	31	100.00%
50	1.6971	32	100.00%
24	1.3808	33	100.00%
12	1.0644	34	100.00%
6	0.7481	35	N/A

** cells highlighted in light blue are below detection threshold limit*
*** PCR efficiency is a calculation where the theoretical maximum is 100%. If the equation has calculated a PCR efficiency over 100%, the maximum value of 100% is represented.*

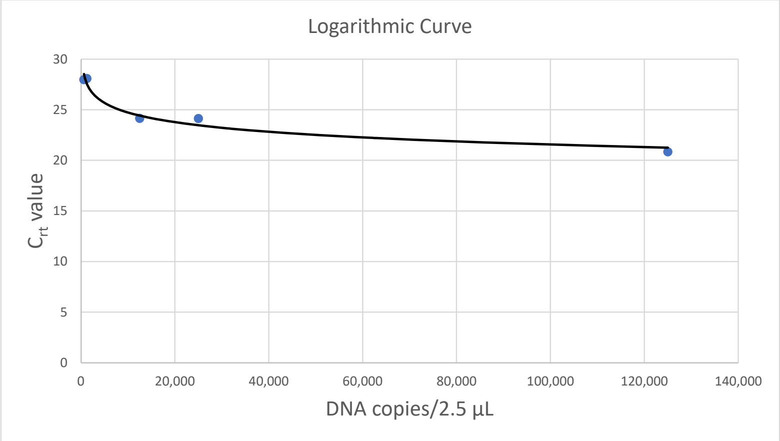

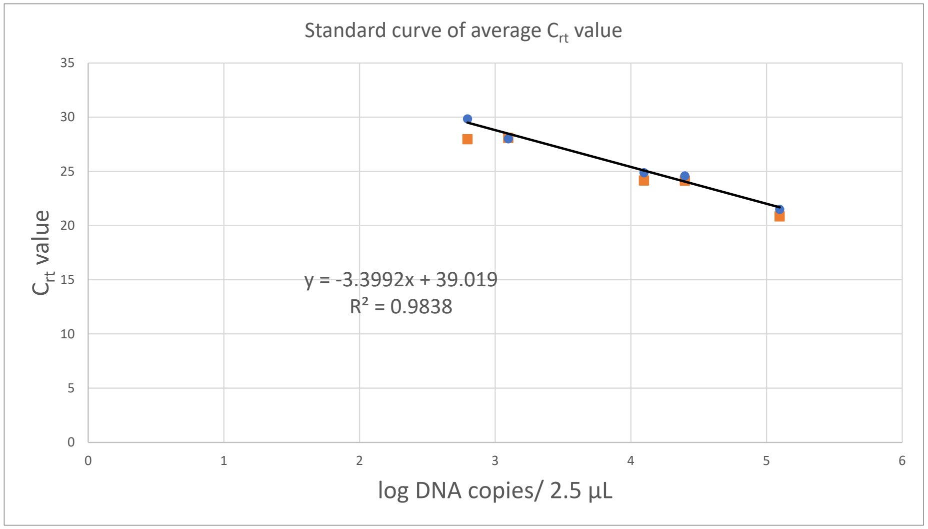

C. riegellii	1st C_{rt} Value	2nd C_{rt} Value	3rd C_{rt} Value	Average C_{rt} Value
125,000 copies/2.5 µL (1:1000)	20.240	20.315	20.412	20.322
25,000 copies/2.5 µL (1:5000)	24.181	23.828	23.138	23.716
12,500 copies/2.5 µL (1:10,000)	23.628	23.862	23.925	23.805
1,250 copies/2.5 µL (1:100,000)	27.387	27.313	27.846	27.515
625 copies/2.5 µL (1:200,000)	28.693	28.928	27.704	28.442

Logarithmic curve	
DNA copies/2.5 µL	C_{rt} value
125,000	20.322
25,000	23.716
12,500	23.805
1,250	27.515
625	28.442

Standard curve data of average C_{rt} value		
DNA copies/2.5 µL	log DNA copies/ 2.5 µL	C_{rt} value
125,000	5.096910013	20.322
25,000	4.397940009	23.716
12,500	4.096910013	23.805
1,250	3.096910013	27.515
625	2.795880017	28.442

* Using the average Ct-value mean resulted in the same equation.

y=-3.4318x + 38.134			
DNA copies/2.5 µL	log DNA copies/2.5µL (x)	C_{rt} value (y)	PCR Efficiency
5,508,950	6.7411	15	97.81%
2,816,285	6.4497	16	97.81%
1,439,741	6.1583	17	97.81%
736,024	5.8669	18	97.81%
376,270	5.5755	19	97.81%
192,357	5.2841	20	97.81%
98,337	4.9927	21	97.81%
50,272	4.7013	22	97.81%
25,700	4.4099	23	97.81%
13,138	4.1185	24	97.81%
6,717	3.8271	25	97.81%
3,434	3.5358	26	97.81%
1,755	3.2444	27	97.81%
897	2.9530	28	97.81%
459	2.6616	29	97.81%
235	2.3702	30	97.81%
120	2.0788	31	97.81%
61	1.7874	32	97.81%
31	1.4960	33	97.81%
16	1.2046	34	97.81%
8	0.9132	35	N/A

* *cells highlighted in light blue are below detection threshold limit*

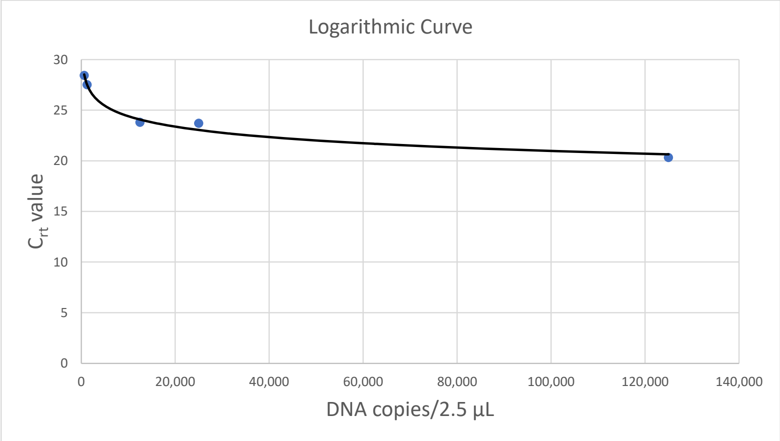

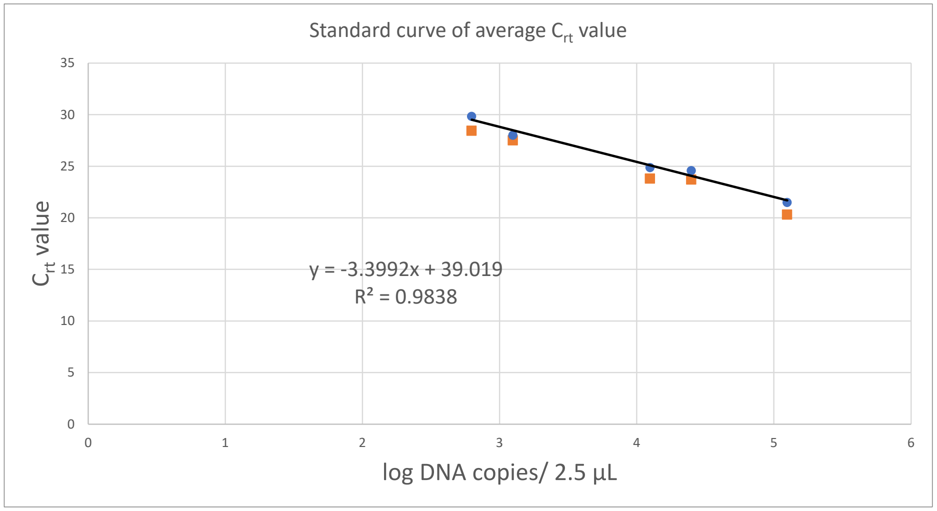

C. urealyticum	1st C_{rt} Value	2nd C_{rt} Value	3rd C_{rt} Value	Average C_{rt} Value
125,000 copies/2.5 µL (1:1000)	20.777	20.835	20.825	20.812
25,000 copies/2.5 µL (1:5000)	24.250	23.997	24.233	24.160
12,500 copies/2.5 µL (1:10,000)	24.605	24.486	24.486	24.526
1,250 copies/2.5 µL (1:100,000)	27.801	27.358	29.537	28.232
625 copies/2.5 µL (1:200,000)	28.525	29.050	29.107	28.894

Logarithmic curve	
DNA copies/2.5 µL	C_{rt} value
125,000	20.812
25,000	24.160
12,500	24.526
1,250	28.232
625	28.894

Standard curve data of average C_{rt} value		
DNA copies/2.5 µL	log DNA copies/ 2.5 µL	C_{rt} value
125,000	5.096910013	20.812
25,000	4.397940009	24.160
12,500	4.096910013	24.526
1,250	3.096910013	28.232
625	2.795880017	28.894

* Using the average Ct-value mean resulted in the same equation.

y=-3.4644x + 38.825			
DNA copies/2.5 µL	log DNA copies/2.5µL (x)	C_{rt} value (y)	PCR Efficiency
7,535,164	6.8771	15	97.19%
3,876,524	6.5884	16	97.19%
1,994,308	6.2998	17	97.19%
1,025,987	6.0111	18	97.19%
527,827	5.7225	19	97.19%
271,545	5.4338	20	97.19%
139,698	5.1452	21	97.19%
71,869	4.8565	22	97.19%
36,973	4.5679	23	97.19%
19,021	4.2792	24	97.19%
9,786	3.9906	25	97.19%
5,034	3.7019	26	97.19%
2,590	3.4133	27	97.19%
1,332	3.1246	28	97.19%
685	2.8360	29	97.19%
353	2.5473	30	97.19%
181	2.2587	31	97.19%
93	1.9700	32	97.19%
48	1.6814	33	97.19%
25	1.3927	34	97.19%
13	1.1041	35	N/A

** cells highlighted in light blue are below detection threshold limit*

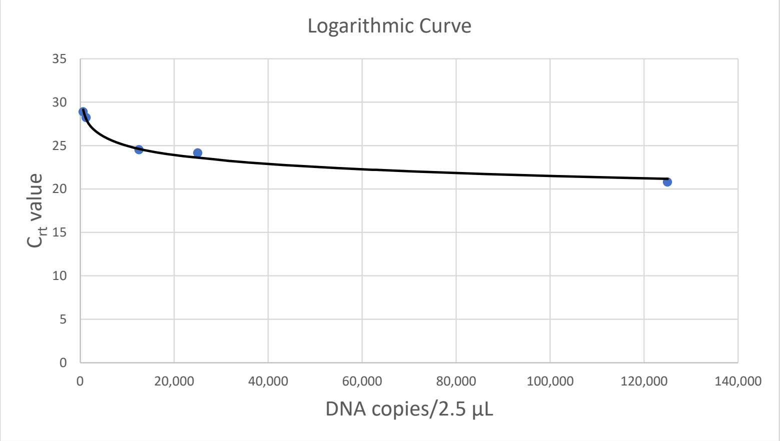

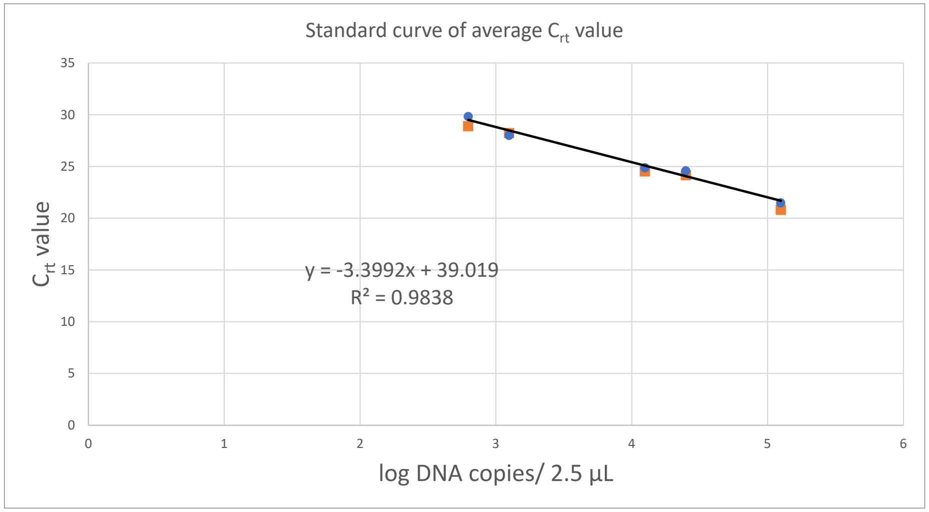

C. trachomatis	1st C_{rt} Value	2nd C_{rt} Value	3rd C_{rt} Value	Average C_{rt} Value
125,000 copies/2.5 µL (1:1000)	20.237	20.302	20.441	20.327
25,000 copies/2.5 µL (1:5000)	23.581	23.566	23.563	23.570
12,500 copies/2.5 µL (1:10,000)	23.869	23.831	23.665	23.788
1,250 copies/2.5 µL (1:100,000)	27.221	26.952	27.530	27.234
625 copies/2.5 µL (1:200,000)	28.586	27.861	28.193	28.213

Logarithmic curve	
DNA copies/2.5 µL	C_{rt} value
125,000	20.327
25,000	23.570
12,500	23.788
1,250	27.234
625	28.213

Standard curve data of average C_{rt} value		
DNA copies/2.5 µL	log DNA copies/ 2.5 µL	C_{rt} value
125,000	5.096910013	20.327
25,000	4.397940009	23.570
12,500	4.096910013	23.788
1,250	3.096910013	27.234
625	2.795880017	28.213

* Using the average Ct-value mean resulted in the same equation.

y=-3.3189x + 37.56			
DNA copies/2.5 µL	log DNA copies/2.5µL (x)	C_{rt} value (y)	PCR Efficiency
6,272,388	6.7974	15	100.00%
3,134,211	6.4961	16	100.00%
1,566,115	6.1948	17	100.00%
782,562	5.8935	18	100.00%
391,034	5.5922	19	100.00%
195,393	5.2909	20	100.00%
97,635	4.9896	21	100.00%
48,787	4.6883	22	100.00%
24,378	4.3870	23	100.00%
12,181	4.0857	24	100.00%
6,087	3.7844	25	100.00%
3,041	3.4831	26	100.00%
1,520	3.1818	27	100.00%
759	2.8805	28	100.00%
379	2.5792	29	100.00%
190	2.2779	30	100.00%
95	1.9766	31	100.00%
47	1.6753	32	100.00%
24	1.3739	33	100.00%
12	1.0726	34	100.00%
6	0.7713	35	N/A

** cells highlighted in light blue are below detection threshold limit*

*** PCR efficiency is a calculation where the theoretical maximum is 100%. If the equation has calculated a PCR efficiency over 100%, the maximum value of 100% is represented.*

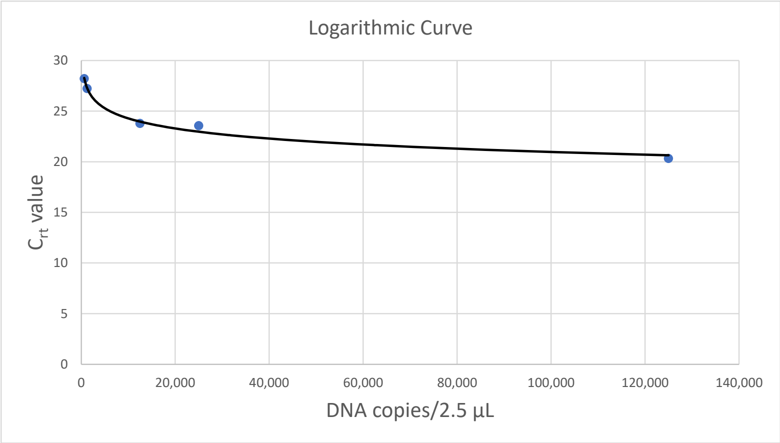

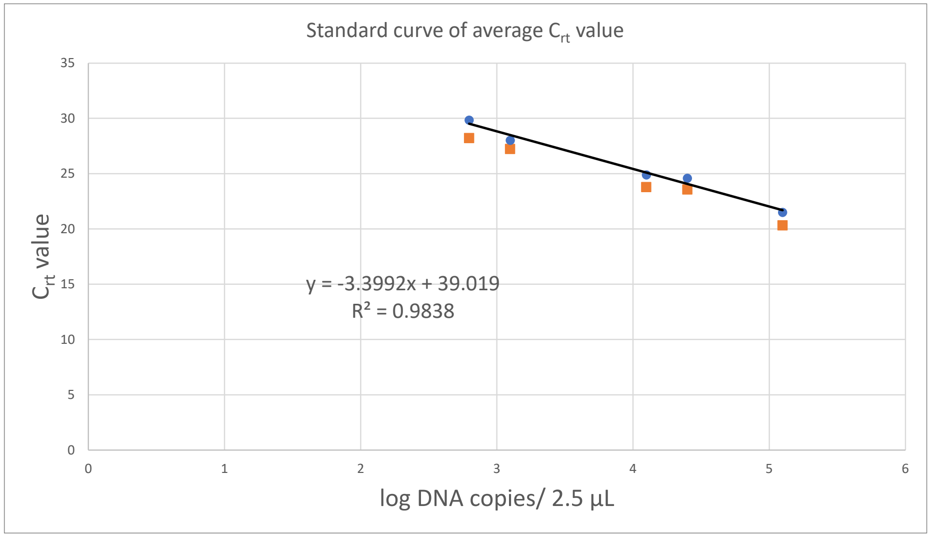

N. gonorrhoeae	1st C_{rt} Value	2nd C_{rt} Value	3rd C_{rt} Value	Average C_{rt} Value
125,000 copies/2.5 µL (1:1000)	20.650	20.611	20.680	20.647
25,000 copies/2.5 µL (1:5000)	24.056	23.863	23.893	23.937
12,500 copies/2.5 µL (1:10,000)	23.825	23.893	24.234	23.984
1,250 copies/2.5 µL (1:100,000)	28.140	28.489	28.280	28.303
625 copies/2.5 µL (1:200,000)	28.899	28.980	29.044	28.974

Logarithmic curve	
DNA copies/2.5 µL	C_{rt} value
125,000	20.647
25,000	23.937
12,500	23.984
1,250	28.303
625	28.974

Standard curve data of average C_{rt} value		
DNA copies/2.5 µL	log DNA copies/ 2.5 µL	C_{rt} value
125,000	5.096910013	20.647
25,000	4.397940009	23.937
12,500	4.096910013	23.984
1,250	3.096910013	28.303
625	2.795880017	28.974

* Using the average Ct-value mean resulted in the same equation.

y=-3.6217x + 39.282

DNA copies/2.5 µL	log DNA copies/2.5µL (x)	C_{rt} value (y)	PCR Efficiency
5,065,079	6.7046	15	94.42%
2,682,087	6.4285	16	94.42%
1,420,232	6.1524	17	94.42%
752,049	5.8762	18	94.42%
398,229	5.6001	19	94.42%
210,872	5.3240	20	94.42%
111,662	5.0479	21	94.42%
59,128	4.7718	22	94.42%
31,310	4.4957	23	94.42%
16,579	4.2196	24	94.42%
8,779	3.9435	25	94.42%
4,649	3.6673	26	94.42%
2,462	3.3912	27	94.42%
1,304	3.1151	28	94.42%
690	2.8390	29	94.42%
365	2.5629	30	94.42%
194	2.2868	31	94.42%
102	2.0107	32	94.42%
54	1.7345	33	94.42%
29	1.4584	34	94.42%
15	1.1823	35	N/A

** cells highlighted in light blue are below detection threshold limit*

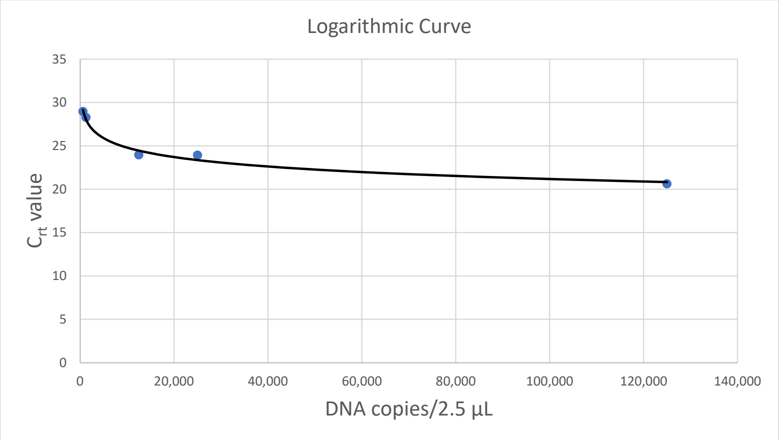

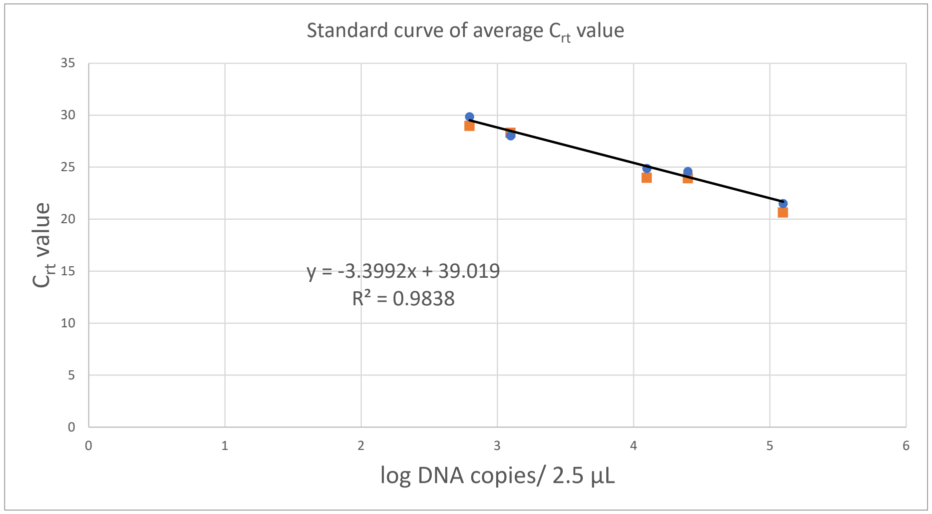

T. vaginalis	1st C_{rt} Value	2nd C_{rt} Value	3rd C_{rt} Value	Average C_{rt} Value
125,000 copies/2.5 µL (1:1000)	21.050	21.274	21.129	21.151
25,000 copies/2.5 µL (1:5000)	24.688	24.574	23.899	24.387
12,500 copies/2.5 µL (1:10,000)	24.606	24.769	24.792	24.722
1,250 copies/2.5 µL (1:100,000)	28.272	28.449	28.104	28.275
625 copies/2.5 µL (1:200,000)	29.028	28.795	29.592	29.138

Logarithmic curve	
DNA copies/2.5 µL	C_{rt} value
125,000	21.151
25,000	24.387
12,500	24.722
1,250	28.275
625	29.138

Standard curve data of average C_{rt} value		
DNA copies/2.5 µL	log DNA copies/ 2.5 µL	C_{rt} value
125,000	5.096910013	21.151
25,000	4.397940009	24.387
12,500	4.096910013	24.722
1,250	3.096910013	28.275
625	2.795880017	29.138

* Using the average Ct-value mean resulted in the same equation.

y=-3.3929x + 38.757			
DNA copies/2.5 µL	log DNA copies/2.5µL (x)	C_{rt} value (y)	PCR Efficiency
10,045,573	7.0020	15	98.56%
5,096,143	6.7072	16	98.56%
2,585,285	6.4125	17	98.56%
1,311,521	6.1178	18	98.56%
665,338	5.8230	19	98.56%
337,527	5.5283	20	98.56%
171,228	5.2336	21	98.56%
86,865	4.9388	22	98.56%
44,067	4.6441	23	98.56%
22,355	4.3494	24	98.56%
11,341	4.0546	25	98.56%
5,753	3.7599	26	98.56%
2,919	3.4652	27	98.56%
1,481	3.1704	28	98.56%
751	2.8757	29	98.56%
381	2.5810	30	98.56%
193	2.2862	31	98.56%
98	1.9915	32	98.56%
50	1.6968	33	98.56%
25	1.4020	34	98.56%
13	1.1073	35	N/A

** cells highlighted in light blue are below detection threshold limit*

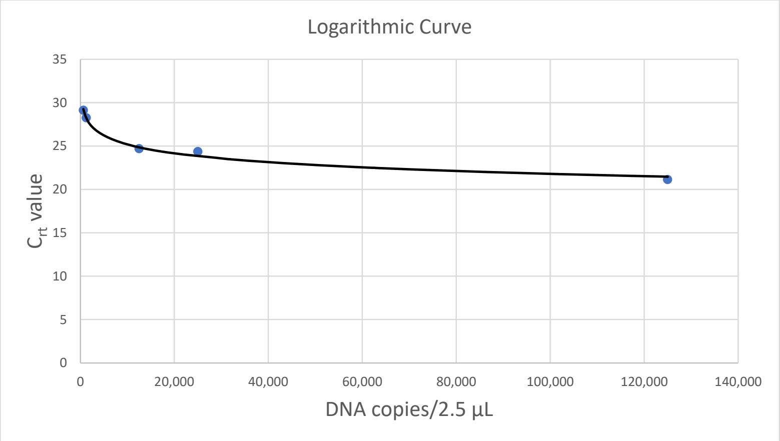

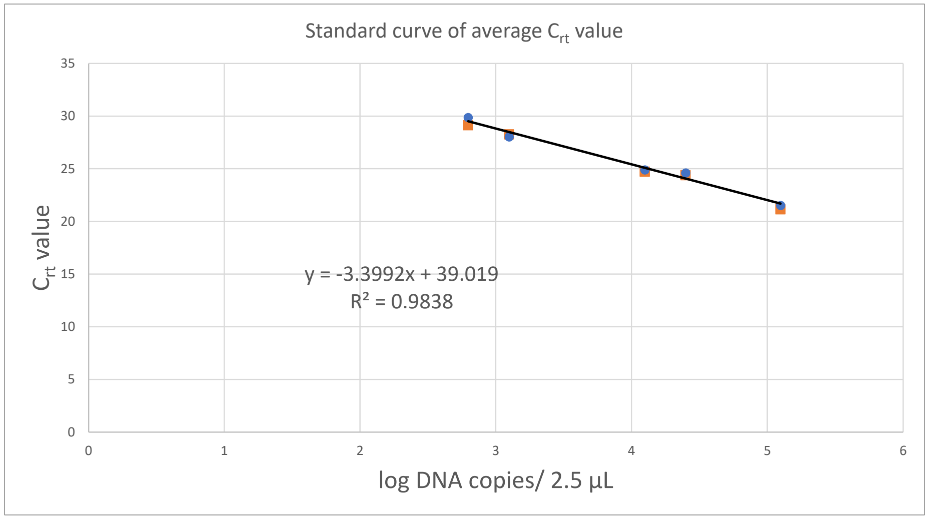

S. pasteuranus	1st C_{rt} Value	2nd C_{rt} Value	3rd C_{rt} Value	Average C_{rt} Value
125,000 copies/2.5 µL (1:1000)	21.379	21.391	21.336	21.369
25,000 copies/2.5 µL (1:5000)	24.703	24.814	24.695	24.737
12,500 copies/2.5 µL (1:10,000)	25.014	25.147	24.842	25.001
1,250 copies/2.5 µL (1:100,000)	28.720	28.410	28.507	28.546
625 copies/2.5 µL (1:200,000)	29.710	29.238	30.448	29.799

Logarithmic curve	
DNA copies/2.5 µL	C_{rt} value
125,000	21.369
25,000	24.737
12,500	25.001
1,250	28.546
625	29.799

Standard curve data of average C_{rt} value		
DNA copies/2.5 µL	log DNA copies/ 2.5 µL	C_{rt} value
125,000	5.096910013	21.369
25,000	4.397940009	24.737
12,500	4.096910013	25.001
1,250	3.096910013	28.546
625	2.795880017	29.799

* Using the average Ct-value mean resulted in the same equation.

y=-3.5188x + 39.603			
DNA copies/2.5 µL	log DNA copies/2.5µL (x)	C_{rt} value (y)	PCR Efficiency
9,814,592	6.9919	15	96.20%
5,101,342	6.7077	16	96.20%
2,651,531	6.4235	17	96.20%
1,378,189	6.1393	18	96.20%
716,343	5.8551	19	96.20%
372,334	5.5709	20	96.20%
193,529	5.2867	21	96.20%
100,591	5.0026	22	96.20%
52,284	4.7184	23	96.20%
27,176	4.4342	24	96.20%
14,125	4.1500	25	96.20%
7,342	3.8658	26	96.20%
3,816	3.5816	27	96.20%
1,983	3.2974	28	96.20%
1,031	3.0132	29	96.20%
536	2.7291	30	96.20%
279	2.4449	31	96.20%
145	2.1607	32	96.20%
75	1.8765	33	96.20%
39	1.5923	34	96.20%
20	1.3081	35	N/A

** cells highlighted in light blue are below detection threshold limit*

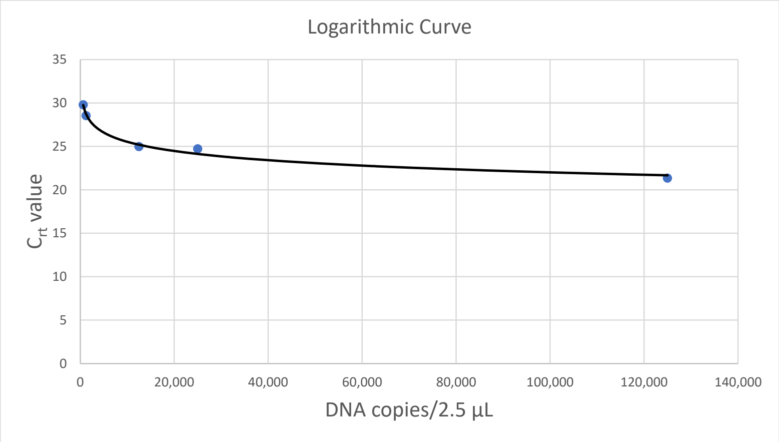

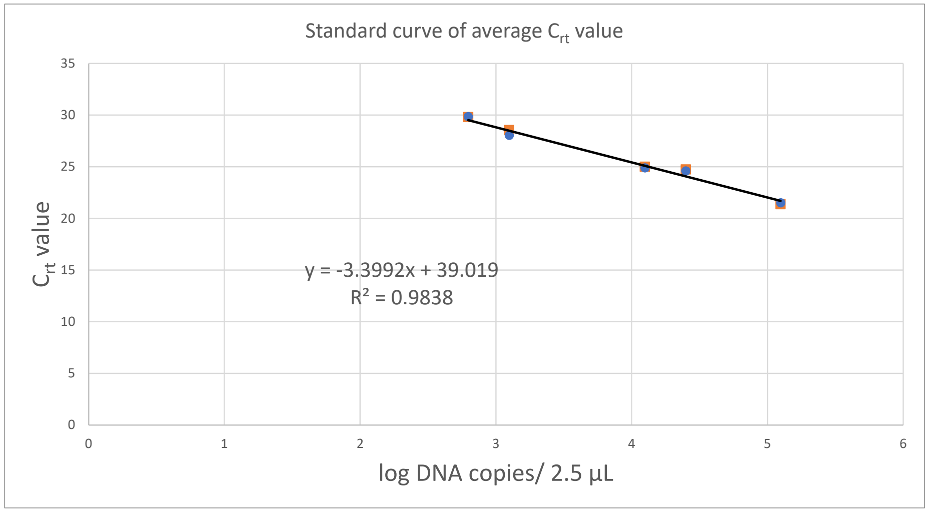

S. pyogenes	1st C_{rt} Value	2nd C_{rt} Value	3rd C_{rt} Value	Average C_{rt} Value
125,000 copies/2.5 µL (1:1000)	20.684	20.856	20.771	20.770
25,000 copies/2.5 µL (1:5000)	23.993	23.856	24.072	23.974
12,500 copies/2.5 µL (1:10,000)	24.218	24.379	24.374	24.324
1,250 copies/2.5 µL (1:100,000)	28.153	27.409	28.650	28.071
625 copies/2.5 µL (1:200,000)	29.073	28.631		28.852

Logarithmic curve	
DNA copies/2.5 µL	C_{rt} value
125,000	20.770
25,000	23.974
12,500	24.324
1,250	28.071
625	28.852

Standard curve data of average C_{rt} value		
DNA copies/2.5 µL	log DNA copies/ 2.5 µL	C_{rt} value
125,000	5.096910013	20.770
25,000	4.397940009	23.974
12,500	4.096910013	24.324
1,250	3.096910013	28.071
625	2.795880017	28.852

* Using the average Ct-value mean resulted in the same equation.

y=-3.4669x + 38.708

DNA copies/2.5 µL	log DNA copies/2.5µL (x)	C_{rt} value (y)	PCR Efficiency
6,892,644	6.8384	15	97.14%
3,547,674	6.5499	16	97.14%
1,826,004	6.2615	17	97.14%
939,852	5.9731	18	97.14%
483,746	5.6846	19	97.14%
248,986	5.3962	20	97.14%
128,154	5.1077	21	97.14%
65,962	4.8193	22	97.14%
33,951	4.5308	23	97.14%
17,475	4.2424	24	97.14%
8,994	3.9540	25	97.14%
4,629	3.6655	26	97.14%
2,383	3.3771	27	97.14%
1,226	3.0886	28	97.14%
631	2.8002	29	97.14%
325	2.5118	30	97.14%
167	2.2233	31	97.14%
86	1.9349	32	97.14%
44	1.6464	33	97.14%
23	1.3580	34	97.14%
12	1.0695	35	N/A

** cells highlighted in light blue are below detection threshold limit*

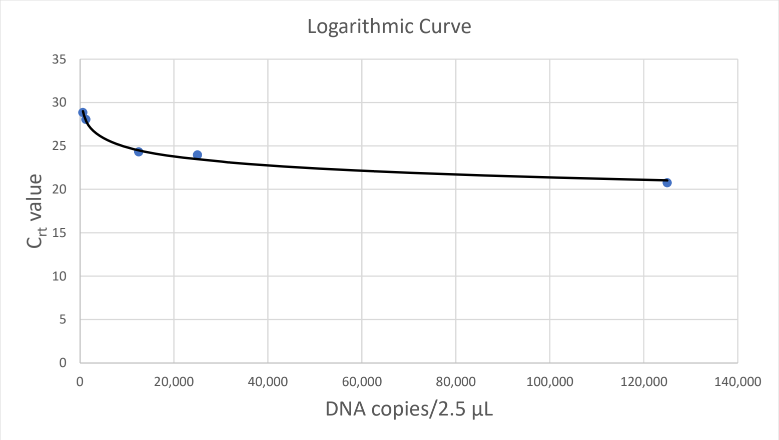

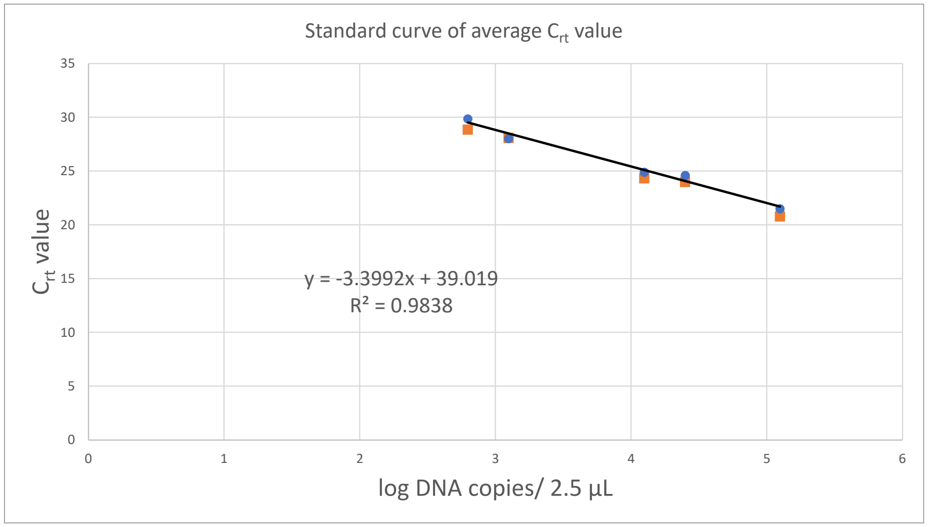

HHV6	1st C_{rt} Value	2nd C_{rt} Value	3rd C_{rt} Value	Average C_{rt} Value
125,000 copies/2.5 µL (1:1000)	20.828	20.971	20.887	20.895
25,000 copies/2.5 µL (1:5000)	24.596	24.463	23.811	24.290
12,500 copies/2.5 µL (1:10,000)	24.408	24.197	24.528	24.378
1,250 copies/2.5 µL (1:100,000)	28.546	28.628	27.777	28.317
625 copies/2.5 µL (1:200,000)	29.019	28.491	28.902	28.804

Logarithmic curve	
DNA copies/2.5 µL	C_{rt} value
125,000	20.895
25,000	24.290
12,500	24.378
1,250	28.317
625	28.804

Standard curve data of average C_{rt} value		
DNA copies/2.5 µL	log DNA copies/ 2.5 µL	C_{rt} value
125,000	5.096910013	20.895
25,000	4.397940009	24.290
12,500	4.096910013	24.378
1,250	3.096910013	28.317
625	2.795880017	28.804

* Using the average Ct-value mean resulted in the same equation.

y=-3.418x + 38.657			
DNA copies/2.5 µL	log DNA copies/2.5µL (x)	C_{rt} value (y)	PCR Efficiency
8,342,554	6.9213	15	98.07%
4,253,341	6.6287	16	98.07%
2,168,510	6.3362	17	98.07%
1,105,587	6.0436	18	98.07%
563,669	5.7510	19	98.07%
287,379	5.4585	20	98.07%
146,516	5.1659	21	98.07%
74,700	4.8733	22	98.07%
38,085	4.5807	23	98.07%
19,417	4.2882	24	98.07%
9,899	3.9956	25	98.07%
5,047	3.7030	26	98.07%
2,573	3.4105	27	98.07%
1,312	3.1179	28	98.07%
669	2.8253	29	98.07%
341	2.5328	30	98.07%
174	2.2402	31	98.07%
89	1.9476	32	98.07%
45	1.6551	33	98.07%
23	1.3625	34	98.07%
12	1.0699	35	N/A

** cells highlighted in light blue are below detection threshold limit*

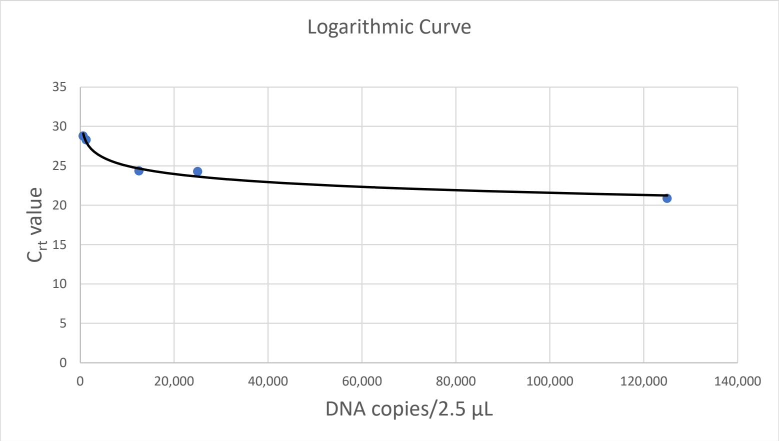

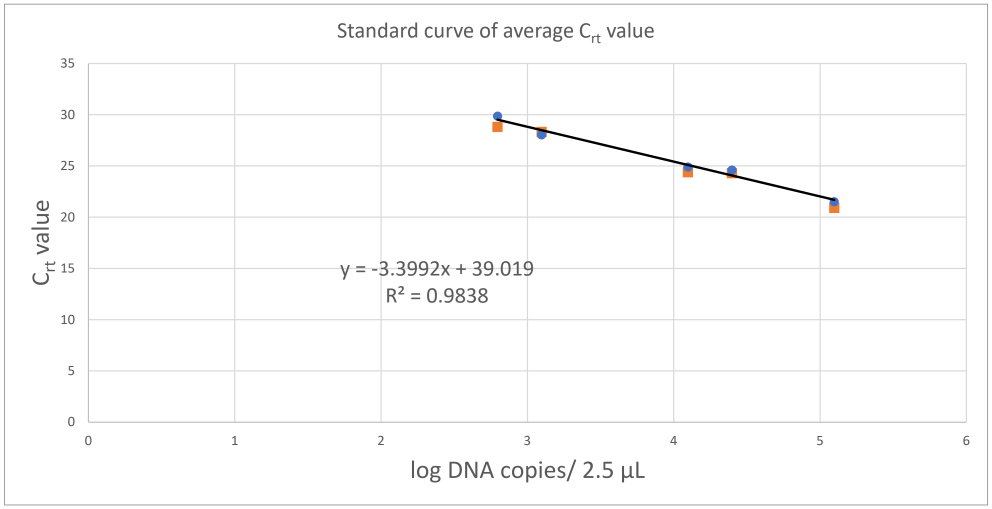

HHV7	1st C_{rt} Value	2nd C_{rt} Value	3rd C_{rt} Value	Average C_{rt} Value
125,000 copies/2.5 µL (1:1000)	20.401	20.458	20.213	20.357
25,000 copies/2.5 µL (1:5000)	23.877	23.545	23.285	23.569
12,500 copies/2.5 µL (1:10,000)	23.790	23.977	23.923	23.897
1,250 copies/2.5 µL (1:100,000)	28.029	27.451	26.946	27.475
625 copies/2.5 µL (1:200,000)	27.136	29.793	28.565	28.498

Logarithmic curve	
DNA copies/2.5 µL	C_{rt} value
125,000	20.357
25,000	23.569
12,500	23.897
1,250	27.475
625	28.498

Standard curve data of average C_{rt} value		
DNA copies/2.5 µL	log DNA copies/ 2.5 µL	C_{rt} value
125,000	5.096910013	20.357
25,000	4.397940009	23.569
12,500	4.096910013	23.897
1,250	3.096910013	27.475
625	2.795880017	28.498

* Using the average Ct-value mean resulted in the same equation.

y=-3.4439x + 38.18			
DNA copies/2.5 µL	log DNA copies/2.5µL (x)	C_{rt} value (y)	PCR Efficiency
5,379,493	6.7307	15	97.57%
2,756,594	6.4404	16	97.57%
1,412,552	6.1500	17	97.57%
723,829	5.8596	18	97.57%
370,909	5.5693	19	97.57%
190,064	5.2789	20	97.57%
97,394	4.9885	21	97.57%
49,907	4.6982	22	97.57%
25,574	4.4078	23	97.57%
13,105	4.1174	24	97.57%
6,715	3.8271	25	97.57%
3,441	3.5367	26	97.57%
1,763	3.2463	27	97.57%
904	2.9560	28	97.57%
463	2.6656	29	97.57%
237	2.3752	30	97.57%
122	2.0848	31	97.57%
62	1.7945	32	97.57%
32	1.5041	33	97.57%
16	1.2137	34	97.57%
8	0.9234	35	N/A

** cells highlighted in light blue are below detection threshold limit*

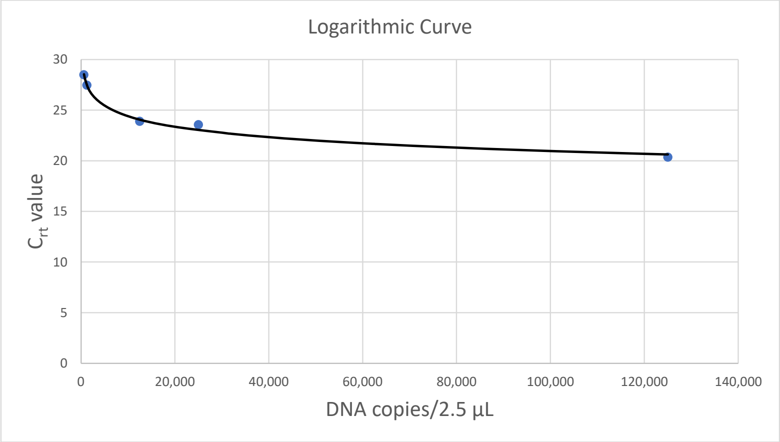

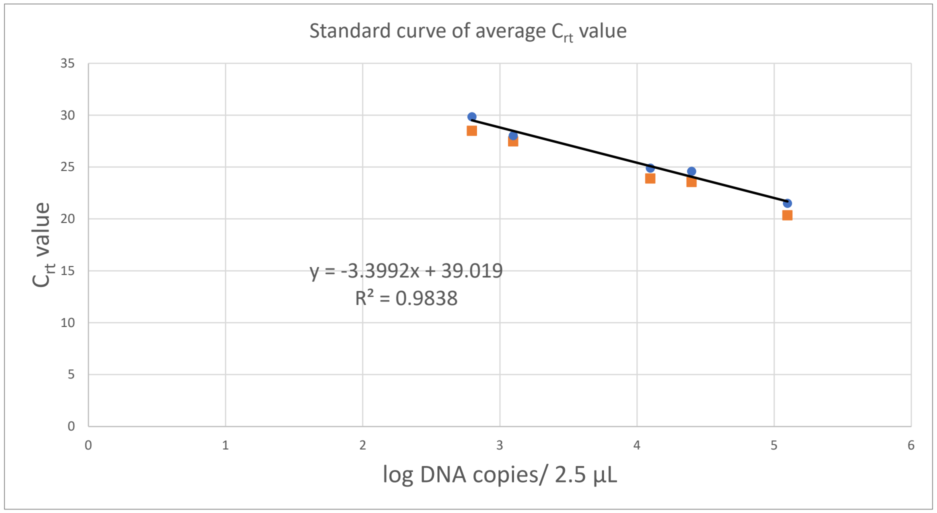

The Impact of Polymerase Chain Reaction Urine Testing on Clinical Decision-Making in the Management of Complex Uri-nary Tract Infections

Supplement C – UTIdv2 CARD Antibiotic Resistance

Julia Elia, Jason Hafron, Mara Holton, Connor Ervin, Mitchell B. Hollander, and Deepak A. Kapoor

Gene symbol	ThermoFisher (TF) Assay ID	Gene Name	CARD Link	Drug Class(es)	Resistance Mechanism
VanA	Ba04646147_s1	D-alanine--(R)-lactate ligase VanA	https://card.mcmaster.ca/ontology/36019	glycopeptide antibiotic	antibiotic target alteration
blaOXA	Ba04646133_s1	OXA-1 family class D beta-lactamase, blaOXA-1, blaOXA-1042, blaOXA-224, blaOXA-31, blaOXA-320, blaOXA-392, blaOXA-4, blaOXA-47, blaOXA-534, blaOXA-675, blaOXA-796	https://card.mcmaster.ca/ontology/37796	penam, cephalosporin	antibiotic inactivation
blaFOX	Ba04646126_s1	cephalosporin-hydrolyzing class C beta-lactamase FOX, blaFOX-1, blaFOX-10, blaFOX-12, blaFOX-13, blaFOX-14, blaFOX-15, blaFOX-16, blaFOX-17, blaFOX-18, blaFOX-19, blaFOX-3, blaFOX-4, blaFOX-5, blaFOX-7, blaFOX-8	https://card.mcmaster.ca/ontology/38555	cephalosporin, cephamycin	antibiotic inactivation
blaGES	Ba04646151_s1	class A beta-lactamase GES, blaGES-1, blaGES-10, blaGES-11, blaGES-12, blaGES-13, blaGES-14, blaGES-15, blaGES-16, blaGES-17, blaGES-18, blaGES-19, blaGES-2, blaGES-20, blaGES-21, blaGES-22, blaGES-24, blaGES-25, blaGES-26, blaGES-27, blaGES-28, blaGES-29, blaGES-3, blaGES-30, blaGES-31, blaGES-33, blaGES-34, blaGES-35, blaGES-36, blaGES-37, blaGES-38, blaGES-39, blaGES-4, blaGES-40, blaGES-42, blaGES-43, blaGES-45, blaGES-46, blaGES-47, blaGES-48, blaGES-49, blaGES-5, blaGES-50, blaGES-51, blaGES-6, blaGES-7, blaGES-8, blaGES-9	https://card.mcmaster.ca/ontology/38730	cephalosporin, penam, carbapenem	antibiotic inactivation
blaKPC	Ba04646152_s1	carbapenem-hydrolyzing class A beta-lactamase KPC, blaKPC-10, blaKPC-102, blaKPC-103, blaKPC-104, blaKPC-105, blaKPC-106, blaKPC-107, blaKPC-108, blaKPC-11, blaKPC-112, blaKPC-113, blaKPC-114, blaKPC-115, blaKPC-116, blaKPC-117, blaKPC-118, blaKPC-12, blaKPC-120, blaKPC-121, blaKPC-122, blaKPC-123, blaKPC-13, blaKPC-14, blaKPC-15, blaKPC-16, blaKPC-26, blaKPC-27, blaKPC-28, blaKPC-29, blaKPC-3, blaKPC-30, blaKPC-33, blaKPC-34, blaKPC-35, blaKPC-36, blaKPC-37, blaKPC-38, blaKPC-39, blaKPC-4, blaKPC-41, blaKPC-42, blaKPC-43, blaKPC-44, blaKPC-45, blaKPC-46, blaKPC-47, blaKPC-48, blaKPC-49, blaKPC-5, blaKPC-50, blaKPC-51, blaKPC-52, blaKPC-53, blaKPC-54, blaKPC-55, blaKPC-56, blaKPC-57, blaKPC-58, blaKPC-59, blaKPC-6, blaKPC-60, blaKPC-61, blaKPC-62, blaKPC-63, blaKPC-64, blaKPC-65, blaKPC-66, blaKPC-67, blaKPC-68, blaKPC-69, blaKPC-7, blaKPC-70, blaKPC-71, blaKPC-72, blaKPC-73, blaKPC-74, blaKPC-75, blaKPC-76, blaKPC-77, blaKPC-78, blaKPC-79, blaKPC-8, blaKPC-80, blaKPC-81, blaKPC-82, blaKPC-83, blaKPC-84, blaKPC-85, blaKPC-86, blaKPC-87, blaKPC-88, blaKPC-89, blaKPC-90, blaKPC-91, blaKPC-92, blaKPC-94, blaKPC-95, blaKPC-96, blaKPC-97, blaKPC-98	https://card.mcmaster.ca/ontology/38720	penam, cephalosporin, monobactam, carbapenem	antibiotic inactivation
blaIMP	Ba04646131_s1	subclass B1 metallo-beta-lactamase IMP, blaIMP-1, blaIMP-10, blaIMP-25, blaIMP-26, blaIMP-28, blaIMP-29, blaIMP-3, blaIMP-30, blaIMP-34, blaIMP-38, blaIMP-4, blaIMP-40, blaIMP-42, blaIMP-5, blaIMP-59, blaIMP-6, blaIMP-60, blaIMP-61, blaIMP-66, blaIMP-70, blaIMP-76, blaIMP-77, blaIMP-78, blaIMP-79, blaIMP-80, blaIMP-85, blaIMP-88, blaIMP-89, blaIMP-94	https://card.mcmaster.ca/ontology/38592	cephamycin, cephalosporin, carbapenem, penem, penam	antibiotic inactivation

Gene symbol	ThermoFisher (TF) Assay ID	Gene Name	CARD Link	Drug Class(es)	Resistance Mechanism
blaNDM	Ba04931076_s1	subclass B1 metallo-beta-lactamase, blaNDM-1, blaNDM-10, blaNDM-11, blaNDM-12, blaNDM-13, blaNDM-14, blaNDM-15, blaNDM-16a, blaNDM-16b, blaNDM-17, blaNDM-18, blaNDM-19, blaNDM-2, blaNDM-20, blaNDM-21, blaNDM-22, blaNDM-23, blaNDM-24, blaNDM-25, blaNDM-26, blaNDM-27, blaNDM-28, blaNDM-29, blaNDM-3, blaNDM-30, blaNDM-31, blaNDM-34, blaNDM-35, blaNDM-37, blaNDM-38, blaNDM-39, blaNDM-4, blaNDM-40, blaNDM-41, blaNDM-5, blaNDM-6, blaNDM-7, blaNDM-8, blaNDM-9	https://card.mcmaster.ca/ontology/36728	carbapenem, penam, cephamycin, cephalosporin	antibiotic inactivation
blaOXA	Ba04930816_s1	OXA-48 family class D beta-lactamase OXA, blaOXA-162, blaOXA-199, blaOXA-244, blaOXA-245, blaOXA-252, blaOXA-370, blaOXA-48, blaOXA-505, blaOXA-514, blaOXA-519, blaOXA-566, blaOXA-918, blaOXA-933, blaOXA-934	https://card.mcmaster.ca/ontology/38176	penam, carbapenem	antibiotic inactivation
blaPER	Ba04646140_s1	class A extended-spectrum beta-lactamase PER, blaPER-1, blaPER-11, blaPER-13, blaPER-15, blaPER-16, blaPER-3, blaPER-4, blaPER-5, blaPER-7, blaPER-8, blaPER-9	https://card.mcmaster.ca/ontology/38763	penem, penam, cephalosporin, carbapenem, monobactam	antibiotic inactivation
blaVEB	Ba04646153_s1	class A extended-spectrum beta-lactamase, blaVEB-1, blaVEB-10, blaVEB-11, blaVEB-12, blaVEB-15, blaVEB-16, blaVEB-17, blaVEB-18, blaVEB-19, blaVEB-2, blaVEB-20, blaVEB-21, blaVEB-22, blaVEB-23, blaVEB-24, blaVEB-26, blaVEB-27, blaVEB-28, blaVEB-29, blaVEB-3, blaVEB-30, blaVEB-4, blaVEB-5, blaVEB-6, blaVEB-7, blaVEB-9	https://card.mcmaster.ca/ontology/38770	monobactam, cephalosporin	antibiotic inactivation
blaCTX-M	Ba04646149_s1	class A extended-spectrum beta-lactamase CTX-M , blaCTX-M-101, blaCTX-M-103, blaCTX-M-114, blaCTX-M-116, blaCTX-M-117, blaCTX-M-12, blaCTX-M-123, blaCTX-M-127, blaCTX-M-136, blaCTX-M-139, blaCTX-M-142, blaCTX-M-143, blaCTX-M-144, blaCTX-M-15, blaCTX-M-150, blaCTX-M-153, blaCTX-M-154, blaCTX-M-155, blaCTX-M-156, blaCTX-M-157, blaCTX-M-162, blaCTX-M-163, blaCTX-M-164, blaCTX-M-167, blaCTX-M-169, blaCTX-M-170, blaCTX-M-172, blaCTX-M-173, blaCTX-M-176, blaCTX-M-177, blaCTX-M-178, blaCTX-M-179, blaCTX-M-180, blaCTX-M-181, blaCTX-M-182, blaCTX-M-183, blaCTX-M-184, blaCTX-M-186, blaCTX-M-187, blaCTX-M-188, blaCTX-M-189, blaCTX-M-190, blaCTX-M-193, blaCTX-M-194, blaCTX-M-197, blaCTX-M-202, blaCTX-M-203, blaCTX-M-204, blaCTX-M-206, blaCTX-M-207, blaCTX-M-208, blaCTX-M-209, blaCTX-M-210, blaCTX-M-211, blaCTX-M-212, blaCTX-M-216, blaCTX-M-218, blaCTX-M-22, blaCTX-M-220, blaCTX-M-225, blaCTX-M-226, blaCTX-M-227, blaCTX-M-228, blaCTX-M-23, blaCTX-M-230, blaCTX-M-231, blaCTX-M-232, blaCTX-M-236, blaCTX-M-237, blaCTX-M-238, blaCTX-M-244, blaCTX-M-245, blaCTX-M-251, blaCTX-M-28, blaCTX-M-3, blaCTX-M-33, blaCTX-M-42, blaCTX-M-52, blaCTX-M-54, blaCTX-M-55, blaCTX-M-60, blaCTX-M-62, blaCTX-M-66, blaCTX-M-69, blaCTX-M-71, blaCTX-M-72, blaCTX-M-79, blaCTX-M-80, blaCTX-M-82, blaCTX-M-88, blaCTX-M-96	https://card.mcmaster.ca/ontology/38360	cephalosporin	antibiotic inactivation

Gene symbol	ThermoFisher (TF) Assay ID	Gene Name	CARD Link	Drug Class(es)	Resistance Mechanism
blaCTX-M	Ba04646142_s1	class A extended-spectrum beta-lactamase CTX-M, blaCTX-M-115, blaCTX-M-124, blaCTX-M-131, blaCTX-M-141, blaCTX-M-165, blaCTX-M-171, blaCTX-M-2, blaCTX-M-20, blaCTX-M-200, blaCTX-M-229, blaCTX-M-31, blaCTX-M-35, blaCTX-M-4, blaCTX-M-43, blaCTX-M-44, blaCTX-M-5, blaCTX-M-56, blaCTX-M-59, blaCTX-M-6, blaCTX-M-7, blaCTX-M-76, blaCTX-M-77, blaCTX-M-92, blaCTX-M-95, blaCTX-M-97	https://card.mcmaster.ca/ontology/38375	cephalosporin	antibiotic inactivation
blaCTX-M	Ba04646154_s1	class A extended-spectrum beta-lactamase CTX-M, blaCTX-M-100, blaCTX-M-152, blaCTX-M-160, blaCTX-M-185, blaCTX-M-205, blaCTX-M-217, blaCTX-M-25, blaCTX-M-26, blaCTX-M-39, blaCTX-M-40, blaCTX-M-41, blaCTX-M-63, blaCTX-M-78, blaCTX-M-8, blaCTX-M-89, blaCTX-M-91, blaCTX-M-94	https://card.mcmaster.ca/ontology/38359	cephalosporin	antibiotic inactivation
blaCTX-M	Ba04646127_s1	class A extended-spectrum beta-lactamase CTX-M, blaCTX-M-102, blaCTX-M-104, blaCTX-M-105, blaCTX-M-110, blaCTX-M-111, blaCTX-M-112, blaCTX-M-113, blaCTX-M-121, blaCTX-M-122, blaCTX-M-125, blaCTX-M-126, blaCTX-M-129, blaCTX-M-13, blaCTX-M-130, blaCTX-M-134, blaCTX-M-137, blaCTX-M-14, blaCTX-M-147, blaCTX-M-148, blaCTX-M-159, blaCTX-M-16, blaCTX-M-161, blaCTX-M-168, blaCTX-M-17, blaCTX-M-174, blaCTX-M-19, blaCTX-M-191, blaCTX-M-192, blaCTX-M-195, blaCTX-M-196, blaCTX-M-198, blaCTX-M-199, blaCTX-M-201, blaCTX-M-21, blaCTX-M-213, blaCTX-M-214, blaCTX-M-215, blaCTX-M-219, blaCTX-M-221, blaCTX-M-223, blaCTX-M-233, blaCTX-M-235, blaCTX-M-239, blaCTX-M-24, blaCTX-M-240, blaCTX-M-241, blaCTX-M-242, blaCTX-M-243, blaCTX-M-252, blaCTX-M-27, blaCTX-M-38, blaCTX-M-46, blaCTX-M-47, blaCTX-M-48, blaCTX-M-49, blaCTX-M-50, blaCTX-M-51, blaCTX-M-64, blaCTX-M-65, blaCTX-M-67, blaCTX-M-73, blaCTX-M-83, blaCTX-M-84, blaCTX-M-85, blaCTX-M-86, blaCTX-M-87, blaCTX-M-9, blaCTX-M-90, blaCTX-M-93, blaCTX-M-98, blaCTX-M-99	https://card.mcmaster.ca/ontology/38361	cephalosporin	antibiotic inactivation
erm(B)	Pa04230913_s1	23S rRNA (adenine(2058)-N(6))-methyltransferase Erm(B)	https://card.mcmaster.ca/ontology/36514	streptogramin antibiotic, macrolide antibiotic, lincosamide antibiotic, streptogramin A antibiotic, streptogramin B antibiotic	antibiotic target alteration
qnrA	Ba04646160_s1	quinolone resistance pentapeptide repeat protein QnrA2	https://card.mcmaster.ca/ontology/39142	fluoroquinolone antibiotic	antibiotic target protection
qnrS	Ba04646145_s1	quinolone resistance pentapeptide repeat protein QnrS9 , qnrS1, qnrS10, qnrS11, qnrS15, qnrS4, qnrS7, qnrS8, qnrS9	https://card.mcmaster.ca/ontology/39224	fluoroquinolone antibiotic	antibiotic target protection

Gene symbol	ThermoFisher (TF) Assay ID	Gene Name	CARD Link	Drug Class(es)	Resistance Mechanism
blaVIM	Ba04646155_s1	subclass B1 metallo-beta-lactamase VIM, blaVIM-1, blaVIM-10, blaVIM-11, blaVIM-12, blaVIM-13, blaVIM-14, blaVIM-15, blaVIM-16, blaVIM-17, blaVIM-18, blaVIM-19, blaVIM-2, blaVIM-20, blaVIM-23, blaVIM-24, blaVIM-25, blaVIM-26, blaVIM-27, blaVIM-28, blaVIM-29, blaVIM-3, blaVIM-30, blaVIM-31, blaVIM-32, blaVIM-33, blaVIM-34, blaVIM-35, blaVIM-36, blaVIM-37, blaVIM-38, blaVIM-39, blaVIM-4, blaVIM-40, blaVIM-41, blaVIM-42, blaVIM-43, blaVIM-44, blaVIM-45, blaVIM-46, blaVIM-47, blaVIM-48, blaVIM-49, blaVIM-5, blaVIM-50, blaVIM-51, blaVIM-52, blaVIM-53, blaVIM-54, blaVIM-55, blaVIM-56, blaVIM-57, blaVIM-58, blaVIM-59, blaVIM-6, blaVIM-60, blaVIM-62, blaVIM-63, blaVIM-64, blaVIM-65, blaVIM-66, blaVIM-67, blaVIM-68, blaVIM-70, blaVIM-72, blaVIM-73, blaVIM-74, blaVIM-75, blaVIM-76, blaVIM-77, blaVIM-78, blaVIM-79, blaVIM-8, blaVIM-9	https://card.mcmaster.ca/ontology/38671	carbapenem, cephalosporin, penem, penam, cephamycin	antibiotic inactivation
